# Glancing Angle Deposition in Gas Sensing: Bridging Morphological Innovations and Sensor Performances

**DOI:** 10.3390/nano15141136

**Published:** 2025-07-21

**Authors:** Shivam Singh, Kenneth Christopher Stiwinter, Jitendra Pratap Singh, Yiping Zhao

**Affiliations:** 1 Department of Physics, Indian Institute of Technology Delhi, Hauz Khas, New Delhi 110016, India; 2 Department of Physics and Astronomy, The University of Georgia, Athens, GA 30602, USA

**Keywords:** glancing angle deposition (GLAD), gas sensors, nanostructured thin films, surface functionalization, noble metal decoration, heterojunctions

## Abstract

Glancing Angle Deposition (GLAD) has emerged as a versatile and powerful nanofabrication technique for developing next-generation gas sensors by enabling precise control over nanostructure geometry, porosity, and material composition. Through dynamic substrate tilting and rotation, GLAD facilitates the fabrication of highly porous, anisotropic nanostructures, such as aligned, tilted, zigzag, helical, and multilayered nanorods, with tunable surface area and diffusion pathways optimized for gas detection. This review provides a comprehensive synthesis of recent advances in GLAD-based gas sensor design, focusing on how structural engineering and material integration converge to enhance sensor performance. Key materials strategies include the construction of heterojunctions and core–shell architectures, controlled doping, and nanoparticle decoration using noble metals or metal oxides to amplify charge transfer, catalytic activity, and redox responsiveness. GLAD-fabricated nanostructures have been effectively deployed across multiple gas sensing modalities, including resistive, capacitive, piezoelectric, and optical platforms, where their high aspect ratios, tailored porosity, and defect-rich surfaces facilitate enhanced gas adsorption kinetics and efficient signal transduction. These devices exhibit high sensitivity and selectivity toward a range of analytes, including NO_2_, CO, H_2_S, and volatile organic compounds (VOCs), with detection limits often reaching the parts-per-billion level. Emerging innovations, such as photo-assisted sensing and integration with artificial intelligence for data analysis and pattern recognition, further extend the capabilities of GLAD-based systems for multifunctional, real-time, and adaptive sensing. Finally, current challenges and future research directions are discussed, emphasizing the promise of GLAD as a scalable platform for next-generation gas sensing technologies.

## 1. Introduction

Gas sensors are vital components in a wide range of applications, including industrial process monitoring, environmental protection, household safety, and medical diagnostics [1,2]. They detect toxic or hazardous gases, prevent accidental leaks, monitor air quality, and can even identify disease biomarkers through breath analysis. In security and defense, they are used to detect chemical warfare agents and explosives, while in law enforcement, they aid in forensic analysis and alcohol detection. The effectiveness of a gas sensor hinges on several performance metrics: sensitivity, selectivity, response time, stability, and environmental robustness [2]. Sensitivity enables detection of trace gases for early warnings, while selectivity minimizes interference from non-target species. Fast response times are crucial for timely decision-making, and stability ensures consistent performance under varying conditions. These performance attributes are intrinsically linked to the underlying sensing mechanisms and fabrication techniques [3,4]. Gas sensors are typically categorized by their transduction mechanisms: electrical, optical, and mechanical. Electrical sensors, especially those based on metal oxide semiconductors (MOS) and conducting polymers, are widely favored for their sensitivity and low cost, though they may require high operating temperatures and exhibit cross-sensitivity [2]. Optical sensors, using techniques like infrared absorption, surface plasmon resonance (SPR), or Raman spectroscopy, offer high selectivity and non-contact operation, albeit with increased complexity and cost [5,6]. Mechanical sensors, such as piezoelectric [7], cantilever-based [8,9], and surface acoustic wave (SAW) devices [10,11], offer high sensitivity and compatibility with MEMS platforms, though long-term stability remains a challenge.

Irrespective of sensing modality, the structure-property relationships of sensing materials are central to performance [12]. Material selection governs response time, stability, and sensitivity. For instance, common MOS materials (SnO_2_, ZnO, TiO_2_) are highly sensitive but often require elevated temperatures [13,14,15,16,17], while conducting polymers allow room-temperature operation but suffer from poor long-term stability [18,19]. Emerging materials such as graphene, carbon nanotubes (CNTs) [20,21], metal-organic frameworks (MOFs) [22,23], and two-dimensional (2D) materials [24,25] offer promising properties, including high surface area, tunable chemistry, and low-temperature responsiveness.

Nanostructuring further enhances gas sensor performance by increasing surface area and active site density, thereby accelerating gas adsorption and diffusion [2,26]. Morphologies such as nanorods (NRs), nanowires (NWs), and hierarchical frameworks promote faster sensor response and recovery [13,14,15,16,17]. Crystallinity also plays a key role: highly crystalline films offer better charge transport, while controlled defect densities (e.g., oxygen vacancies) can enhance reactivity [2,12,13]. Hybrid structures that combine materials such as MOS-graphene composites [27], MOF-functionalized nanostructures [22,23,28], or self-assembled monolayers (SAMs) [29,30] leverage synergistic properties like enhanced conductivity, porosity, and chemical specificity.

Advanced nanofabrication techniques are required to realize these tailored structures [31]. Top-down methods such as photolithography, electron beam lithography (EBL), and focused ion beam (FIB) offer high patterning precision but are costly and time-consuming. Bottom-up approaches, including chemical vapor deposition (CVD), sol-gel synthesis, and hydrothermal growth, are scalable but often suffer from limited control over morphology or reproducibility. Emerging nanopatterning strategies like nanoimprint lithography and block copolymer self-assembly provide new possibilities but involve complex processing. Among these, glancing angle deposition (GLAD) has gained attention as a physical vapor deposition (PVD) method capable of producing vertically aligned, porous nanostructures with tunable geometry [32]. Unlike lithographic techniques, GLAD directly forms nanostructures through physical vapor deposition at oblique angles, inherently eliminating the need for masks or cleanroom-level fabrication environments [33]. Compared to bottom-up methods, it avoids high-temperature reactions and offers better reproducibility [34]. Real-time control over deposition parameters, such as substrate tilt, azimuthal rotation, and deposition rate, enables precise engineering of nanostructure shape, porosity, and orientation, optimizing gas diffusion and adsorption characteristics [35].

GLAD offers several compelling advantages for gas sensor fabrication. The technique supports a broad range of materials, including MOSs (e.g., SnO_2_, ZnO, TiO_2_), noble metals (e.g., Au, Ag, Pt), and composite structures, enabling the combination of semiconducting, plasmonic, and catalytic properties. The resulting columnar, high-surface-area morphologies facilitate gas transport, enhance signal stability, and reduce cross-sensitivity. Furthermore, post-deposition functionalization, such as coating with MOFs or molecular layers, can fine-tune selectivity and sensing specificity. Crucially, GLAD is scalable and cost-efficient. It can be implemented on standard PVD systems, supports batch processing over large substrates, and eliminates the need for expensive precursors or complex process steps, making it ideal for commercial sensor development [32].

**Figure 1:**
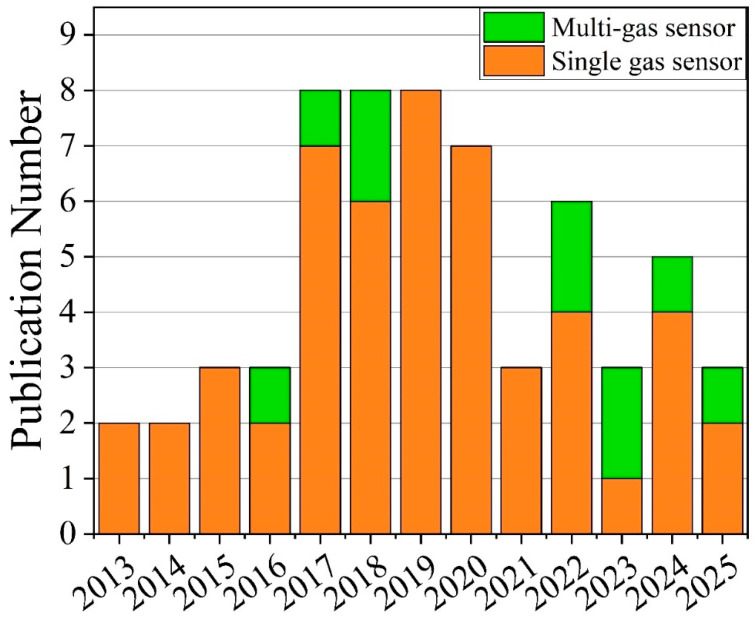
Number of GLAD-based gas sensor publications by year via Web of Science, accessed May 2025.

Figure 1 presents the annual publication trend for GLAD-based gas sensors over the past 12 years. Despite the demonstrated capabilities of GLAD, the number of related publications remains modest, peaking below ten papers per year. A brief increase occurred from 2017 to 2019, but subsequent output has stagnated. This indicates that GLAD remains underutilized in gas sensing, representing an opportunity for innovation in material design, structural engineering, and application-specific integration. Given this gap, there is a clear need for a comprehensive review that consolidates existing knowledge and outlines future research directions.

This review is organized as follows: Section 2 introduces the GLAD technique and its influence on nanostructure morphology. Section 3 explores the role of GLAD in various gas sensing modalities, highlighting the impact of structural and material design. Section 4 discusses emerging strategies for advancing GLAD-based sensors through hybrid architectures, functional coatings, and compositional engineering. Finally, Section 5 summarizes key insights and outlines future opportunities for developing scalable, multifunctional, and intelligent gas sensing technologies using GLAD.

## 2. Glancing Angle Deposition Technique

### 2.1. GLAD Configuration and Principles

**Figure 2:**
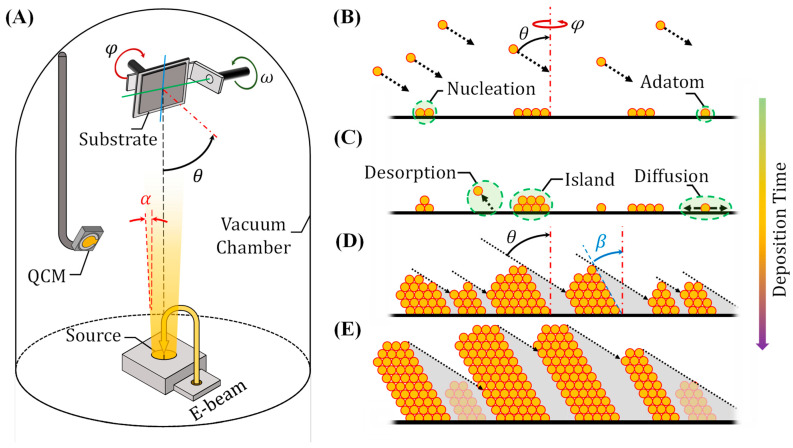
(**A**) A typical GLAD configuration in an electron-beam evaporation system. NR formation mechanisms during GLAD as a function of deposition time: (**B**) Deposition and random nucleation on the substrate; (**C**) island formation and adatom desorption; (**D**) tilting NR formation and self-shadowing effect; and (**E**) self-shadowing induced competing growth of NRs.

GLAD is a highly versatile nanofabrication technique that modifies conventional PVD by introducing an extreme substrate tilt angle, typically θ>70°, as illustrated in Figure 2A [32,36]. This high tilt results in oblique vapor flux, inducing anisotropic growth and enabling the formation of vertically aligned, porous nanostructures well-suited for gas sensing applications. Unlike standard PVD, which forms dense and uniform films, GLAD leverages directional deposition to produce columnar geometries with tunable morphology and porosity.

A typical GLAD setup (Figure 2A) consists of a vacuum chamber equipped with an evaporation or sputtering source, a substrate holder capable of precise tilt (θ) and azimuthal (φ) rotation, and a quartz crystal microbalance (QCM) for real-time monitoring of deposition rate and film thickness. The key to GLAD is maintaining a long mean free path in high vacuum conditions, which preserves the directionality of the vapor flux and facilitates self-shadowing effects during deposition. Nanorod (NR) formation in GLAD proceeds through four key stages (Figure 2B–E): (1) Initial nucleation (Figure 2B): Vapor atoms adsorb onto the tilted substrate surface and diffuse to form small nuclei. Surface diffusion governs the distribution and aggregation of adatoms. (2) Island formation (Figure 2C): These nuclei grow into isolated islands or clusters. Simultaneously, weakly bound adatoms may desorb, and a balance between adsorption, diffusion, and re-evaporation stabilizes island formation. (3) NR growth via self-shadowing (Figure 2D): As islands gain height, they cast shadows over neighboring regions due to the steep incidence angle. This shadowing limits deposition to neighboring areas, resulting in tilted or slanted NRs. (4) Competing NR growth (Figure 2E): Taller structures continue to dominate the vapor flux, while shorter ones fall into shadow and cease growing [37]. This leads to high-aspect-ratio, tilted NRs (tilting angle β) with directional anisotropy.

The interplay between adatom diffusion and geometric shadowing dictates the resulting morphology. The adatom diffusion length $L_{d}$, which reflects how far atoms can travel before being immobilized, depends on deposition rate (*R*), diffusion coefficient (*D*), and substrate temperature (*T*). It is given by [38]

(1) $$L_{d}=\sqrt{\frac{2D}{R}}=\sqrt{{2D}_{0}\exp\left(-\frac{E_{a}}{k_{B}T}\right)/R},$$

where $D_{0}$ is the pre-exponential diffusion constant, $E_{a}$ is the activation energy, and $k_{B}$ is Boltzmann’s constant. At early stages (Figure 2B), diffusion occurs along the flat substrate, so $D_{0}$ and $E_{a}$ correspond to substrate-adatom specific parameters. In later stages (Figure 2D,E), it occurs along the growing NR surfaces and is governed by the self-diffusion properties of the deposition material.

Self-shadowing is a geometric effect governed by the incident angle (θ) and nanostructure height (*h*), with the shadowing length $L_{s}$ approximated as [32],

(2) $$L_{s}=h\;tan\left(\theta \right).$$

When $L_{s}>L_{d}$, adatoms cannot reach shaded regions, and porous nanocolumns form. Conversely, if $L_{s}<L_{d}$, diffusion dominates and adatoms can fill in shadowed areas, leading to denser films with reduced porosity. Thus, lower substrate temperatures, which reduce adatom mobility, are preferred in GLAD to enhance separation and porosity. Structure zone models (SZMs), such as the Movchan-Demchishin model, describe how film morphology changes based on the ratio of substrate temperature to the melting point of the material being deposited, and are often used to guide the optimization of GLAD conditions [36,39].

In practice, the vapor flux is not perfectly collimated but has an angular spread, described by the solid angle Ω, which depends on the half angle (α) of the flux cone (see Figure 2A),

(3) Ω=2π(1−cosα).

A narrow flux (small α) promotes stronger shadowing and more distinct nanostructures, while broader fluxes reduce separation and increase film density.

Together, these factors, deposition angle, substrate temperature, flux directionality, and surface diffusion, allow GLAD to produce a diverse array of nanostructures with tunable geometry and porosity. This makes it uniquely suited for applications where high surface area and tailored pathways for gas diffusion are essential, such as in resistive and optical gas sensors.

### 2.2. Tailoring the Morphology of Nanostructures via GLAD

**Figure 3:**
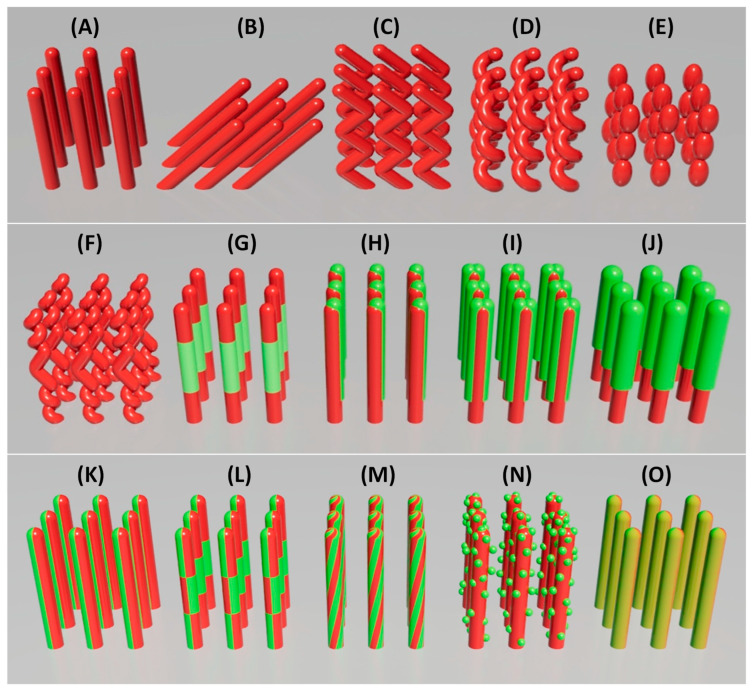
Schematics of different nanostructures realized by GLAD: Morphological sculpturing: (**A**) Vertically aligned NRs; (**B**) tilted NRs; (**C**) Zig-zag NRs; (**D**) Helical NRs; (**E**) Beaded NRs, and (**F**) Helical-zigzag multilayer NRs. Heterostructure NRs: (**G**) Multilayered NRs; (**H**) Side-coated NRs; (**I**) Sandwiched NRs; and (**J**) Core-shell NRs. Composite NRs through co-deposition: (**K**) Janus NRs; (**L**) Checkboard NRs; (**M**) Double helices NRs or twisted “candy cane” NRs; (**N**) NP decorated NRs; and (**O**) Doped NRs.

GLAD offers unique control over nanostructure geometry and composition through manipulation of deposition parameters such as incident angle (θ), azimuthal rotation (φ), material selection, and deposition sequence [34,40,41,42]. These capabilities enable the design of nanostructures with tunable porosity, surface area, and functionality as shown in Figure 3. The primary design strategies fall into three categories: morphological sculpturing, heterostructure formation, and composite co-deposition.

**Morphological Sculpturing:** By adjusting the vapor incidence and substrate rotation, GLAD enables the fabrication of complex geometries [34,42,43,44]:

- *Vertically aligned NRs* (Figure 3A): Achieved by fast, continuous rotation at a fixed θ, producing symmetric structures with uniform exposure.
- *Tilted NRs* (Figure 3B): Formed by fixing θ without azimuthal rotation, leading to slanted rods due to preferential growth on the flux-facing side, commonly referred to as oblique angle deposition (OAD).
- *Zig-zag NRs* (Figure 3C): Generated by alternating φ in discrete steps during deposition, creating kinked structures that increase surface complexity.
- *Helical NRs* (Figure 3D): Produced via slow, continuous azimuthal rotation, wrapping the material into a spiral trajectory. The pitch is controlled by rotation speed relative to the deposition rate.
- *Beaded NRs* (Figure 3E): Formed by modulating θ or φ dynamically during growth, resulting in periodic constrictions that enhance surface area.
- *Helical-zigzag NRs* (Figure 3F): Constructed by combining rotation profiles mid-growth, producing multilayered architectures suitable for multifunctional applications.

These sculptured geometries directly influence gas transport, adsorption kinetics, and sensor response by enhancing surface accessibility and diffusion pathways.

**Heterostructure Formation**: GLAD supports the formation of compositional heterostructures by sequentially introducing different materials [40,41].

- *Multilayered NRs* (Figure 3G): Constructed by alternating material sources during growth, forming vertical heterojunctions along the rod axis. The number of layers and the thickness of each segment can be precisely controlled by tailoring functional properties. For example, WO_3_/TiO_2_ layers can enhance photocatalytic activity [45].
- *Side-coated NRs* (Figure 3H): Achieved by depositing a secondary material at a distinct angle (θ or φ), coating one side of the rods. The extent of side coverage can be tuned by adjusting the deposition angle θ of the second source. This asymmetry supports directional sensing or catalytic activity.
- *Sandwiched NRs* (Figure 3I): Fabricated through a two-step side coating by rotating the substrate 180° azimuthally after the first side-coating, leading to symmetric dual-side heterostructures.
- *Core-shell NRs* (Figure 3J): Formed by post-deposition coating with GLAD [46] or atomic layer deposition (ALD) [47], resulting in uniform shells for passivation or catalytic enhancement.

These heterostructural strategies can be combined with morphological sculpturing to produce nanostructures with both topological and compositional complexity. For instance, a square-helical heterostructure composed of alternating layers of Si and Ni was fabricated through precise azimuthal rotation and sequential material deposition, enabling anisotropic magnetic properties suitable for micromotor applications [48]. In another approach, Ag-coated Si NRs were assembled into a segmented helical configuration, forming a catalytic nanomotor capable of self-propulsion in liquid media [49]. Such heterostructures enhance sensing by leveraging interfacial phenomena like band bending, charge transfer, and catalytic synergies.

**Composite structures via co-deposition:** Simultaneous deposition of multiple materials enables unique composite nanostructures [40,41].

- *Janus NRs* (Figure 3K): Formed using two vapor sources from opposite directions, producing rods with chemically distinct sides [50,51].
- *Checkerboard NRs* (Figure 3L): Achieved by rotating the substrate 180° azimuthally and alternatively with the same deposition configuration for Janus NRs, resulting in segmental composition variations [51].
- *Double helices or “candy cane” twisted NRs* (Figure 3M): Created by co-deposition during helical rotation, producing twisted, asymmetric rods for chiral or plasmonic applications.
- *Nanoparticle (NP)-decorated NRs* (Figure 3N): Fabricated via co-deposition or post-deposition sputtering, enhancing catalytic activity and surface reactivity [52,53] (See Section 3.1.4).
- *Doped NRs* (Figure 3O): Produced by introducing dopants during deposition [54]. Controlled doping gradients can tailor local conductivity and sensitivity [55].

These composite structures offer fine control over the electrical, chemical, and catalytic properties of the sensing layer while retaining the high surface area and porosity characteristic of GLAD films.

In summary, the flexibility of GLAD in both structural sculpting and compositional tuning provides a powerful toolkit for engineering nanostructures with enhanced gas sensing performance. These design strategies allow researchers to systematically optimize surface area, porosity, anisotropy, and chemical specificity to meet the demands of next-generation sensor applications.

### 2.3. The Advantages of GLAD Structures for Gas Sensors

The performance of nanostructured gas sensors is fundamentally determined by a combination of structural and material parameters that influence gas adsorption, surface interaction, and charge transport [56]. Among these, high surface area, controllable porosity, tunable electronic structure, and selective surface reactivity are especially critical. GLAD provides an effective strategy to tailor these properties by enabling precise control over nanostructure geometry, alignment, and composition [57]. This section details how GLAD-fabricated architecture enhances gas sensor functionality by addressing key design aspects, including surface area and porosity, inter-column connectivity, material selection, and crystallinity.

#### 2.3.1. Surface Area and Porosity

GLAD enables the fabrication of porous films with high surface-to-volume ratios, which increase the number of active sites for gas adsorption. Porosity and surface area are primarily controlled by deposition angle, film thickness, NR topology, deposition rate, material properties, and substrate temperature.

**Figure 4:**
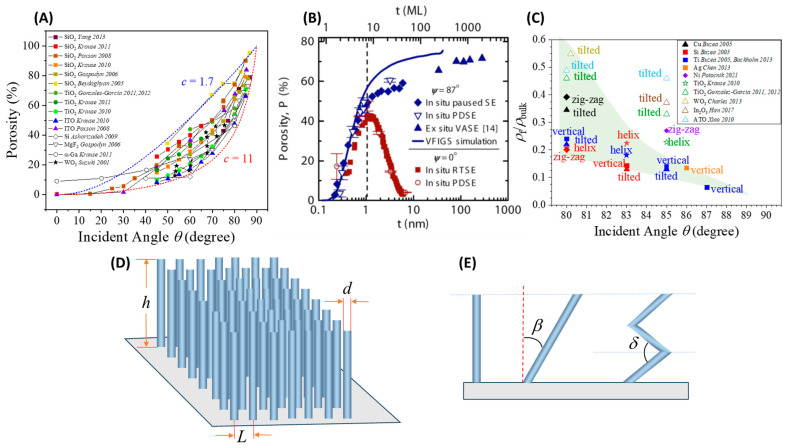
(**A**) Reported porosity values of GLAD films as a function of deposition angle (*θ*) from the literature [58,59,60,61,62,63,64,65,66,67]. (**B**) Evolution of film porosity with deposition time for θ=0° and 87°, where ML on top *x*-axis refers to monolayer [68]. (**C**) Summary of the ratio of GLAD film density $\rho_{f}$ to the bulk material density $\rho_{bulk}$ as a function of deposition angle and NR morphology [60,64,65,69,70,71,72,73,74,75]. (**D**) Schematic illustration of a regular array of GLAD-grown NRs. (**E**) Comparative schematic of NR geometries, straight, tilted, and zig-zag, all with identical height.

Among all parameters, θ has the most direct impact on film porosity. Figure 4A compiles experimental data of porosity (P) versus θ from various materials deposited via GLAD, showing a clear monotonic increase with θ regardless of materials and deposition methods used [58,59,60,61,62,63,64,65,66,67]. Beyond θ≈70°, self-shadowing becomes dominant, leading to the formation of well-separated nanocolumns. Poxson et al. [58] proposed a model to describe this trend,

(4) $$P=\frac{\theta \tan\theta }{c+\theta \tan\theta },$$

where c is a material-dependent constant. In Figure 4A, the blue and red dashed curves represent the model with *c* = 1.7 and *c* = 11, respectively. The experimental data fall within these two curves, highlighting the general applicability of the model.

Increasing deposition time increases NR height, aspect ratio, and available surface area. However, beyond a critical thickness, mechanical instability can cause rod merging or collapse, reducing porosity. Amassian et al. [68] investigated this progression and identified a critical thickness threshold for the onset of shadowing-dominated growth at various deposition angles. Figure 4B presents the evolution of film porosity *P* as a function of film thickness *t* for amorphous silicon (a-Si) films deposited at θ=0° and θ=87°. The figure includes data from various in situ and ex situ measurement techniques: real-time spectroscopic ellipsometry (RTSE) for normal incidence films (solid red squares), paused in situ ellipsometry for GLAD films (solid blue diamonds), and ex situ ellipsometry (solid blue triangles). Additional markers, such as hollow red circles and blue inverted triangles, represent post-deposition and paused data points, respectively, for cross-validation. The key observation is the contrasting porosity trends between the two deposition angles. At θ=0°, porosity initially increases slightly (reaching ~45% around 1.2 nm thickness) but then decreases toward zero as the film becomes continuous. In contrast, for GLAD films, porosity rises continuously and asymptotically with increasing film thickness, indicating the onset and dominance of the self-shadowing effect early in the growth process. This divergence beyond ~1 nm thickness marks the transition to shadowing-dominated growth, highlighting the distinct structural evolution enabled by GLAD.

Although NR morphology (e.g., helical, zigzag) has a lesser effect on porosity than θ, it significantly increases surface complexity and gas-accessible area. As illustrated in Figure 4C, films with different morphologies fabricated under similar conditions (e.g., material type and deposition angle) show comparable density and porosity, suggesting that tilt angle (i.e., shadowing length) and material composition are the primary factors influencing porosity [64,65,69,70,71,72,73,74,75]. Notably, vertical columns are generally denser than tilted structures made from the same material. Meanwhile, zigzag and helical NRs exhibit similar densities, and metal oxide nanostructures tend to be denser than those composed of pure metals.

Consider a simplified model, illustrated in Figure 4D, where we assume that GLAD NRs are uniformly arranged in a square lattice on a substrate. Each NR is assumed to be a solid cylinder with height h, diameter d, and arranged with a center-to-center spacing L. In this configuration, the ratio of the total surface area of the NR array ($A_{NR}$) to the projected flat substrate area $(A_{0}$) can be expressed as

(5) $$\frac{A_{NR}}{A_{0}}=1+\pi \frac{d^{2}}{L^{2}}\gamma ,\;$$

where γ=h/d represents the aspect ratio of the NRs. If we assume d≈0.5L, Equation (5) can be simplified as $\frac{A_{NR}}{A_{0}}\approx 1+\frac{\pi \gamma }{4}$. For an average aspect ratio γ=12, this yields a surface area enhancement factor of approximately 10, and for γ=20, the ratio increases to about 17.

It is important to note that Equation (5) assumes each NR is a solid, non-porous cylinder. However, microscopic analyses of GLAD-grown NRs reveal the presence of nanoscale pores within the NRs, contributing additional internal surface area [32]. To account for this porosity, the surface area ratio can be modified as,

(6) $$\frac{A_{NR}}{A_{0}}=1+\kappa \frac{\pi d^{2}}{L^{2}}\gamma ,$$

where κ>1 is a correction factor. For a zig-zag NR array fabricated on a square lattice, each NR can be modeled as a sequence of N cylindrical segments of length l and diameter d, joined at a bending angle δ. The surface area per unit substrate area is then enhanced by a factor

(7) $$\frac{A_{zig-zag}}{A_{0}}=1+\kappa \frac{\pi d^{2}}{L^{2}}N\gamma^{\prime },$$

where $\gamma^{\prime }=l/d$ is the segment aspect ratio. As shown in Figure 4E, now consider a tilted NR array with a tilt angle β, and let the total film thickness be the same as the height h of a straight NR array. The length of each tilted NR must satisfy l=h/cosβ, yielding

(8) $$\frac{A_{tilt}}{A_{0}}=1+\kappa \frac{\pi d^{2}}{L^{2}}\frac{1}{\cos\beta }\gamma .$$

This shows that for the same h, tilted NRs provide more surface area than vertically aligned rods. Since the tilt angle β generally increases with θ, larger θ should lead to a higher surface area. For example, if β=60°, $\frac{1}{\cos\beta }$ will give 2 times more surface area compared to straight NR array. For zig-zag NRs of the same total height as straight NRs, the surface area factor becomes,

(9) $$\frac{A_{zig-zag}}{A_{0}}=1+\kappa \frac{\pi d^{2}}{L^{2}}\frac{1}{\sin\frac{\delta }{2}}\gamma .$$

Notably, when $\beta +\frac{\delta }{2}=\frac{\pi }{2}$, theoretically the zig-zag and tilted NR arrays yield equivalent surface area enhancement ($\frac{A_{zig-zag}}{A_{0}}=\frac{A_{tilt}}{A_{0}}$).

These analytical models assume idealized geometries, i.e., perfectly cylindrical NRs with uniform tilts or bends and even spatial distribution, which are common simplifications used in power-law scaling analyses of nanocolumnar growth during oblique angle deposition [76]. In practice, however, growth dynamics, shadowing effects, surface diffusion, and vapor flux variations lead to deviations, producing features like surface roughness, voids, grain boundaries, and fan-out effect that affect the actual surface area [32,77]. As a result, the correction factor κ is not universal but varies with NR morphology, deposition conditions, and material system [78]. For instance, straight NRs differ in porosity from zig-zag or tilted ones, complicating direct comparisons. Thus, theoretical predictions must be calibrated against experimental data such as Brunauer–Emmett–Teller (BET) measurement, electron microscopy, or gas adsorption to ensure accuracy.

Deposition rate is a key parameter influencing nanocolumn morphology and porosity in GLAD processes, although the exact way it modulates these properties appears to be more complex than traditionally understood. Lower deposition rates are generally thought to allow adatoms more time to diffuse, potentially decreasing column separation, porosity, and surface roughness [79]. However, experimental studies have shown that this relationship does not always hold, suggesting that additional factors may complicate or even reverse the expected trends [80]. This warrants further experimental study, as suggested by Buzea et al. [75], who emphasized the importance of deposition rate along with pressure and substrate temperature in shaping film morphology and advocated its further study to improve column uniformity and scaling behavior.

Substrate temperature controls the adatom diffusion length, where the diffusion coefficient *D* increases with temperature [81]. At high temperatures, atoms can migrate into shadowed regions, reducing porosity. At low temperatures, mobility is restricted, preserving shadowed areas and enhancing column separation [82]. Kay et al. [83] showed that annealing or increasing substrate temperature reduced internal voids and caused structural coalescence, leading to lower surface area and reduced gas diffusion pathways.

The morphology and porosity of GLAD- or OAD-grown nanocolumnar films are strongly governed by material-dependent properties such as surface energy, vapor pressure, sticking probability, surface diffusion kinetics, and crystal structure [54,79,84,85]. These factors influence adatom mobility, which in turn determines column geometry, density, and porosity [86]. Karabacak et al. [87] demonstrated that column diameter scales with height via a material-dependent power law, where the growth exponent *p* reflects the balance between shadowing and diffusion. Materials with low surface diffusivity (e.g., Si, W) form broader and more porous columns, whereas those with high mobility (e.g., Cu, Co) yield denser structures. Simulations confirmed that increasing diffusivity reduces *p* from ~0.5 (pure shadowing) to ~0.31 (diffusion-influenced), underscoring the critical role of material properties in porosity evolution. These findings align with Buzea et al. [88], who observed that Si nanocolumns exhibit higher porosity and roughness than metals like Ag or Cr due to reduced adatom mobility. Material influence extends to the column tilt angle β. Zhu et al. [37] reported that β varies significantly with material type under identical deposition conditions. Using a fan-out model, they introduced a material-specific fan angle correlated with melting temperature for elements and heat of formation for compounds. Low-mobility materials such as Si and TiO_2_ exhibited steeper tilt angles and higher porosity due to enhanced shadowing.

#### 2.3.2. Connectivity or Percolation

In addition to porosity, connectivity is a critical parameter in GLAD-fabricated nanostructures, particularly for resistive sensors, as it governs charge transport and electrical percolation [32]. Connectivity arises not only from the intrinsic conductivity of the material but also from the geometrical arrangement and overlap of nanocolumns, which are determined by deposition parameters such as angle, rotation speed, deposition method, and post-deposition annealing [32,89]. As nanocolumns become more isolated, charge carrier pathways become discontinuous, increasing film resistivity. Thus, resistivity ($\rho_{res}$) serves as a quantitative indicator of connectivity: low $\rho_{res}$ suggests good percolation, while high $\rho_{res}$ indicates poor structural continuity [32,74,90,91,92,93,94,95,96]. 

**Figure 5:**
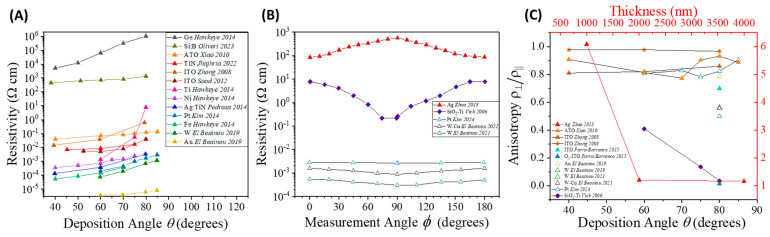
(**A**) Reported resistivity ρ of GLAD films as a function of θ for various materials, compiled from the literature [32,74,90,91,92,93,94,95,96]. (**B**) Resistivity of GLAD films deposited by sputtering (hollow symbols) and e-beam evaporation (filled symbols) at high deposition angles (θ > 80°) as a function of measurement angle (ϕ). The symbol shapes indicate NR morphologies: ▲ for tilted, ♦ for zigzag, and ★ for helical nanostructures [95,98,99,100]. (**C**) Anisotropic resistivity ratio ($\rho_{⊥}/\rho_{∥}$) of GLAD nanostructures plotted against θ, including thickness-dependent data for Ag films. The same symbol designations from (**B**) are used [74,90,95,96,98,99,100,101].

Among these parameters, deposition angle (θ) plays a pivotal role because it directly governs film porosity, nanocolumn tilt, and inter-column connectivity via geometric shadowing effects, affecting film resistivity. At small angles (θ≤50°), the incoming vapor flux still maintains partial access to lateral growth sites, enabling denser and more connected structures; thus $\rho_{res}$ remains largely unchanged in DC-sputtered Cr films [97]. However, as shown in Figure 5A, when θ increases, a monotonic rise in $\rho_{res}$ is observed across a wide range of materials, including metals and conductive oxides, regardless of the deposition method [32,74,90,91,92]. Based on the known porosity–angle relationship, Sood et al. [94] proposed an empirical model,

(10) $$\rho_{res}=\rho_{res}^{b}{\left(\frac{1}{1-P}\right)}^{s},$$

where $\rho_{res}^{b}$ is the bulk resistivity and the exponent s is a structure factor reflecting the morphological contribution to resistivity. Interestingly, $\rho_{res}$ at a fixed θ does not always follow trends predicted by bulk resistivity. For example, while for the bulk resistivity Ge>ITO>TiN>Ti>Fe>Ni, experimental data at θ=60° (Figure 5A) show Ge>ITO>TiN>Ti>Ni>Fe. This suggests that geometry, rather than intrinsic material properties, dominates electrical behavior in GLAD films. At low θ, more uniform vapor flux yields denser films with stronger inter-column contact. At higher θ, oblique vapor incidence enhances self-shadowing, producing isolated columns and reduced connectivity. 

In addition to the total resistivity and resistivity anisotropy, the directional dependence of electrical transport provides further insight. As shown in Figure 5B, anisotropy is measured by varying the in-plane measurement angle (ϕ). For most materials, the $\rho_{res}-\phi$ relationship exhibits two-fold symmetry, decreasing from ϕ=0° (parallel to flux) to 90°, then increasing back to 180° [95,99,100], with Ag as an exception [98]. The anisotropy ratio $\frac{\rho_{res}^{⊥}}{\rho_{res}^{∥}}$, where ⊥ and ∥ refer to ϕ=90° and 0°, respectively, quantifies this effect: lower ratios imply greater anisotropy.

As shown in Figure 5B, sputtered films (hollow symbols), such as Pt, W, and W-Cu, exhibit lower resistivity and weaker anisotropy than e-beam evaporated Ag films, despite higher bulk resistivities. This inversion highlights the geometric influence. Sputtering, due to its larger divergence angle (α), enhances lateral growth and inter-column contact. Conversely, e-beam evaporation under low pressure and large source-to-substrate distances yields more isolated NRs with higher resistivity. Sputtering pressure further modulates resistivity [100,102]. At low pressures, columns grow longer and more separated, yielding low resistivity along the flux but high anisotropy. High-pressure conditions lead to denser films with increased resistivity and reduced anisotropy due to limited lateral diffusion and higher defect density.

Figure 5B,C illustrate how NR geometry dictates anisotropy. Tilted NRs (▲), Pt [95], W [96,100], and W–Cu [100] show moderate anisotropy with ratios of 0.2–0.93, while zig-zag SiO_2_–Ti bilayers (♦) [99] reach extreme anisotropy ratios as low as 0.03. Helical ITO structures (★) [90] exhibit near-isotropic behavior, with ratios close to 1. This trend reflects a clear geometric effect: helices maintain 3D connectivity across directions, while zigzag structures, despite their less directional appearance, suppress lateral coalescence due to abrupt growth direction changes, dramatically reducing cross-column links.

Even more so, Figure 5C reveals that electrical anisotropy does not rise or fall smoothly with deposition angle; instead, each morphology also follows a distinct angle-dependent anisotropy trend. Helices retain isotropy across tilt angles, tilted rods show non-monotonic anisotropy, and zigzag structures exhibit a sharp anisotropy increase with θ from 60° to 80°, likely due to disrupted axial pathways and inhibited lateral merging. A particularly striking example is the “inverted” anisotropy in silver NRs [98]. A 1000 nm Ag NR film shows enhanced conduction along the tilt direction ($\frac{\rho_{res}^{⊥}}{\rho_{res}^{∥}}$ ≈ 6), due to smooth surfaces and minimal lateral overlap. Extending the growth to 4000 nm increases rod tip fanning, restoring cross-column contact and reducing the ratio to ~1.16 approaching isotropy. These results emphasize that both morphology and growth length dictate how a deposition angle translates into anisotropic electrical transport behavior.

#### 2.3.3. Material Selection

Table 1 summarizes the key attributes and trade-offs of each PVD method when used in GLAD-based fabrication, highlighting their influence on morphology, porosity, and crystallinity. The performance of GLAD-fabricated gas sensors depends critically on the selection and engineering of active materials that possess high surface reactivity, tunable electronic properties, and compatibility with nanoscale structuring [103]. Metal oxides (e.g., ZnO, SnO_2_, TiO_2_, WO_3_, In_2_O_3_, CuO) continue to dominate the field due to their robust redox activity, ease of synthesis, and adaptability to GLAD processes [104,105]. 

These materials offer tunable band structures, oxygen vacancy concentrations, and surface chemistries, which collectively determine gas adsorption behavior, operating temperature, and sensing response [103,104].

The compatibility of GLAD with a broad spectrum of materials, including elemental metals, semiconductors, binary and ternary oxides, nitrides, fluorides, and sulfides, arises from its foundation in physical vapor deposition (PVD). If the material can be vaporized under vacuum, it can be structured into porous nanocolumnar morphologies using GLAD. Various PVD techniques such as thermal evaporation, e-beam evaporation, sputtering (RF/DC), pulsed laser deposition (PLD), and ion beam-assisted deposition (IBAD) have been adapted for GLAD, each offering unique advantages in deposition rate, energy, and material compatibility. Because GLAD only requires a programmable tilt/rotation stage added to an otherwise standard PVD tool, its incremental capital expense is modest; nevertheless, a full cost-of-ownership analysis relative to other fabrication routes remains an open topic for future work.

A broad range of materials have been successfully nanostructured using GLAD (see Table 2). These include element metals such as Ag, Au, Cu, Cr, Co, and Al, as well as semiconductors, nitrides (e.g., TiN, CrN), fluorides (e.g., MgF_2_, CaF_2_), sulfides (e.g., SnS, ${In}_{2}S_{3}$), and even antimony- or germanium-based compounds [32,74,107,108]; and multicomponent systems, including doped and co-deposited variants (see Figure 3). However, reactive or low-melting-point materials, such as pure Pb, Cs, or Rb, are less commonly reported [109,110].

Not only is GLAD effective in producing nanostructured MOSs, but their performance can be further tailored by (i) doping (C-doped WO_3_) [111] or decorating post-deposition with NPs (e.g., Au, Pt, Pd, Rh) [112,113,114,115,116,117,118,119] to enhance selectivity and sensitivity via Schottky junctions [120,121], Fermi level modulation [122], or spillover catalysis [123]. Ultimately, the material system chosen for GLAD gas sensors should be tailored to the target analyte and sensing mechanism.

**Table 2:** Materials fabricated by GLAD.

Material	Ref.	Material	Ref.	Material	Ref.	Material	Ref.	Material	Ref.
***Element***									
Ag	[32]	Co	[32]	Mg	[32]	Pt	[32]	Ta	[32]
Al	[32]	Cr	[32]	Mn	[32]	Rh	[119]	Te	[32]
Au	[32]	Cu	[32]	Nb	[32]	Ru	[32]	Ti	[32]
Bi	[124]	Fe	[32]	Ni	[32]	Se	[32]	W	[32]
C	[32]	Ge	[32]	Pd	[32]	Si	[32]	Zn	[125]
***Two Element—Oxide***									
${As}_{2}O_{3}$	[32]	${Fe}_{2}O_{3}$	[32]	${MoO}_{3}$	[32]	${SnO}_{2}$	[126]	${WO}_{3}$	[32]
${CeO}_{2}$	[32]	${Gd}_{2}O_{3}$	[127]	NiO	[128]	${Ta}_{2}O_{5}$	[32]	${ZrO}_{2}$	[32]
CuO	[129]	${HfO}_{2}$	[32]	${RuO}_{2}$	[32]	${TiO}_{2}$	[32]	ZnO	[32]
${Cu}_{2}O$	[130]	${In}_{2}O_{3}$	[73]	SiO	[32]	$V_{2}O_{5}$	[131]		
${Er}_{2}O_{3}$	[132]	MgO	[32]	${SiO}_{2}$	[32]	${VO}_{2}$	[133]		
***Two Element—non-Oxide***									
${CaF}_{2}$	[32]	CrN	[32]	InN	[32]	Ta3N5	[32]	ZnS	[32]
CdS	[134]	${GeSe}_{2}$	[32]	${MgF}_{2}$	[32]	TiC	[32]		
CdTe	[135]	${In}_{2}S_{3}$	[32]	SnS	[108]	${WSe}_{2}$	[136]		
***Three Element—Oxide***						***Three Element—non-Oxide***			
**ATO**	[32]	CrN_1−x_O_x_	[137]	W_x_Si_y_O_x_	[32]	GeSbSn	[32]	TiAlN	[32]
${BiVO}_{4}$	[32]	ITO	[32]	$Y_{2}O_{3}:Eu$	[32]	${Sn}_{3}{Sb}_{2}S_{6}$	[138]	TiZrV	[32]

#### 2.3.4. Crystal Quality

Crystallinity plays a pivotal role in the performance of gas sensors by influencing charge carrier mobility, chemical reactivity, and long-term structural stability [139,140,141]. In GLAD-fabricated films, the unique oblique deposition geometry and limited surface diffusion during growth often result in amorphous or nanocrystalline structures. Therefore, understanding and controlling the crystal quality of GLAD nanostructures through deposition parameters and post-deposition treatments is essential to optimize sensor functionality. The grain size orientation, and phase composition of GLAD NRs can be tailored by modulating deposition angle, substrate temperature, material choice, and annealing conditions.

**Figure 6:**
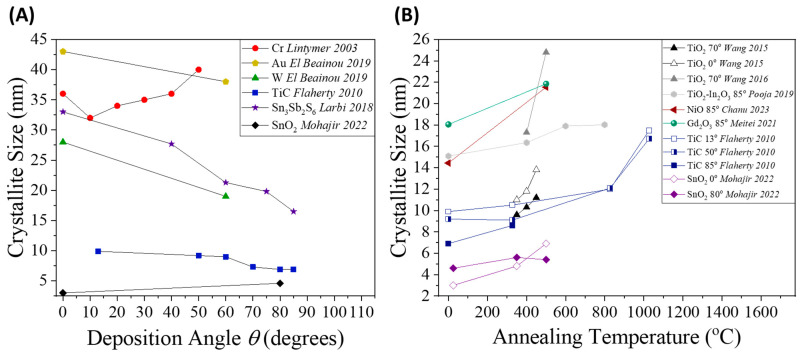
(**A**) Crystallite grain size of as-deposited films as a function of deposition angle [96,97,138,142,143]. (**B**) Evolution of crystallite grain size with increasing annealing temperature for non-GLAD (hollow symbols) and GLAD-deposited (filled symbols) nanostructures [128,142,143,146,147,148,149]. Crystallite sizes are estimated from XRD peak broadening using Scherrer’s equation.

As seen in Figure 6A, at higher *θ*, shadowing dominates and adatom diffusion is restricted, often leading to finer grains and increased amorphous content [96,138,142]. However, the impact of on crystallinity is material dependent. For example, studies on Cr and SnO_2_ showed that grain size increases with higher *θ* [97,143].

Thermal annealing is widely employed to enhance crystallinity, induce phase transitions, and relieve internal stresses [32,144]. Figure 6B shows the evolution of crystallite size in various GLAD-deposited films as a function of annealing temperature. As expected, grain size increases with temperature due to enhanced atomic mobility and grain coalescence [145]. Among the as-deposited films, SnO_2_ deposited at θ=0° exhibits the lowest crystallinity, while Gd_2_O_3_ deposited at θ=85° shows the highest crystallinity, highlighting material-dependent and θ-dependent crystallization behavior [143,146]. For TiO_2_, TiC, and SnO_2_, higher deposition angles lead to more rapid grain growth with temperature. Notably, TiO_2_ deposited at θ=70° shows a sharp increase in grain size over a relatively small temperature increment. This accelerated growth is attributed to the higher porosity of GLAD structures at larger θ, which enhances surface diffusion during annealing. Since surface atoms are significantly more mobile, owing to a surface melting point roughly one-third that of the bulk, grain growth becomes more efficient, resulting in improved crystallinity under identical thermal conditions. However, some materials deviate from this trend. GLAD-deposited SnO_2_ NRs and heavily oxidized TiO_2_–In_2_O_3_ coaxial NRs exhibit slower crystallite growth until the highest annealing temperatures tested [143,147].

In addition, the crystal phase of a MOS plays a critical role in determining its gas sensing behavior. Different phases can exhibit distinct surface chemistries, catalytic activities, and electronic properties that affect gas adsorption and charge transport. For instance, in TiO_2_, the rutile phase is often favored for gas sensing due to its higher density of surface-active sites and greater surface roughness, which facilitate gas adsorption and electron exchange [150,151]. However, anatase may offer better electron mobility and interfacial conductivity matching for composite-based systems, making them interesting candidates for novel gas sensing applications [152,153]. Generally, crystallization in GLAD-deposited MOS films initiates above 400–500 °C, and while annealing time ensures phase completion, the temperature largely determines the resulting crystal structure. For example, tilted TiO_2_ NRs deposited at θ=60° and 80° consistently transform into the anatase phase after 2 h of annealing between 450–650 °C [154], similar to zigzag TiO_2_ NRs annealed for 5 h at 450–600 °C [155]. In contrast, Au-decorated vertical TiO_2_ columns annealed at 950 °C for 1 h transition into a mixed anatase–rutile phase, with rutile becoming dominant [149,156]. Other oxides exhibit similar material-dependent behavior. NiO and Gd_2_O_3_ readily adopt the cubic phase after just 1 h at 500 °C, while In_2_O_3_ becomes cubic after 4 h at the same temperature [127,128,157]. VO_2_ demonstrates rapid crystallization, forming its monoclinic phase within 30 min at 500 °C [106]. The role of dopants is also evident in phase tuning. Undoped WO_3_ nanostructures form the orthorhombic phase at 400 °C [158], whereas Ni co-deposition shifts crystallization toward the monoclinic phase at 500 °C [159]. This suggests that compositional modification can be strategically employed to tailor phase outcomes and, by extension, sensor properties. For instance, in GLAD-deposited SnO_2_ nanostructures, Singh et al. [160] reported that smaller crystallite sizes and higher crystallinity contribute to higher sensing performance by providing more active sites for CO adsorption. Similarly, Oros et al. [161] fabricated vertical SnO_2_ nanorods using GLAD and attributed enhanced selectivity to increased crystallinity. Additionally, Hwang et al. [162] investigated GLAD-deposited TiO_2_ single-crystal nanohelices and linked their near single crystallinity to improved electron mobility, resulting in faster response times and greater long-term reliability.

In summary, the annealing temperature and duration must be carefully optimized to avoid compromising the structural integrity of GLAD nanostructures. Excessive thermal treatment may lead to densification, collapse of nanocolumns, or loss of porosity [32], thereby reducing the surface area and degrading gas accessibility.

#### 2.3.5. Surface Reactivity Engineering

While material selection and morphology significantly influence gas sensing performance, surface reactivity is equally crucial in determining how the sensor interacts with target gases. Modifying the surface of GLAD-fabricated nanostructures through chemical, physical, or structural strategies enables enhanced adsorption, improved charge transfer, and better selectivity (see Table 3). These strategies fall into three main categories: (1) composition engineering, (2) nanoparticle decoration, and (3) surface functionalization.

Composition engineering involves modifying the material structure or chemistry to enhance gas sensing performance. A key strategy is forming heterostructures such as p–n, n–n, or p–p junctions, which improve charge separation and modulate band structures, thereby boosting sensitivity and selectivity [163,164,165]. For example, a ZnO/CuO p–n heterojunction enhances H_2_S sensing by facilitating efficient charge carrier dynamics [163]. Another effective approach is constructing core–shell architectures, where a NW or nanoparticle core is coated with a shell of another material, often a metal oxide [163,164]. When the shell thickness is tuned to match the Debye length, the entire shell actively participates in gas adsorption and charge modulation. A notable example is the SnO_2_/NiO sensor, which demonstrated exceptional performance when the NiO shell was ~4.2 nm (close to its Debye length,) enabling full shell depletion and heightened sensitivity [166]. Doping is another widely used strategy to tune the electrical and chemical properties of sensing materials [163,164,165]. It introduces new electronic states, modulates carrier concentrations, and increases oxygen vacancy density, enhancing gas adsorption and electron transport. Bhuvaneshwari and Gopalakrishnan systematically studied Fe doping in CuO nanostructures for NH_3_ sensing and observed a 14-fold performance boost with 8 at.% Fe doping at 600 ppm NH_3_ [167]. This enhancement is attributed to the formation of CuO/α-Fe_2_O_3_ heterojunctions, increased hole carrier concentration, expanded surface area, and a favorable flake-on-rod morphology that promotes gas diffusion.

These composition engineering strategies integrate seamlessly with the GLAD technique. As shown in Figure 3G, GLAD enables fabrication of multi-segmented or layered heterostructures by sequentially changing deposition materials during NR growth, forming vertical or tilted p–n and n–n junctions depending on material pairing. Through co-deposition, GLAD can also produce advanced heterostructures like Janus NRs (Figure 3K), checkerboard NRs (Figure 3L), and twisted “candy cane” NRs (Figure 3M), offering added design flexibility. Side-coated or sandwiched NRs (Figure 3H,I) can be engineered by adjusting deposition angles or rotating the substrate mid-growth, enabling spatial control over heterostructure formation and directional gas response. Core–shell NRs (Figure 3J) can be fabricated using conformal GLAD or via post-deposition techniques such as ALD for uniform shell coating on high-aspect-ratio structures. For doping, GLAD supports simultaneous co-deposition (Figure 3O), where a dopant is introduced along with the host material. By precisely tuning deposition rates and substrate rotation speed, the dopant concentration and spatial distribution along the NR length can be finely controlled. This capability allows for gradient or zoned functionalities in next-generation gas sensors, offering enhanced sensitivity and selectivity through engineered material composition.

Surface decoration enhances sensor performance by modifying the outer layer of the sensing material with functional nanomaterials that act as catalysts, sensitizers, or charge-transfer mediators. A common approach is decorating the surface with noble metal NPs such as Pt, Pd, Au, Ir, or Ru [163,164,165]. These NPs influence the electronic structure at the metal–semiconductor interface by forming Schottky barriers, enhancing electronic sensitization, or by introducing plasmonic effects that improve light–matter interactions. Additionally, noble metals catalyze gas reactions through the spillover effect, where adsorbed reactive species migrate from the metal to the oxide surface, further increasing sensitivity. For example, Ir- and Ru-decorated ZnO films, fabricated via ALD, demonstrated enhanced ethanol and SO_2_ sensing, along with improved corrosion resistance and long-term stability [168]. MOS NP decoration is another effective strategy [163,165]. These decorations can form local p–n junctions at the interface with the base material, enhancing charge modulation and sensor response. They can also modify the acid–base properties of the surface, which significantly improves gas selectivity. In a notable example, Van Hieu et al. [169] achieved a nearly 351-fold enhancement in H_2_S detection by decorating SnO_2_ NWs with NiO nanoparticles. The NiO–SnO_2_ heterostructure created multiple n–p–n–p junctions that collapsed upon H_2_S exposure, producing a dramatic resistance drop. At 300 °C, the decorated sensor showed a response of 1372 to 10 ppm H_2_S, compared to just 3.9 for bare SnO_2_. Quantum dots (QDs) can also serve as powerful surface modifiers. By extending the absorption spectrum and enabling visible-light-driven photocatalysis, QDs enhance photoexcited charge separation and surface reactivity. For instance, CdSe QD-decorated ZnO films demonstrated improved room-temperature NO_2_ sensing under 535 nm illumination, with a 150% resistance increase in response to 1.6 ppm NO_2_, attributed to enhanced QD-ZnO contact and charge transfer [170].

GLAD is particularly well-suited for integrating nanoparticles into NRs with high precision and design flexibility. During NR growth, co-deposition enables the incorporation of catalytic materials such as Pt, Au, or Pd directly into the oxide NR matrix (Figure 3N), forming embedded heterojunctions that enhance sensing. Alternatively, post-deposition techniques, including sputtering or side-coating GLAD (Figure 3H–J), can decorate the surfaces of pre-formed NRs with nanoparticles, allowing for tunable distribution and decoration density tailored to specific gas targets. Both MOS NPs and QDs can be integrated using GLAD-based or solution-based methods. Dip-coating or spray-coating from colloidal solutions provides a simple way to achieve uniform or patterned surface decoration [164,165]. Additionally, ligand-assisted decoration uses functionalized molecules to anchor NPs or QDs selectively onto the surface, ensuring stable attachment and further improving sensitivity and selectivity [164,165].

Surface functionalization can enhance gas sensor performance by chemically modifying the sensing surface to improve reactivity and selectivity [165]. A widely used method is silane chemistry, where self-assembled monolayers (SAMs) of organosilanes are formed on oxide surfaces (e.g., SiO_2_), introducing functional groups such as –NH_2_ and –COOH. For noble metals like Au, Ag, or Pt, thiol-based chemistry using alkanethiols achieves similar modification. These functional groups promote selective gas adsorption via specific chemical interactions. For example, Singh et al. [171] functionalized ZnO NWs with APTMS (3-aminopropyltriethoxysilane) and GLYMO (3-glycidyloxypropyltrimethoxysilane), and found that APTMS-functionalized ZnO significantly improved acetone detection, lowering the limit from 6 ppm to 0.5 ppm at 300 °C and increasing the response fivefold over unmodified ZnO. Another powerful strategy involves coating the sensing surface with different MOF materials, which are highly porous, crystalline materials characterized by large surface areas and tunable pore sizes. These act as molecular sieves that enhance gas adsorption and selectivity [172]. Advanced MOF-based designs, including MOF-on-MOF heterostructures and alkyl- or fluorinated-functionalized MOFs, offer further selectivity refinement. As summarized by Peng et al. [165], MOF coatings provide structurally and chemically tunable platforms that enable high-performance and selective gas sensing [172] Similarly, polymer coatings and molecularly imprinted polymers (MIPs) introduce “key–lock” recognition, boosting selectivity and reducing cross-sensitivity, especially valuable for detecting VOCs.

Many of these strategies are well suited for GLAD-fabricated nanostructures. Fan et al. [173] rendered vertically aligned Si NRs hydrophobic by treating them with (heptadecafluoro-1,1,2,2-tetrahydrodecyl)trichlorosilane. Fu et al. [174] designed Au/Si hetero-NRs resembling matchsticks, where the Au tips were selectively functionalized with dithiobis(succinimidyl propionate) (DSP) for anti-Salmonella antibody attachment, while the Si NR surfaces were modified with (3-Aminopropyl)triethoxysilane (APTES) to bind a fluorescent dye (Alexa488-succinimidyl ester). This spatially resolved dual-functionalization enabled simultaneous biorecognition and fluorescence detection, achieving single-cell-level biosensing for *Salmonella*. Table 4 summarizes these surface functionalization techniques, highlighting their advantages and limitations for GLAD-enabled gas sensors.

Although reports of MOF- or polymer-coated GLAD structures remain limited, several integration methods, including solution-based deposition and vapor-phase coating [185], as well as in situ growth [175], have proven effective for applying these functional layers onto complex GLAD geometries. The choice of method depends on the desired coating morphology (e.g., conformal vs. selective), the chemical compatibility with the nanostructured surface, and the targeted sensing function, such as gas selectivity, humidity resistance, or biofouling prevention [175]. 

## 3. GLAD-Enabled Gas Sensing Mechanisms and Device Integration

Gas sensing technologies rely on the detection of changes in specific physical or chemical properties of sensing materials when exposed to target gas molecules. These changes, such as variations in electrical resistance, capacitance, mass, temperature, or optical properties, form the basis for different types of gas sensors, including resistive, capacitive, piezoelectric, optical, and electrochemical sensors. This section focuses on the integration of GLAD-fabricated nanostructured films into various gas sensing platforms, with particular attention to how morphology and material design impact sensitivity, selectivity, response time, and detection limits across different sensing mechanisms.

### 3.1. Resistive Gas Sensors

**Figure 7:**
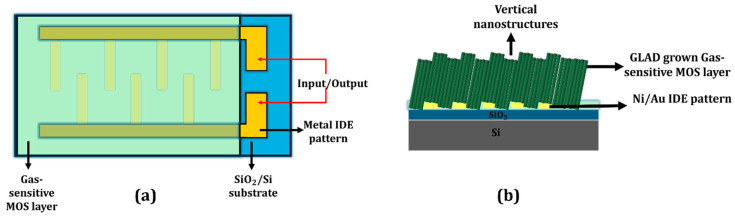
(**a**) Top view of a typical resistive gas sensor, and (**b**) side view of a GLAD-grown vertical nanostructure-based resistive gas sensor.

Resistive sensors are among the most widely studied and practically deployed gas sensors. They function by measuring changes in the electrical resistance of a material upon exposure to reducing or oxidizing gases [186]. This simple operating principle, combined with advantages such as low cost, compact size, low power consumption, long lifetime, and excellent sensitivity, makes them ideal for a range of applications. A typical resistive sensor consists of a MOS film deposited over a pair of electrodes, often with a heater to maintain the optimal operating temperature (Figure 7a). GLAD enables fabrication of vertical nanostructures (Figure 7b) that enhance sensor performance by increasing surface area and defect density. MOS materials, such as SnO_2_, ZnO, and WO_3_, dominate this field due to their excellent semiconducting, thermal, and chemical properties [103,186]. Their ability to adsorb gas molecules, modulated by surface defects and oxygen vacancies, enables efficient charge transfer in response to gas exposure.

The gas sensing mechanism of MOS materials involves surface adsorption and charge transfer processes that alter resistance. At elevated temperatures (typically 150–400 °C), oxygen molecules are adsorbed onto the MOS surface and ionized by capturing electrons from the conduction band. The ionization pathway depends on temperature [16],

(11) $$O_{2}^{gas}+e^{-}\to O_{2\left(ads\right)}^{-}\;\left(T\leq 150\;{}^{\circ}C\right),$$

(12) $$O_{2\left(ads\right)}^{-}+e^{-}\to {2O}_{\left(ads\right)}^{-}\;(150\;{}^{\circ}C<T<300\;{}^{\circ}C),$$

(13) $$O_{\left(ads\right)}^{-}+e^{-}\to O_{\left(ads\right)}^{2-}\;(300\;{}^{\circ}C<T<500\;{}^{\circ}C).$$

These ionized oxygen species withdraw electrons, forming a surface depletion layer in n-type MOS or a hole accumulation layer in p-type MOS, thereby defining the baseline resistance.

When the sensor is exposed to a reducing gas (e.g., H_2_, CO, CH_4_, NH_3_), the gas reacts with adsorbed oxygen species, releasing electrons back into the MOS conduction band. This reduces the depletion layer and thus lowers resistance in n-type materials; in p-type materials, it reduces hole density, increasing resistance. In contrast, oxidizing gases (e.g., NO_2_, O_3_, Cl_2_) extract more electrons from the MOS, widening the depletion layer in n-type and increasing resistance, while in p-type materials, they increase hole concentration, lowering resistance. This reversible change underlies the resistive sensing mechanism. The sensor response $R_{o}$ is quantified as [187],

(14) $$R_{o}=\frac{R_{gas}^{o}}{R_{air}},$$

for oxidizing gases. For reducing gases, $R_{r}$ can be defined as [188],

(15) $$R_{r}=\frac{R_{air}}{R_{gas}^{r}},$$

or [115]

(16) $$R_{r}=\frac{R_{air}-R_{gas}^{r}}{R_{gas}^{r}},$$

where $R_{gas}^{o}$ and $R_{gas}^{r}$ represent the resistance in the presence of the oxidizing/reducing gas, and $R_{air}$ is the resistance under ambient air. The sensitivity *S* of the sensor is the rate of change in response with gas concentration $C_{g}$ [118],

(17) $$S=\frac{dR}{dC_{g}}.$$

A steeper slope indicates higher sensitivity, meaning small concentration changes result in significant resistance shifts. Sensor performance is influenced by temperature, nanostructure morphology, surface area, defect density, and environmental conditions (e.g., humidity). Material parameters such as band gap, Fermi level, crystallite size, and network connectivity also play critical roles in determining sensitivity [140].

The limit of detection (LoD) is defined as the lowest gas concentration that causes a measurable resistance change exceeding three times the baseline noise. A lower (i.e., improved) LoD can be achieved by increasing surface area and defect density, both of which are enhanced by nanostructures [189]. Response and recovery times, representing the duration to reach 90% of the resistance change upon gas exposure and removal, are governed by adsorption/desorption kinetics, which are influenced by material morphology, operating temperature, gas concentration, and external conditions [190]. 

Because gas sensing is fundamentally a surface-driven process, the morphology of semiconductor nanostructures is key to achieving high sensitivity, particularly at low analyte concentrations. GLAD is especially well-suited for constructing high-performance MOS sensing layers due to its precise control over geometry, high density of active sites, tunable pore structures, and rich defect profiles, all of which contribute to improved gas response. Table 5 summarizes reported GLAD-based resistive gas sensors, categorized into pure MOS sensors and those decorated with metal nanoparticles. In the following sections, we further explore the performance of various GLAD-synthesized MOS nanostructures in practical gas sensing applications.

#### 3.1.1. Pure MOS-Based Gas Sensors

**Figure 8:**
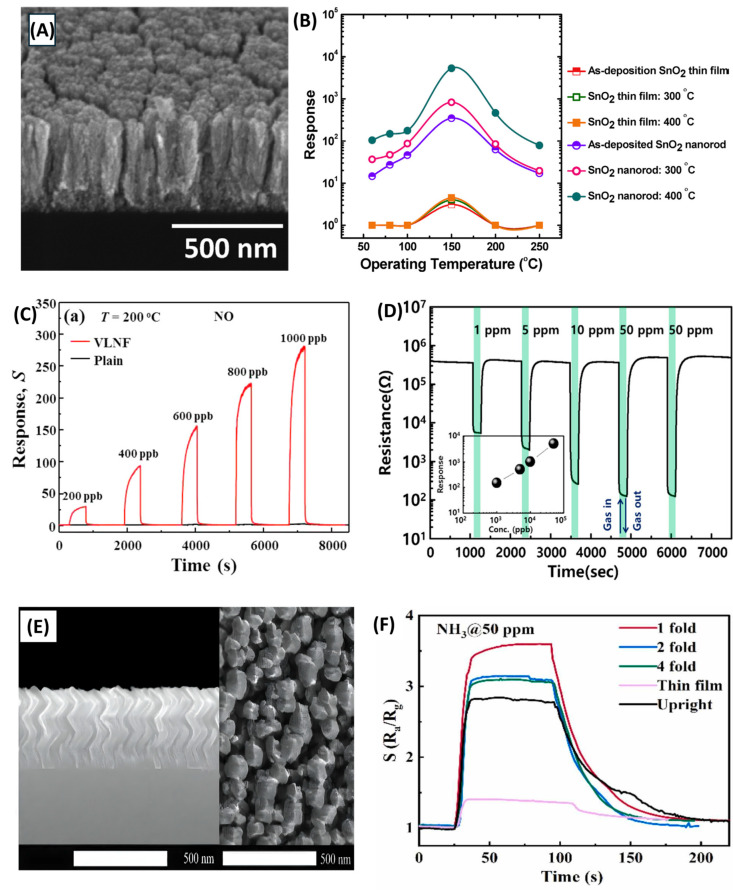
(**A**) A representative cross-section SEM image of $SnO_{2}$ NRs annealed at 400 °C. (**B**) Temperature-dependent response curves at 5 ppm $NO_{2}$ for GLAD deposited and annealed $SnO_{2}$ NRs. Reproduced from [161] with permission from Elsevier. (**C**) The dynamic NO sensing responses of the VLNF WO_3_ sensor compared to a dense, planar WO_3_ thin film sensor under identical conditions (at 200 °C in 80% RH). Reproduced from [194] with permission from the American Chemical Society. (**D**) The ethanol sensing performance of GLAD In_2_O_3_ nanocolumnar films. Reproduced from [73] with permission form Elsevier. Inset shows the log-log plot of the response and ethanol concentration. (**E**) An SEM image of a 4-fold zig-zag TiO_2_ NR array [188]. (**F**) Responses of TiO_2_ nanostructures with different morphologies at 50 ppm NH_3_. Reproduced from [188] with permission from Elsevier.

${SnO}_{2}$**.** Tin oxide (SnO_2_) is one of the most widely used MOSs in gas sensing due to its wide band gap, high stability, and strong gas sensitivity [103,201]. Enhancing its performance typically involves the use of nanostructures to increase surface area and porosity [202]. GLAD, particularly when combined with PVD methods like sputtering or e-beam evaporation, enables precise control over SnO_2_ morphology. Chundak et al. [126] demonstrated this tunability by fabricating various SnO_2_ architectures, slanted pillars, zigzags, spirals, bush-like forms, and vertical posts, by varying the deposition angle and substrate rotation. Their analysis showed that slanted pillars had the highest surface area, establishing a direct link between morphology, surface roughness, and gas-sensing potential. GLAD-fabricated SnO_2_ sensors exhibit high sensitivity, low LoD, and fast response/recovery for gases like NO_2_, CO, C_2_H_2_, and VOCs. Oros et al. employed GLAD sputtering to produce vertically aligned SnO_2_ NRs and reported an impressive NO_2_ response of 5310 to 5 ppm at 150 °C, with a LoD of 6 ppb and response/recovery times of 5.9 and 2.6 min, respectively [161]. As shown in their results, although the NR morphology (Figure 8A), characterized by rod shape, porosity, and vertical alignment, remained largely unchanged with annealing, its crystallinity improved significantly, especially at 400 °C. This enhancement in crystallinity led to substantially better gas-sensing performance. At 5 ppm NO_2_, the 400 –annealed NRs outperformed dense films by a factor of ~642 (Figure 8B). All NR sensors, regardless of annealing, consistently outperformed dense films due to their higher accessible surface area, better gas penetration, and lower grain boundary resistance. Lee et al. [105] applied e-beam GLAD to fabricate porous SnO_2_ nanocolumns for fire detection, achieving early detection of toxic gases (HCl, CO, VOCs) from PVC decomposition. At 200 °C, their sensor outperformed commercial smoke detectors, and at 350 °C, it reached a response of 294.9% to high gas concentrations. In another study, GLAD-deposited SnO_2_ NRs showed a 6% response to 10 ppm C_2_H_2_ with a 1 ppm LoD at 350 °C, addressing critical transformer safety concerns [116]. Operating at 350 °C, the as-deposited sensors showed a 6% response to 10 ppm C_2_H_2_ with a detection limit of 1 ppm. Singh et al. [160] investigated CO sensing using GLAD-assisted RF sputtered SnO_2_. By optimizing the deposition angle (θ=50°), they achieved a remarkable 150% response to 500 ppm CO at 110 °C, far surpassing baseline films (15% at θ=0°). Performance was optimized at moderate angles due to enhanced porosity and surface roughness, with diminishing returns at higher angles due to reduced crystallinity. Sensor response increased linearly with CO concentration and remained selective against NH_3_, CH_4_, CO_2_, and ethanol. Mohajir et al. [143] further explored how GLAD angle and annealing affect SnO_2_ porosity and sensing performance. Films deposited at θ=80° and annealed at 500 °C showed the highest surface/internal porosity, enabling detection of benzene at 30 ppb at 400 °C. Structural tuning also enabled selectivity: using four GLAD-fabricated SnO_2_ sensors, each with distinct porosity profiles, they distinguished between BTEX compounds (benzene, toluene, ethylbenzene, xylene) via linear discriminant analysis (LDA). This created a virtual sensor array using a single material platform, demonstrating a powerful strategy for selective VOC sensing through microstructural engineering.

${WO}_{3}$**.** Tungsten trioxide (WO_3_), an n-type semiconductor, is among the most extensively studied gas-sensing materials due to its high chemical stability [103], strong surface catalytic activity, and sensitivity to a wide range of toxic gases, including ${NH}_{3}$ [203], $H_{2}S$ [204], $H_{2}$ [205], and ${NO}_{2}$ [206]. Nanostructured WO_3_ exhibits enhanced sensing performance due to its high surface-to-volume ratio and active surface sites [202]. GLAD techniques enable fabrication of diverse WO_3_ morphologies (e.g., slanted NRs, zigzags, spirals, and villi-like nanofingers [207,208,209,210,211,212,213,214]), with gas sensing being among the most intensively explored applications. GLAD-fabricated WO_3_ structures have shown excellent sensitivity, selectivity, and low detection limits toward gases such as NO_2_, ethanol, ozone, dodecane, acetone, and nitric oxide [35,192]. These properties are highly tunable via deposition angle, rotation speed, and post-annealing treatments. Horprathum et al. [187] fabricated vertically aligned WO_3_ NRs via DC magnetron sputtering at θ=85°, followed by annealing at 400–500 °C. The resulting crystallization increased porosity and reduced grain boundary resistance, resulting in a high NO_2_ response at 250 °C and a LoD of 0.1 ppm. Ahmad et al. [191] studied film thickness effects on WO_3_ NRs and found a thickness-dependent sensitivity: thinner films favored ethanol detection, while thicker films enhanced NO_2_ response (max = 1075 at 150 °C, 10 ppm NO_2_) due to depletion layer effects. Xu et al. [192] used GLAD with a reactive gas pulsing process to fabricate inclined and zigzag WO_3_ columns. The zigzag structure showed a 7.5% response to 50 ppm dodecane, while the inclined configuration achieved an 8.1% response to 300 ppb ozone. These enhancements were primarily attributed to increased porosity, which facilitated more efficient gas diffusion and adsorption. Zarzycki et al. [195] found that intermediate deposition angles (θ=75°) yielded the highest response (~1.5) to acetone under 50% RH, attributed to enhanced columnar porosity and roughness. Low (0°, 45°) and high deposition angles (85°) produced flatter, less responsive films. Bronicki et al. [35] demonstrated that increasing substrate rotation speed (up to 20 rpm) enhanced WO_3_ nanostructure alignment and porosity, significantly improving acetone sensing at 300 °C and 55% RH. Moon et al. [194,215] developed villi-like nanofinger (VLNF) WO_3_ films via GLAD for exhaled NO detection. Figure 8C presents the dynamic NO sensing responses of the VLNF WO_3_ sensor in comparison to a dense planar WO_3_ thin film sensor, both tested at 200 °C under 80% RH. The VLNF sensor exhibits a clear and rapid resistance increase upon exposure to NO concentrations ranging from 200 to 1000 ppb. Notably, the response of the VLNF sensor at 1000 ppb NO is nearly 200 times greater than that of the planar sensor, and even at 200 ppb NO, the VLNF sensor shows a distinct and repeatable signal after 5 consecutive pulses of NO. After annealing, these structures enabled ppt-level NO detection (LoD = 206 ppt) at 168 °C under 80% RH, with strong selectivity toward H_2_S and NH_3_. The high performance was attributed to porous column geometry, the presence of double Schottky barriers at grain junctions, and the high surface area, all of which contribute to enhanced gas-solid interactions. These features make the sensors highly suitable for breath diagnostics and integration into electronic nose (e-nose) platforms.

${In}_{2}O_{3}$**.** Indium oxide (In_2_O_3_) is a well-established n-type metal oxide semiconductor extensively explored for gas sensing due to its wide band gap, high concentration of oxygen vacancies, and excellent chemical and thermal stability [103,202]. Despite its popularity, studies specifically using GLAD to fabricate pure In_2_O_3_ gas sensors remain relatively limited. Han et al. [73] optimized In_2_O_3_ nanocolumnar films via GLAD with e-beam evaporation to enhance gas sensing. By adjusting the deposition angle (80–85°), rate, and vacuum level, they tuned nanocolumn size, porosity, and oxygen vacancy concentration, resulting in baseline resistances from 10^2^ to 10^5^ Ω. This enabled selective sensitivity: the best film for NO_2_ (oxidizing gas) showed a response of 176 ($R_{g}/R_{a}$) at 5 ppm and a LoD as low as 2 ppt; while for ethanol (reducing gas), a low-rate film achieved a response of 929 ($R_{a}/R_{g}$) at 50 ppm with a LoD of 370 ppt. Figure 8D shows the ethanol sensing performance, characterized by a strong, repeatable, and linear response in log scale (inset). All sensors exhibited rapid response/recovery (20–90 s), driven by high porosity, gas diffusion, and intergrain barrier modulation. Complementing this, Song et al. [196] examined the influence of sensor miniaturization and electrode design on the performance of GLAD-deposited In_2_O_3_ nanocolumns. The study employed Pt interdigitated electrodes (IDEs) with sensing areas ranging from 1 mm^2^ down to 0.01 mm^2^ and explored the effects of different GLAD angles (78°, 82°, and 85°). Their findings showed that films fabricated at 85° formed highly porous, vertically aligned nanocolumns with excellent crystallinity, as confirmed by SEM and XRD analyses. Among all configurations, a sensor with a 0.3 mm × 0.3 mm sensing area exhibited the best performance, offering rapid response (~10 s) and high selectivity toward various VOCs, including ethanol, acetone, benzene, toluene, and formaldehyde, at 300 °C. The study used the double Schottky barrier model to explain the sensing mechanism, in which intergrain resistance was dominant in porous nanocolumn films, in contrast to the interface resistance in dense films.

ZnO**.** Zinc oxide (ZnO) is an n-type MOS widely recognized for its excellent thermal and chemical stability, high exciton binding energy, and notable gas selectivity, making it a strong candidate for gas sensing applications [103,202]. ZnO nanostructures fabricated using GLAD have demonstrated excellent gas sensing capabilities. Singh et al. [117] fabricated porous ZnO columnar films via GLAD sputtering at varying deposition angles and identified 70° as the optimal angle, producing vertically aligned nanostructures with circular heads, high crystallinity, and enhanced surface roughness. The resulting CO sensor achieved a high response of 575 ($R_{a}/R_{g}-1$) toward 500 ppm at 150 °C, along with fast response (28 s) and recovery (97 s) times. Luo et al. [198] developed nanospiral ZnO films using GLAD sputtering for NO detection. These sensors outperformed dense ZnO films, delivering a response of 16.9 towards 100 ppb NO at 150 °C under 40% RH, with a LoD as low as 10 ppb. The optimized sensor also showed good selectivity against NH_3_, CH_4_, H_2_, and CO, stable operation over 20 days, and a strong response (~14) under high humidity (80% RH) at 90 ppb NO. Aier and Dhar [197] fabricated catalyst-free ZnO NRs (~500 nm long, 100 nm wide) on IDEs using GLAD magnetron sputtering and tested them for SO_2_ sensing. At 300 °C, the sensors responded with 18.19% to 3 ppm SO_2_, with a response time of 41.8 s and a recovery time of 84.9 s. They also showed high selectivity, responding only minimally to NO_2_ (2.75%) and CO (1.45%), demonstrating the effectiveness of GLAD-fabricated ZnO nanostructures for selective and sensitive gas detection.

${TiO}_{2}$**.** Titanium dioxide (TiO_2_), another n-type semiconductor, is widely studied for gas sensing due to its chemical stability, non-toxicity, excellent electron transport, and photoelectric properties [151,216,217]. Among its polymorphs, anatase is preferred in gas sensors for its high oxygen vacancy density and strong surface reactivity [217]. Though TiO_2_ has been extensively used in gas sensing, GLAD-specific studies remain limited. Hwang et al. [162] developed high-performance H_2_ sensors using near-single-crystalline TiO_2_ nanohelices grown by GLAD and annealed at 500 °C to form anatase. With diameters <30 nm (close to the Debye length) and a top–bottom electrode configuration, the sensors showed ~10× improved response (15.6 at 50 ppm), a LoD of 1.37 ppm, and a response time <10 s, far outperforming conventional TiO_2_ films. Jyothilal et al. [218] fabricated humidity sensors using GLAD-deposited tilted TiO_2_ NRs at θ=80°, followed by annealing at 500 °C. The highly porous structure achieved a fast response (145 ms) and recovery (210 ms) at 95% RH, with a sensitivity of 8.3 × 10^3^, outperforming previously reported TiO_2_-based humidity sensors. The sensor remained stable for more than 70 days and successfully monitored post-exercise breath dehydration. Dai et al. [188] studied various GLAD-fabricated TiO_2_ morphologies, including nanofilms, upright rods, and zigzag structures (see an example in Figure 8E), for room-temperature NH_3_ sensing. Figure 8F compares the NH_3_ sensing responses at 50 ppm for TiO_2_ nanostructures with different morphologies. Among them, the 1-fold zigzag NRs showed the highest performance, with a response of 3.6 to 50 ppm NH_3_, a LoD of 200 ppb, and a response time of ~4.5 s. Molecular dynamics simulations confirmed spontaneous NH_3_ adsorption, with the 1-fold zigzag offering the most favorable adsorption energy. The sensor also demonstrated long-term stability (45 days) and operated optimally at 25 °C and 60% RH.

CuO**.** As a p-type semiconductor, CuO offers several properties that make it attractive for gas sensor development [219,220,221]. CuO exhibits strong interactions with both oxidizing and reducing gases. These properties, along with its high surface reactivity, make it a suitable candidate for detecting a variety of toxic and combustible gases, including acetone, ethanol, H_2_, CO, H_2_S, NH_3_, and NO_2_. Rydosz et al. [129] investigated the use of GLAD magnetron sputtering to fabricate ultrathin CuO films (4–15 nm) for sub-ppm acetone detection, targeting noninvasive diabetes monitoring via breath analysis. These films were deposited at a deposition angle of θ=85°, enabling the formation of tilted, porous nanostructures with an enhanced surface-to-volume ratio. Among the films tested, the 8 nm-thick CuO layer deposited in fully reactive conditions (100% O_2_) showed the highest sensitivity, achieving a LoD of 0.25 ppm at 350 °C within the acetone concentration range found in healthy human breath. The results demonstrated that precise control over film thickness and GLAD geometry can significantly improve gas sensing performance. However, sensor response decreased markedly under high humidity (80% RH), indicating the need for strategies to mitigate moisture interference in practical applications.

${Cu}_{2}O$**.** Cuprous oxide (Cu_2_O), though less extensively studied than popular n-type semiconductors like SnO_2_, ZnO, and WO_3_, is emerging as a promising material for gas sensing applications [222]. As a p-type semiconductor, Cu_2_O provides complementary sensing characteristics, making it particularly effective for detecting oxidizing gases such as NO_2_, as well as certain reducing gases like H_2_S, NH_3_, and CO. Ben Nacer et al. [130] investigated the ethanol gas sensing performance of nano-columnar Cu_2_O thin films synthesized by GLAD thermal evaporation. Copper films were deposited on glass substrates at various vapor incident angles (0°, 60°, 75°, and 85°), followed by thermal oxidation at 250 °C to form pure cubic-phase Cu_2_O. The sensor fabricated at θ=85° exhibited the best response, showing a sensitivity of 8.12 ($R_{g}/R_{a}$) to 500 ppm ethanol at an optimal operating temperature of 200 °C. This enhanced performance was attributed to higher porosity (~37%), increased surface area, and finer grain structures that promote gas adsorption. The LoD was estimated at 30 ppm, and the sensor demonstrated good long-term stability with less than 3% signal drift over one month.

**Vanadium oxide.** Vanadium oxide, particularly vanadium dioxide (VO_2_) and vanadium pentoxide (V_2_O_5_), has emerged as a promising material for gas sensor applications due to its distinct physical and electronic properties. VO_2_, a notable p-type semiconductor, that undergoes a metal-insulator transition (MIT) near ~68 °C, changing from a low-temperature monoclinic insulating phase to a high-temperature rutile metallic phase. VO_2_ is especially suited for detecting reducing gases such as CH_4_, isopropanol (C_3_H_8_O), butanol (C_4_H_9_OH), and NO_2_, with good selectivity and a fast response near the MIT temperature [223,224,225]. However, its application can be limited by thermal instability, hysteresis effects, and difficulty in controlling stoichiometry, making it more complex to implement in practical sensors. In contrast, V_2_O_5_, an n-type semiconductor with a wide bandgap (~2.2–2.7 eV), offers excellent chemical stability and strong surface reactivity. Among its polymorphs, the orthorhombic α-V_2_O_5_ phase is the most stable and commonly employed in sensing. V_2_O_5_ has been widely used for the detection of oxidizing gases such as NO_2_, benzene, NH_3_, and other VOCs [226,227,228].

In 2013, Ciprés [229] explored the fabrication and characterization of VO_2_-based thin films using the GLAD DC magnetron sputtering. The main goal of the work was to study how different GLAD-induced nanostructures, such as inclined columns, zigzags, and spirals, affected the MIT properties and gas sensing performance of the films. After deposition, films were annealed to form various vanadium oxide phases (mainly VO_2_). One selected film, prepared at θ=85° with specific oxygen pulse parameters and annealed at 550 °C, was tested for ozone sensing, demonstrating an optimal operating temperature of ~370 °C and repeatable changes in resistivity upon gas exposure. In a recent 2024 study, Sanchez et al. [131] developed nano-sculptured V_2_O_5_ thin films using DC magnetron sputtering with conventional (θ=0°) and GLAD (θ=80°) techniques for benzene gas sensing. After post-deposition annealing at 500 °C, GLAD-fabricated films showed highly porous structures and crystallized into the α-V_2_O_5_ orthorhombic phase with improved chemisorbed oxygen content. Compared to conventionally deposited films, the GLAD-based sensors exhibited significantly enhanced sensitivity and repeatability, with a limit of detection as low as 28 ppb under dry conditions and 36 ppb under 60% RH. The improved sensing performance was attributed to the highly porous, anisotropic nanostructures created via GLAD, which promoted better gas diffusion and increased interaction with benzene molecules. This work highlights the potential of GLAD-deposited V_2_O_5_ as a reliable and miniaturized gas sensor for trace-level benzene detection in real-world environmental conditions.

#### 3.1.2. Mixed Oxide and Multilayer MOS NR Array

**Indium tin oxide.** Indium tin oxide (ITO) has emerged as a promising alternative to traditional MOS like In_2_O_3_ and SnO_2_ for gas sensing applications, although it has been less extensively studied [230,231]. Notably, Yao et al. [232] conducted a comprehensive investigation into the fabrication and gas sensing performance of ITO NRs for NO_2_ gas sensing using GLAD. Their results showed that ITO NRs grown at high incident angles (up to 86°) displayed significantly enhanced NO_2_ sensing capabilities compared to conventional thin-film ITO, owing to their high surface-to-volume ratio and increased porosity. The study compared three sensor architectures: a flat ITO film, a single-layer ITO NR array, and a double-layer NR array constructed using Fe_2_O_3_ or SiO_2_ as seed layers. Among these, the double-layer configuration achieved superior sensitivity by increasing NR spacing and reducing the number of conductive paths, thereby amplifying resistance changes during NO_2_ exposure. This configuration demonstrated a LoD as low as 50 ppb and a response time of around 20 min. Additionally, by varying the deposition angle between 60° and 86°, the authors observed that higher angles led to increased porosity and NR tilt, decreased interconnectivity, and ultimately enhanced gas response.

**Multilayer MOS NR array.** MOS/MOS heterojunction structures have emerged as a powerful strategy to enhance gas sensor performance [17,120]. By combining two different MOSs, whether p–n, n–n, or p–p types, these heterostructures benefit from band alignment and interface-induced charge modulation. This leads to enhanced electron depletion or hole accumulation at the junction, which amplifies the change in sensor resistance upon gas exposure. The built-in electric field at the heterojunction interface improves charge separation and facilitates more efficient gas–solid interactions. Numerous studies have demonstrated that heterojunctions, such as CuO/SnO_2_, WO_3_/TiO_2_, ZnO/SnO_2_, and In_2_O_3_/SnO_2_, significantly improve sensitivity, selectivity, and response/recovery times compared to single-oxide sensors [233]. This enhancement is attributed to synergistic effects between the two oxides, including improved surface reactivity, tailored band structures, and extended active adsorption sites. Bikesh et al. [199] reported a SnO_2_/TiO_2_ heterojunction NR structure-based GLAD sensor for ethanol sensing. The fabrication process involved sequential deposition: first, a SnO_2_ seed layer (~50 nm), followed by SnO_2_ NRs (~250 nm) at θ=85°, and then a TiO_2_ NR layer (~250 nm) directly on top. The final device was equipped with interdigitated Ag electrodes for sensing measurements. The sensor exhibited good ethanol sensing performance at an operating temperature of 150 °C. At 200 ppm ethanol, the sensor demonstrated a response of ~3.2, with corresponding response and recovery times of ~41 s and ~84 s, respectively. It also showed a higher sensitivity to ethanol compared to acetone, attributed to the favorable interaction between ethanol and the (110) planes of SnO_2_. The sensor response in terms of current increased significantly with ethanol concentration (from 0.1 mA in air to ~1.8 mA at 250 ppm ethanol under a 3 V bias). The improved gas sensing performance was attributed to the synergistic effects of the SnO_2_/TiO_2_ heterojunction and the vertically aligned porous NW morphology. The heterojunction enhanced charge carrier separation and increased the density of reactive oxygen species due to band alignment (SnO_2_ has a higher work function than TiO_2_), facilitating better charge transfer and gas interaction. The GLAD-based vertical NW structure provided a high surface-to-volume ratio and ample active sites for gas adsorption and desorption, further improving response and recovery times.

#### 3.1.3. Metal Doped or Decorated MOS for Gas Sensing

The integration of metal dopants or metal NPs into MOS nanostructures is a widely used strategy to enhance gas sensing performance. These enhancements arise primarily from four interconnected mechanisms: electronic sensitization, chemical sensitization, catalytic effects, and structural & surface engineering effects. Together, these mechanisms tailor the physicochemical interactions between the MOS surface and target gas molecules, leading to improved sensor response, selectivity, and operating efficiency [234].

Electronic sensitization refers to the modification of the electronic structure of the sensing material through doping or junction formation [235]. When a metal dopant is incorporated into the MOS lattice (e.g., substituting host cations), it alters the charge carrier concentration, bandgap, and Fermi level of the material [122]. This adjustment can either increase electron density (n-type doping) or hole density (p-type doping), thereby enhancing the baseline conductivity and amplifying the resistance change upon gas exposure. For example, Sb^5+^ doping in SnO_2_ increases free electron concentration, which improves conductivity and sensitivity to reducing gases such as CO or H_2_ [103]. Similarly, the formation of Schottky junctions between metal NPs (e.g., Pt, Pd, Au) and MOS materials creates energy barriers that modulate carrier transport [120,121]. These barriers are sensitive to surface reactions and gas adsorption, amplifying resistance changes in response to gas exposure.

Chemical sensitization involves the catalytic interaction of metal NPs with adsorbed gas species, which promotes surface activation and spillover effects. In this process, reactive gas molecules are dissociated on the surface of metal NPs into intermediate species (e.g., H atoms, O^−^), which then spillover to the MOS surface, where they participate in electron exchange reactions [120,121]. This spillover accelerates the redox interactions between the sensing layer and target gases, effectively lowering the activation energy for surface reactions. For instance, Pd or Pt NPs facilitate H_2_ dissociation and promote enhanced H_2_ sensing at lower temperatures [123].

The catalytic effect stems from the inherent chemical reactivity of noble or transition metal dopants/NPs. These metals act as catalysts by lowering the activation barrier for gas adsorption and reaction, especially in oxidizing/reducing environments [121,123]. This capability enables sensing at lower operating temperatures, which is critical for portable or wearable sensors. Furthermore, the catalytic oxidation of reducing gases (e.g., ethanol, CO, NH_3_) on the NP surface can lead to localized heat or electron release, further modulating the conductivity of the underlying MOS.

In addition to electronic and chemical effects, doping and decoration often induce morphological changes, such as increased surface roughness, porosity, and crystallinity [236]. These changes enhance gas diffusion and adsorption and increase the density of active sites, further amplifying sensor performance. Especially in nanostructured platforms (e.g., NRs, NWs, nanospirals), dopants can stabilize high-surface-area architectures and reduce grain boundaries, minimizing electron scattering and improving the signal-to-noise ratio.

Metal doping and metal NP decoration have been consistently used as highly effective strategies for enhancing the gas sensing performance of GLAD-fabricated MOS nanostructures.

**Figure 9:**
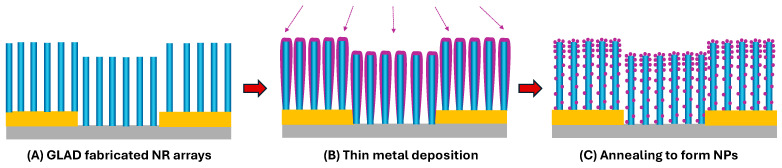
The general procedure used in the literature to form metal NPs decorated GLAD MOS nanostructures: (**A**) MOS NR array fabricated by GLAD; (**B**) ultra-thin layer metal deposition; and (**C**) annealing for metal NP formation.

Metal NP decoration on GLAD-fabricated MOS nanostructures is typically achieved through a two-step process (see Figure 9 [113,114,115,116,117,118,119,200,237,238]): (1) fabrication of highly aligned MOS NRs or nanocolumn arrays using GLAD, and (2) deposition of a thin metallic layer to form discrete NPs on the nanostructure surfaces using either DC or RF magnetron sputtering, or electron beam evaporation. Metal thicknesses are carefully controlled, typically in the range of 1–5 nm, to ensure discontinuous NP formation. Upon post-deposition annealing, these ultrathin metal films can spontaneously agglomerate into well-distributed NPs due to surface diffusion, driven by minimization of surface energy. The size and distribution of NPs can be tuned by adjusting the metal thickness, annealing temperature, and duration. Alternatively, multilayer GLAD-metal deposition cycles can be used to create sandwich-like architectures with alternating layers of MOS and metal to achieve full-surface and interfacial decoration [112].

**Figure 10:**
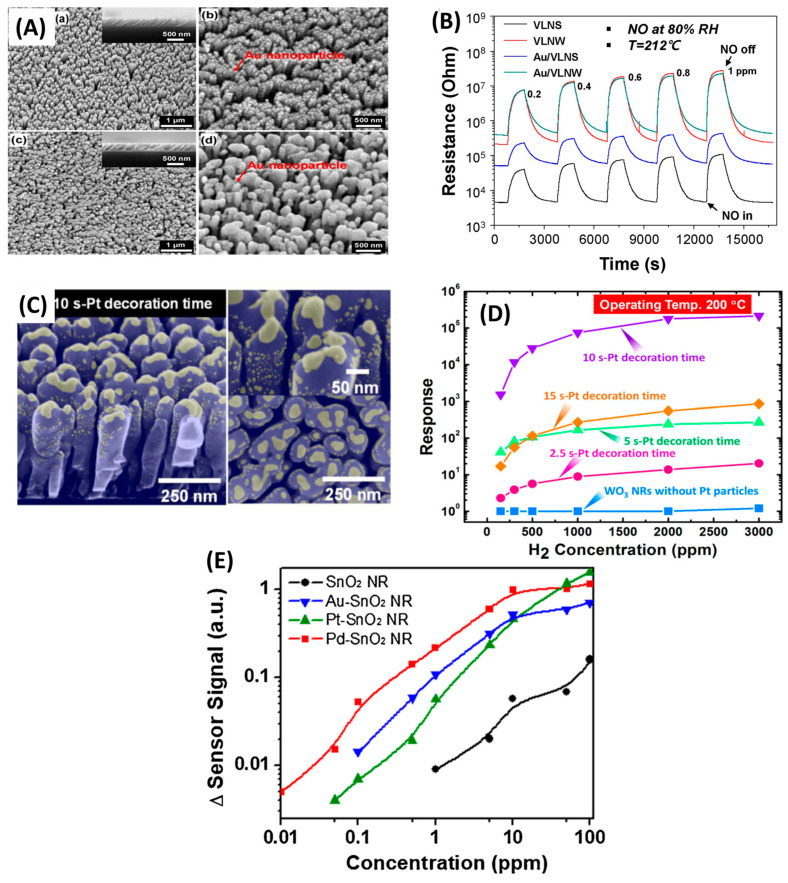
(**A**) (**a**,**b**) Top-view and cross-sectional FE-SEM images (inset) of VLNs of SnO_2_ (VLNS) and Au-functionalized VLNS (Au/VLNS). (**c**,**d**) Top-view and cross-sectional FE-SEM images (inset) of VLNs of WO_3_ (VLNW) and Au-functionalized VLNW (Au/VLNW) (**B**) Response curve of each channel in the CEN as a function of NO in 80% Rh at 212 °C. Reproduced from [238] with permission from Elsevier. (**C**) The SEM image of WO_3_ NR film with a Pt decoration time of 10 s, and (**D**) the gas response of WO_3_ NRs with different Pt decoration times toward 100−3000 ppm of H_2_ at the optimal operating temperature of 200 °C. Reproduced from [114] with permission from American Chemical Society. (**E**) The sensor response of the different metal NP coated SnO_2_ NR sensors as a function of C_2_H_2_ concentration in log-log scales. Reproduced from [116] with permission from Elsevier.

**Au NP decorated.** Jeon et al. [112] fabricated vertically aligned SnO_2_ nanobamboo structures using GLAD, incorporating 2 nm-thick Au NP layers between each SnO_2_ segment to achieve full-surface decoration. By repeating this alternating deposition cycle five times, they formed a multilayered sandwich-like architecture approximately 500 nm in height. These Au NP-decorated SnO_2_ nanobamboos exhibited outstanding gas sensing performance. The authors used calibration curves of the sensor response to C_2_H_5_OH, CH_3_COCH_3_, and C_7_H_8_ gases over the 1–5 ppm concentration range to calculate sensitivities (slopes), which were 0.170 ppb^−1^ for ethanol, 0.073 ppb^−1^ for acetone, and 0.030 ppb^−1^ for toluene. These results support the potential of the sensor for detecting trace levels of volatile reducing gases, with theoretical detection limits in the sub-ppb range. Moon et al. [238] fabricated 2 × 2 sensor arrays using VLNs of SnO_2_ and WO_3_, both in pristine form and with Au NP decoration, Figure 10A. The sensors modified with Au NPs showed substantially improved detection of NO (Figure 10B) and NH_3_ gases, achieving detection limits in the low ppb range even under high humidity (80% RH). The inclusion of Au NPs not only increased sensitivity but also reduced humidity interference, making the sensors more reliable in real-world environments. Kang et al. [237] extended this strategy to a multi-gas sensor array comprising nanocolumnar films of SnO_2_, In_2_O_3_, WO_3_, and CuO, both with and without Au NP decoration. A 1 nm Au layer was deposited via electron beam evaporation, forming uniformly dispersed Au NPs on the nanocolumn surfaces. The resulting sensors demonstrated excellent batch uniformity (base resistance variation ~10%) and gas response consistency (<5% variation), enabling accurate detection of gases such as CO, NH_3_, NO_2_, CH_4_, and acetone. Au NP-functionalized sensors achieved rapid response times ranging from 1 to 8 s, depending on the analyte. Across all three studies, the enhanced gas sensing performance of Au NP-decorated MOS nanostructures was primarily attributed to three synergistic effects: the catalytic activity of Au NPs facilitating gas molecule dissociation, the spillover of reactive species like oxygen ions onto the metal oxide surface to increase active sites, and electronic sensitization via Schottky barrier formation at the Au/oxide interface, enhancing charge modulation during gas exposure.

**Pt NPs decorated.** Horprathum et al. [114] demonstrated that WO_3_ nanorods synthesized via GLAD exhibited negligible response to H_2_ gas when left undecorated. However, after decorating the surface with Pt NPs through brief DC sputtering (see Figure 10C), the sensor response dramatically improved. Figure 10D shows the hydrogen sensing response of WO_3_ NRs with different Pt decoration times (0–15 s) as a function of H_2_ concentration (150–3000 ppm) at 200 °C. The response increases dramatically with increasing Pt decoration time, peaking at 10 s with a maximum response of 2.2 × 10^5^ at 3000 ppm H_2_. However, further decoration to 15 s led to a drop in performance due to Pt nanoparticle agglomeration. The optimal sensor (10 s Pt decoration) achieved a LoD of approximately 0.5 ppm, demonstrating exceptional sensitivity. This enhancement was attributed to the catalytic role of Pt in dissociating hydrogen molecules into atomic hydrogen, which subsequently spills over onto the WO_3_ surface, reacts with chemisorbed oxygen species, and releases electrons into the conduction band, thereby reducing the resistance. Liu et al. [113] investigated the NO_2_ sensing performance of WO_3_ NR films decorated with Pt NPs, fabricated using GLAD, followed by post-deposition annealing. The Pt NP-decorated GLAD WO_3_ sensors exhibited significantly enhanced sensitivity, selectivity, and faster response and recovery times compared to both planar WO_3_ films and undecorated GLAD WO_3_ structures. This improvement was attributed to the high surface area and porosity of the NR architecture, the catalytic activity of the Pt nanoparticles, which promoted NO_2_ adsorption via spillover, and the formation of Schottky barriers at the Pt/WO_3_ interface due to work function differences, which increased the electron depletion region and amplified the sensor’s resistance response to NO_2_ exposure.

**Pd NPs decorated.** Kim et al. [115] demonstrated that Pd NP-coated SnO_2_ NR sensors, fabricated using a GLAD-based approach, showed exceptional hydrogen sensing capabilities. These sensors achieved a high response value of ~104 for 1% H_2_ in nitrogen at room temperature, along with a rapid response time of ~15 s and an impressive LoD of approximately 0.2 ppm. When tested in transformer oil, a relevant industrial application, they still performed robustly, with a response of 96 to 480 ppm H_2_ and stable sensing behavior across temperatures ranging from 20–80 °C. The sensors also demonstrated strong selectivity for H_2_ over other gases, such as CO, CO_2_, and C_2_H_2_. In a separate study, Singh et al. [117] compared undecorated ZnO NRs to Pd NP-decorated ZnO NRs for CO detection. The Pd-ZnO sensors displayed a dramatically enhanced response of 1022 (compared to the undecorated counterpart) under the same CO concentration and temperature conditions. Moreover, they exhibited faster response and recovery times of 17 s and 23 s, respectively, and showed high selectivity over other gases, such as NH_3_, NO_2_, CH_4_, liquefied petroleum gas (LPG), and CO_2_. Similarly, Liu et al. [118] investigated Pd NP-decorated WO_3_ nanostructured films for NO_2_ detection. The sensors, optimized to operate at 150 °C, exhibited a sensitivity of 1.85 for 0.5 ppm NO_2_ and a notably high response of 191.35 at 10 ppm. Selectivity tests confirmed minimal interference from gases, such as NH_3_, CO, acetone, and ethanol. These performance metrics represented a substantial improvement over undoped WO_3_ films, which generally require higher operating temperatures and show lower sensitivity. The enhancements observed across these studies were primarily attributed to the synergistic effects introduced by Pd NP decoration. First, Pd can act as a catalytic agent, facilitating the dissociation of target gases (e.g., H_2_ or NO_2_) into reactive atomic species. These species then react with chemisorbed oxygen on the MOS surface, releasing trapped electrons back into the conduction band and significantly altering the electrical resistance. Second, the spillover effect plays a key role, where Pd nanoparticles not only catalyze the adsorption of gas molecules but also promote the migration of reactive intermediates (such as hydrogen atoms or oxygen species) onto the underlying MOS surface, thereby expanding the active sensing area and enhancing reactivity. Third, Schottky barrier formation at the Pd/MOS interface (e.g., Pd/SnO_2_, Pd/ZnO, or Pd/WO_3_) can modulate the electron transport properties of the sensor. Due to the difference in work functions between Pd and the MOS, a depletion region forms at the interface, which becomes more or less conductive upon gas adsorption, amplifying the sensing signal. In some cases, gas exposure (e.g., to H_2_) can lead to the formation of Pd hydride (PdHx), which reduces the work function of Pd and lowers the Schottky barrier height, further enhancing electron mobility and sensor response.

Lee et al. [116] conducted a comparative study on SnO_2_ NR sensors decorated with Au, Pt, and Pd nanoparticles for C_2_H_2_ detection. Figure 10E presents the sensor response (Δ Sensor Signal) of bare and metal-coated SnO_2_ NR sensors, specifically Au, Pt, and Pd NP-coated, as a function of C_2_H_2_ concentration on a logarithmic scale. All sensors exhibit a linear increase in response with increasing gas concentration (0.01–50 ppm), indicating consistent exponential response behavior typical of MOS gas sensors. Among them, Pd NP-coated SnO_2_ NRs show the highest sensitivity, with a strong linear response even at low concentrations. The enhanced sensing capability was primarily attributed to the catalytic and electronic sensitization effects of the noble metals. In particular, Pd significantly promoted the formation of oxygen vacancies and the dissociation of oxygen molecules, increasing the concentration of reactive oxygen ion species on the SnO_2_ surface. This led to a denser population of active sites and a thicker electron depletion layer, amplifying resistance changes upon gas exposure. XPS analysis supported these findings, revealing the highest oxygen vacancy concentration (32.28%) in the Pd NP-decorated SnO_2_, directly correlating with its superior sensing performance.

**Other metal decorations.** Song et al. [119] developed high-performance acetone sensors using Rh-decorated WO_3_ NRs fabricated by GLAD. Vertically aligned WO_3_ NRs were deposited onto Pt interdigitated electrodes at θ=80° via e-beam evaporation, followed by Rh decoration with varying thicknesses (0.5–3 nm) using on-axis deposition. The samples were annealed at 550 °C for 2 h in air to enhance crystallization and form NPs. Gas sensing tests showed superior sensitivity to acetone among various VOCs. At an optimal Rh thickness of 0.5 nm and an operating temperature of 300 °C, the sensor exhibited a theoretical LoD of 131 ppt, excellent selectivity against other VOCs (ethanol, toluene, xylene, methane), fast response time (~11 s), and high stability over time and under varying humidity conditions.

Kwon et al. [200] developed a photoactivated gas sensor system capable of real-time O_2_ monitoring at room temperature, specifically designed for hydrogen-rich, high-humidity environments such as water electrolysis systems. The sensor utilized In_2_O_3_ nanofilms fabricated by GLAD at θ=85°, followed by annealing at 400 °C to enhance crystallinity. To boost sensitivity and response speed, the surface was decorated with a 1 nm-thick layer of Cu NPs via electron beam evaporation. Under UV illumination (365 nm), the sensor enabled photoactivated room-temperature sensing, achieving stable and humidity-tolerant detection of O_2_ concentrations ranging from 0.5 to 2 vol% across a broad RH range (30–90%). Responses increased from approximately 2.06 to 3.34 with higher O_2_ levels. Although the baseline response time was relatively long (~500 s), the integration of a convolutional neural network (CNN) enabled rapid prediction of O_2_ concentration from early-stage transient responses, reducing the effective detection time to under 5 s while maintaining high accuracy.

**Metal doped.** Wongchoosuk et al. [111] developed C-doped WO_3_ NRs using GLAD for NO_2_ sensing. During the GLAD process (θ=85°), C_2_H_2_ gas was introduced into the sputtering chamber to achieve carbon doping. After deposition, all samples were annealed in air at 400 °C for 3 h to enhance crystallinity, with Auger electron spectroscopy confirming ~20.1 at.% carbon incorporation. Gas sensing experiments demonstrated that the C-doped WO_3_ NRs outperformed undoped counterparts in NO_2_ sensing, exhibiting higher sensitivity and faster response/recovery times. At an optimal operating temperature of 250 °C, the sensor showed a strong and selective response to NO_2_ within the 0.5–5 ppm range, with an estimated LoD below 0.5 ppm. Remarkably, the C-doped sensor maintained excellent performance at a lower operating temperature of 150 °C, achieving a response of 18.2 to 1 ppm NO_2_, surpassing previously reported WO_3_ NR sensors operating at higher temperatures. The enhancement in gas sensing performance is attributed to several synergistic effects resulting from carbon doping. First, carbon incorporation increased the aspect ratio and surface roughness, providing a larger active surface area for gas adsorption. Second, carbon doping reduced the activation energy required for charge carrier generation, thereby improving conductivity at lower operating temperatures. Finally, the doping altered the depletion layer structure, leading to more significant resistance modulation upon NO_2_ exposure. 

#### 3.1.4. Summary of GLAD-Based Resistive Gas Sensors

**Figure 11:**
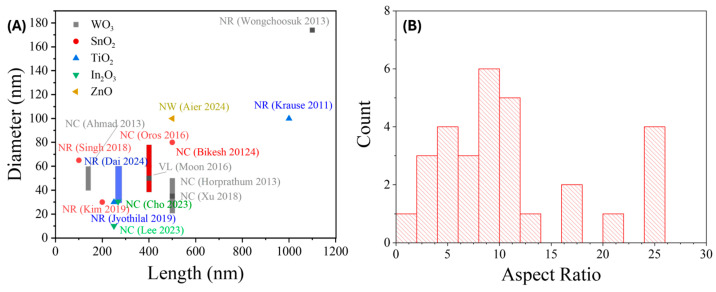
(**A**) The dimension of GLAD nanostructures used in literature [62,111,115,160,161,187,188,191,192,197,199,215,218,240,243] and (**B**) the corresponding aspect ratio statistics.

**Structure and morphology.** GLAD-fabricated nanostructures for resistive gas sensors exhibit a rich diversity of morphologies and dimensions, each tailored to optimize gas accessibility, surface area, and charge transport. Among the most common structures are NRs, also referred to as nanocolumns, which can be fabricated in both vertical and inclined orientations. These structures typically range in length from ~100 nm to over 1.5 μm, with diameters between 20 and 100 nm. Figure 11 presents the dimensions of GLAD NRs used in resistive gas sensor development and the distribution of the aspect ratio. Their porosity and anisotropy make them ideal for maximizing the gas–solid interface while providing tunable electrical pathways.

Short nanocolumns, those under 300 nm in length, are commonly found in In_2_O_3_ [196,239,240], SnO_2_ [160], and TiO_2_ [218] systems. For example, In_2_O_3_ columns with lengths around 250–268 nm and diameters as small as 10 nm have been reported, offering low to moderate resistance values depending on interconnectivity (e.g., 250 Ω to 1 MΩ) [196,239,240]. Similarly, SnO_2_ NRs of ~100 nm length and 65 nm diameter showed relatively high resistances (6.1–85.3 MΩ) [160], reflecting limited conduction paths in short, disconnected rods. Intermediate-length nanocolumns ranging from 300 to 500 nm are among the most frequently used across different materials. At these dimensions, a balance between enhanced connectivity and increased surface area is effectively achieved. WO_3_ nanocolumns with 400 nm length and ~50 nm diameter showed moderate resistance values (~1 MΩ) [113,187], while SnO_2_ NRs in the 400–450 nm range with 40–80 nm diameters exhibited resistances around 100 MΩ [161]. Pd decoration in this size regime can significantly lower resistance, as demonstrated by 200 nm-long SnO_2_/Pd rods (30 nm diameter) with a resistance of just 0.6 MΩ [115]. Longer nanocolumns, those exceeding 500 nm to over 1 μm, offer extended conduction pathways and higher surface roughness. Examples include WO_3_ rods of 500–1000 nm length and 20–50 nm diameter with resistance in the 10^4^–10^7^ Ω range [192], and Mg or TiO_2_ NRs reaching up to 1.5 μm in length [241,242]. In some cases, larger diameters (e.g., 174 nm WO_3_ rods at 1.1 μm length) correspond to significantly reduced resistance (e.g., 128 MΩ and 4.6 MΩ) [111], suggesting improved vertical connectivity.

Zig-zag NRs represent a distinct morphological category characterized by alternating growth directions, which increases surface curvature and adsorption sites. These structures typically range from 200 to 400 nm in length with diameters around 30–60 nm, although some reach 1 μm. Zig-zag TiO_2_ NRs with a length of 270 nm exhibited extremely low resistance (~0.01 MΩ) [188], while WO_3_ zig-zags at 500–1000 nm length showed resistance as low as 1 kΩ, demonstrating the effectiveness of this morphology in enhancing charge transport.

Villi-like structures, which resemble biological villi with highly branched tips, typically range from 100 to 400 nm in length and ~30–50 nm in diameter. These have been primarily realized in WO_3_ films. Their open and porous structure supports efficient gas infiltration. One such sensor with 380 nm villi-like rods showed a low resistance of ~0.1 MΩ [194], suitable for ambient NO_2_ detection.

Nanospirals and nanoscrews are more complex and less commonly reported. ZnO nanospirals (~400 nm in length) offer helical channels for gas diffusion and exhibit a resistance of ~1 MΩ [197]. WSe_2_ nanoscrews (~60 nm) represent a unique geometry that may provide new opportunities for hybrid gas sensing, though their electrical performance has not yet been detailed [136].

Across all these morphologies, length and aspect ratio play significant roles in determining electrical resistances, as illustrated in Figure 11. Shorter and more isolated nanostructures tend to exhibit higher resistance due to limited conduction paths, while longer, interconnected columns support lower resistance and higher sensitivity. The presence of metallic or plasmonic nanoparticle coatings (e.g., Pd, Au) further modifies the conductivity and enhances sensing performance through catalytic or photonic effects.

**Effects of annealing on GLAD deposited films.** Annealing plays a pivotal role in tuning the gas sensing performance of MOS nanostructures fabricated by GLAD. Due to the low temperature and physical nature of the GLAD process, the as-deposited films are typically amorphous or poorly crystalline, especially for materials like WO_3_ [187], TiO_2_ [244], and VO_x_ [245]. These amorphous structures generally exhibit very low electrical conductivity, which severely limits their usefulness in resistive gas sensing applications. Consequently, post-deposition annealing is a critical step to activate the nanostructured films by improving their crystallinity and enabling effective charge transport. Importantly, the specific crystalline phase formed upon annealing has a significant impact on the sensors’ performance and selectivity, as different polymorphs of a given MOS have varying surface reactivities, oxygen vacancy distributions, and adsorption affinities. For example, the monoclinic phase of WO_3_ is known for high NO_2_ sensitivity, while the anatase phase of TiO_2_ is generally more reactive than rutile in sensing applications [217]. Annealing also enables phase transitions that are beneficial for tuning these properties, depending on the target gas.

For metal-doped or metal-decorated GLAD nanostructures, annealing has further implications. It promotes the uniform distribution and crystallization of metal NPs, improves the interfacial bonding between the metal and host oxide, and reduces the defect density around dopants. This results in more stable and active catalytic sites, better electronic sensitization, and improved spillover effects, all of which enhance sensitivity and selectivity.

In terms of morphological effects, annealing alters the porosity, inter-nanorod connectivity, and overall surface area. Moderate annealing (typically 400–450 °C for 1–3 h) tends to enhance porosity by removing volatile residues and enabling partial grain boundary reorganization. This increases the number of active surface sites and facilitates faster gas diffusion. However, excessive annealing, particularly at temperatures exceeding 500 °C or for prolonged durations, may result in densification, grain growth, and collapse of the columnar or porous architecture. These effects reduce the available surface area and impede gas accessibility, ultimately degrading sensor response. Kiema et al. [246] showed that annealing GLAD-deposited TiO_2_ at 500 °C preserved the overall columnar morphology but significantly modified the internal structure. TEM analysis revealed that the fine substructures within each column coalesced, resulting in denser microstructures and reduced internal porosity. Similarly, Kay et al. [83] demonstrated that TiO_2_ films deposited at cryogenic temperatures (50–100 K) exhibited exceptionally high surface areas (~100 m^2^/g). TiO_2_ films deposited at low substrate temperatures (e.g., 50–100 K) were initially amorphous and exhibited very high surface area, up to ~100 m^2^/g. However, progressive annealing from 200 K to 1300 K caused a continuous decrease in surface area, with most porosity collapsing by 900 K. Notably, temperature-programmed desorption (TPD) experiments showed that moderate annealing (400–600 K) could enhance chemical reactivity by exposing higher-energy binding sites (such as Ti^4+^) without fully destroying the porous architecture. These findings emphasize that the thermal treatment conditions must be precisely controlled to optimize both the structural and functional properties of GLAD-grown nanomaterials for gas sensing applications.

Annealing also affects electrical conductivity, a key factor in resistive gas sensing [247,248]. By improving crystallinity and reducing trap states, annealing decreases resistivity and enhances carrier mobility, which helps to lower baseline resistance and amplify response changes upon gas exposure [249]. Additionally, increased grain connectivity resulting from annealing creates better percolation pathways for charge carriers [250].

However, the annealing window is narrow and must be carefully optimized. As seen in examples like TiO_2_, annealing at 500 °C not only alters internal porosity but also affects columnar stability. In contrast, studies such as those by Krause et al. [62,64] and Wongchoosuk et al. [111] highlight that mild annealing can promote beneficial surface restructuring and controlled void formation that enhances performance.

**Figure 12:**
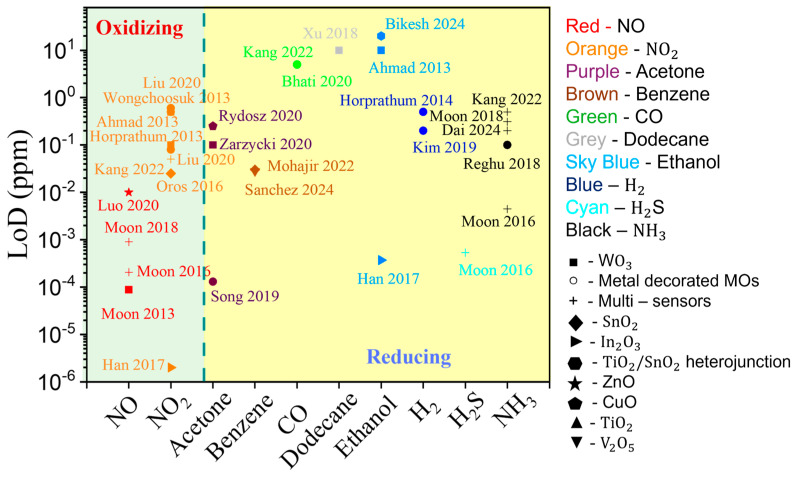
The summary of LoDs of different GLAD based MOS sensors [73,111,113,114,115,118,119,129,131,143,161,187,188,191,192,194,195,198,199,215,237,238,251,252].

**Sensor Performance Summary**. Gas sensor performance is primarily evaluated based on LoD, sensitivity (response), and response/recovery time. Figure 12 summarizes reported LoDs for a variety of GLAD-fabricated MOS-based sensors across both oxidizing and reducing gases. It clearly illustrates how sensing performance strongly depends on both the intrinsic material properties and the nanostructured morphology enabled by GLAD.

For oxidizing gases, NO_2_ sensing shows the most exceptional results. Vertically aligned In_2_O_3_ nanocolumns exhibit the lowest reported LoD at 2 ppt [73], followed by SnO_2_ nanocolumns with a LoD of 0.025 ppm [161]. These values significantly outperform traditional morphologies and materials, such as bare WO_3_ NRs with higher LoDs of 0.1 ppm [187] and 0.5 ppm [191]. Material modifications also prove effective: Pt-decorated WO_3_ achieves an LoD of 0.08 ppm [113], Pd-decorated WO_3_ reports 0.6 ppm [118], and carbon-doped WO_3_ reaches 0.5 ppm [111]. These enhanced performances are primarily attributed to the n-type semiconducting nature of the materials, which offers favorable band alignment with oxidizing gases like NO_2_, as well as the highly porous nanostructures that maximize gas adsorption.

NO detection is of particular interest for biomedical applications such as breath analysis, where NO serves as a biomarker for inflammation and respiratory disease. Therefore, achieving a low LoD becomes a critical performance parameter. Moon et al. [194,215] demonstrated an electronic nose platform with LoDs of 88 ppt [194] and 0.2 ppb [215]. In subsequent work, they fabricated a SnO_2_/WO_3_-based sensor array capable of detecting NO at 0.9 ppb under 80% relative humidity [238]. Another notable design includes ZnO nanospirals, which reached a LoD of 0.01 ppm [198].

Among reducing gases, ethanol detection is led by In_2_O_3_, which achieved a LoD of 0.37 ppb [73]. This is vastly superior to other materials such as WO_3_ (10 ppm) [191] and TiO_2_/SnO_2_ hybrids (20 ppm) [199]. The outstanding performance is attributed to the high oxygen-vacancy density and large accessible surface area provided by GLAD-fabricated nanostructures. For acetone, Rh-decorated WO_3_ NRs exhibit the best performance with an LoD of 13 ppb, a significant improvement over bare WO_3_ NRs (0.25 ppm) [195] and CuO-based sensors (0.1 ppm) [129].

Benzene sensing is effectively achieved using a nanosculptured V_2_O_5_ film, which recorded an LoD of 0.02 ppm [131], slightly better than a comparable SnO_2_-based sensor with a LoD of 0.03 ppm [143]. For NH_3_, Moon et al.’s [215] electronic nose again demonstrated impressive performance with a LoD of 4 ppb. Additional systems include Au-decorated WO_3_ nanostructures (100 ppb) [251], zigzag TiO_2_ NRs (200 ppb), and multi-sensor platforms reporting LoDs of 0.31 ppm [238] and 0.5 ppm [237].

Finally, heterostructured or multicomponent sensors, often based on material arrays or engineered interfaces, set the benchmark for sensing gases like acetone [119], hydrogen [114,115], carbon monoxide [237,252], and hydrogen sulfide [215]. These architectures offer enhanced performance due to synergistic interactions at material interfaces, which create complementary transduction pathways and unique electronic states. These effects collectively enable LoDs significantly lower than those observed in single-phase or unmodified materials. As shown in Figure 12, many of the top-performing sensors across all target gases fall into this multicomponent category, underscoring the critical role of interface engineering and material hybridization in next-generation gas sensor design.

**Figure 13:**
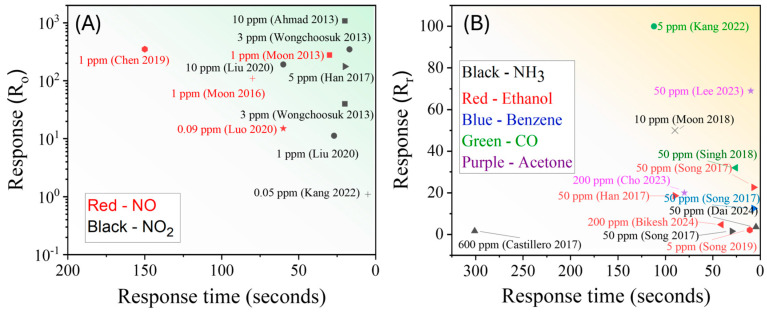
The summary of response versus response time of different GLAD-based MOS sensors for (**A**) oxidizing gases, NO and NO_2_ [73,111,113,118,136,191,194,198,215,237], and (**B**) reducing gases, NH_3_, Ethanol, Benzene, CO, and Acetone [73,119,160,188,196,199,237,238,240,243,253].

To further evaluate sensor effectiveness, it is essential to consider the trade-off between response magnitude and response time. Figure 13A,B plots these two parameters for various GLAD-fabricated MOS sensors across oxidizing and reducing gases.

Figure 13A focuses on oxidizing gases, particularly NO (in red) and NO_2_ (in black). Among NO sensors, the most notable is the villi-like WO_3_ nanostructure developed by Moon et al. [194], which achieved a remarkably high response of approximately 278 in just 30 s. This architecture benefits from a 32-fold increase in surface area compared to planar films and features narrow necks between nanofingers that act as electronic constriction points. These features amplify resistance changes caused by small fluctuations in carrier concentration. The favorable electronic band structure of WO_3_ supports efficient electron withdrawal by oxidizing gases like NO, while the 1D nanostructure maintains excellent carrier mobility and rapid gas diffusion. Another effective NO detection system is a multi-material electronic nose composed of Au-decorated SnO_2_, WO_3_, and In_2_O_3_ nanostructures, which showed a high response of 110 in 80 s [215], illustrating the synergistic benefits of combining multiple materials. Additionally, WSe_2_ nanoscrews achieved the highest NO response of 350 in 150 s [136], attributed to their p-type semiconducting nature and highly efficient charge transfer to NO molecules. ZnO nanospirals also performed well, showing a moderate response of ~15 within 60 s [198]. The wurtzite structure of ZnO, which is rich in oxygen vacancies, promotes NO adsorption and charge transfer, while its spiral morphology ensures a large, accessible surface without compromising diffusion speed.

For NO_2_, WO_3_ nanostructures again dominate. The highest reported response value of 1075 was achieved by WO_3_ NRs in just 20 s [191], making them the most responsive NO_2_ sensors in the dataset. This exceptional performance stems from the strong interaction of WO_3_ with oxidizing gases and its GLAD-induced porosity. Carbon doping further enhances the sensitivity of WO_3_ by showing a response of 348 in only 17 s, outperforming their undoped counterparts (response of 40 in 20 s) [111]. Metal decoration also impacts performance: Pd-decorated WO_3_ NRs achieved a response of 191 in 60 s [118], while Pt-decorated WO_3_ yielded a more modest response of 11.24 in 27 s [113], indicating the catalytic influence of specific metal types. A multi-sensor array configuration offered ultra-fast detection (~5 s) with moderate response, ideal for real-time applications [237]. In_2_O_3_ nanocolumns also demonstrated strong performance with a response of 176 in just 20 s [73].

Figure 13B presents the performance map for reducing gases, including NH_3_, CO, ethanol, acetone, and benzene. For NH_3_, TiO_2_-based sensors are most common but vary widely in performance. Zigzag TiO_2_ NRs responded to 50 ppm NH_3_ within 4.5 s, albeit with low signal amplitude [188]. In contrast, a villi-like WO_3_–SnO_2_ sensor array exhibited a much higher response of 50 but required 50 s [238]. An optical NH_3_ sensor using TiO_2_/porphyrin structures performed poorly, reaching a response of only 1.6 over 301 s, confirming the superior kinetics of resistive sensors [253]. In_2_O_3_ nanocolumns offered a good balance, achieving a response of 22.6 in 7 s [196].

For CO, SnO_2_ nanocolumns demonstrated a response of 100 in 112 s [160], while another SnO_2_-based sensor achieved a response of 32 in 26 s [237]. In ethanol detection, In_2_O_3_ nanostructures again performed well, with one study reporting a response of 22.6 in 7 s [196] and another showing 18.5 in 90 s [240]. These results are consistent with the known affinity of In_2_O_3_ for alcohols. Notably, heterojunction architectures improve performance further; Rh-decorated WO_3_ NRs [119] and TiO_2_/SnO_2_ heterostructures [199] both demonstrated rapid response and strong signal.

In the case of acetone, Au-decorated In_2_O_3_ sensors performed exceptionally well, achieving response values of 20 [240] and 69 [243] in 80 and 10 s, respectively. These results highlight the role of catalytic metal islands in accelerating gas adsorption and charge-transfer processes. For benzene detection, In_2_O_3_-based sensors detected 50 ppm benzene in 7 s with a response of 12.5 [196], indicating a relatively fast detection speed for this aromatic target.

Importantly, the bottom-right region of Figure 13B shows a dense cluster of sensors across various reducing gases that achieve fast response times (under 60 s) accompanied by moderate response values (10–30%). This grouping reflects a class of sensors deliberately optimized for rapid detection rather than maximum sensitivity. These devices are particularly suited for real-time monitoring, where timely detection is critical. Despite their modest signal magnitudes, they deliver reliable performance across different materials, including In_2_O_3_, SnO_2_, WO_3_, and metal-decorated MOS platforms. This tight clustering also highlights how GLAD-fabricated nanostructures enable precise tuning of porosity, morphology, and composition to consistently achieve fast response kinetics.

This range of sensor designs illustrates a key engineering advantage of GLAD: it enables a trade-off regime where fast, moderate-response sensors can be deployed for safety-critical and air-quality monitoring scenarios, where speed and consistency are often more valuable than absolute sensitivity.

### 3.2. Capacitive Gas Sensors

Capacitive gas sensors operate based on the principle of a parallel-plate capacitor, where two conductive electrodes are separated by a dielectric material. The capacitance of this system is defined by [254],

(18) $$C=\varepsilon_{0}\varepsilon_{r}\frac{A}{d},$$

where $\varepsilon_{0}$ is the permittivity in vacuum, $\varepsilon_{r}$ is the relative permittivity (dielectric constant) of the dielectric material, *A* is the electrode area, and *d* is the thickness of the dielectric layer or the separation distance between the electrodes. In gas sensing applications, since $\varepsilon_{0}$ is a constant and the electrode area *A* typically remains fixed, gas-induced changes are primarily detected through variations in $\varepsilon_{r}$ and/or *d*. Thus, capacitive gas sensors are broadly classified into two types: permittivity-change-based sensors, and thickness-change-based sensors [254].

Capacitive gas sensors offer several advantages over resistive sensors, particularly in low-power, room-temperature operation [255,256]. They are highly sensitive, capable of detecting even monolayer adsorption, and exhibit fast, reversible responses. Unlike resistive sensors, which rely on changes in electrical conductivity caused by charge transfer and often require elevated temperatures to facilitate redox reactions, capacitive sensors function efficiently at ambient conditions, making them more suitable for integration into miniaturized and flexible electronics. They also provide enhanced selectivity when designed with gas-specific dielectric interfaces. Although resistive sensors benefit from simpler readout electronics, capacitive sensors can yield richer analytical data through techniques like impedance spectroscopy, which reflects both capacitive and resistive changes during gas exposure.

The response $R_{C}$ of a capacitive gas sensor is often expressed as a change in capacitance relative to its baseline value [254],

(19) $$R_{C}=\frac{∆C}{C_{0}},$$

and the sensitivity (*S*) is defined as [254],

(20) $$S=10\times \log\left(\frac{C_{gas}}{C_{0}}\right).$$

**Figure 14:**
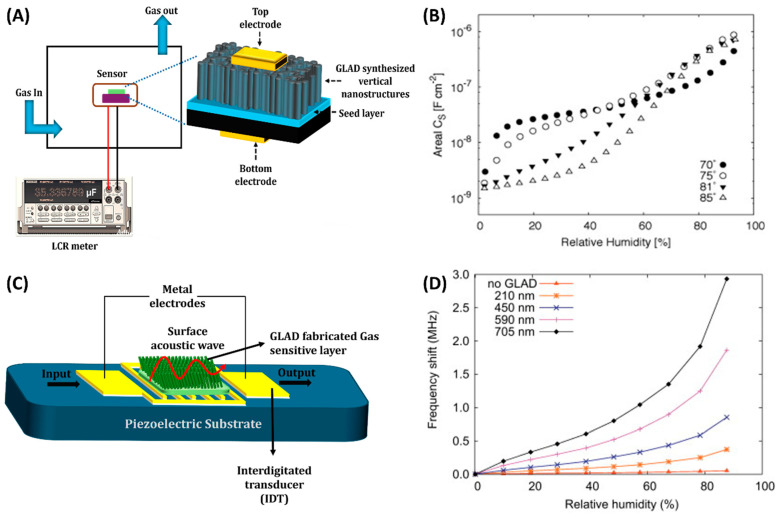
(**A**) A typical capacitive gas sensor configuration based on GLAD NRs. (**B**) Capacitive response for sensors coated with TiO_2_ films at different deposition angles. Reproduced from [258] with permission from Elsevier. (**C**) A typical SAW sensor integrated with GLAD nanostructures. (**D**) Response of SAW sensors to RH loading, coated with different lengths of GLAD SiO_2_ NRs. Reproduced from [259] with permission from Elsevier.

Capacitive measurements may be taken directly or interpreted through impedance analysis [255]. The LoD depends on the ability to resolve small changes in capacitance above background noise. Response and recovery times are governed by the dynamics of gas adsorption/desorption and the dielectric relaxation of the sensing material. Importantly, capacitive sensors are associated with minimal energy dissipation, as the sensing mechanism involves no active current flow. This allows for ultra-low power operation while still producing a useful and sensitive analytical signal [254,256]. Over the past 25 years, GLAD has emerged as a powerful and versatile technique for fabricating high-performance capacitive humidity sensors (Table 6) [257]. A typical GLAD-based capacitive sensor is shown in the schematic (Figure 14A), where the GLAD nanostructure is positioned between the top and bottom electrodes. The development of GLAD-based humidity sensors from 1999 to 2024 reveals that sensor performance depends on several key factors: the choice of sensing material, deposition angle, nanocolumn height and porosity, electrode material, and advanced post-fabrication techniques. 

**Table 6:** Summary of GLAD-based capacitive and piezoelectric sensors.

Mat	Structure	Deposition Condition	Target Gas	Response and Sensitivity	$t_{90}\;\&\;t_{10}$	Operation Frequency	Ref
**Capacitive**							
$TiO_{2}$	Vertical columns	E-beam: θ=81°	Humidity	S (nF/RH%): 34.4 @ 78% RH	$t_{90}\approx 275\;ms,\;t_{10}\approx 297\;ms$	1 kHz@1 $V_{rms}$	[144]
	Vertical columns	E-beam evaporation, θ = 60–80°;	Humidity	Ti electrodes: C ≈ 233–1050 pF at 11–93% RH TiN electrodes: C ≈ 375 pF at 11–52% RH & abrupt >52%	N/A	20 Hz to 2 MHz, V = −3 V to 3 V	[260]
	Vertical columns	E-beam evaporation, θ = 81°	Humidity	C ≈ 2–1000 ${nF/cm}^{2}$ @ 0–92%RH	$t_{90}\approx 50\;s,\;t_{10}\approx 129\pm 15\;s$ (78% RH)	25 Hz, 1 $V_{rms}$, @ 20 °C to 22 °C	[261]
	Vertical columns	PVD: θ = 81°	Humidity	C ≈ 1–1000 ${nF/cm}^{2}$ @ 0–92%RH	$t_{90}<220\;ms,\;t_{10}<400\;ms$	1 kHz, 1 $V_{rms}$	[262]
	Vertical columns	E-beam evaporation θ = 80°	Humidity	C ≈ 2–2200 ${nF/cm}^{2}$nF @ 2–92%RH	N/A	20 Hz to 1 MHz, 1 $V_{rms}$	[263]
	Vertical columns	E-beam evaporation θ = 70–85°	Humidity	C ≈ 1–800 ${nF/cm}^{2}$ @ 2–95%RH	$t_{90}\approx 388-176\;ms$ $t_{10}\approx 469-239\;ms$	1 kHz, 1 $V_{rms}$	[258]
	Vertical columns	E-beam: θ = 81°	Humidity	C ≈ 1–1600 nF @ 6–92%RH	N/A	1 kHz, 1 $V_{rms}$	[264]
	Vertical columns	E-beam evaporation, θ = 81°	Humidity	C ≈ 2–1600$\;{nF/cm}^{2}$ @ 2–92%RH (untreated) C ≈ 2–14,000$\;{nF/cm}^{2}$ @ 2–92%RH (treated)	NA	1 kHz, 1 $V_{rms}$	[242]
$SiO_{2}$	Vertical columns	E-beam: θ=81°	Humidity	S(nF/RH%): MAX @ 55% RH	$t_{90}\approx 231\;ms,\;t_{10}\approx 229\;ms$	1 kHz at 1 $V_{rms}$	[144]
	Vertical columns	E-beam evaporation θ=81°	Alcohols	C ≈ 1–2000 nF (0–6.5% Ethanol), C ≈ 3–7000 nF (0–14.1% Methanol), C ≈ 0.7–300 nF (0–2.8% 1-PrOH), C ≈ 2–300 nF (0–3.4% 2-PrOH), C ≈ 0.7–15 nF (0–6.8% 1-BuOH), LoD =190 ppm (Ethanol)	$t_{90}=66\;s$, $t_{10}=76\;s$	20 Hz to 1 kHz, 22 °C	[265]
**SiO**	Helical columns	Thermal evaporation	Humidity	C = 0.063–425 nF@1.1–97% RH	$t_{90}=3\;s,\;t_{10}=NA$	120 Hz at 23 °C	[257]
	Helical columns templates	PVD θ = 85°	Humidity	C ≈ 0.120 nF@15–65% RH C ≈ 0.120–1.240 nF @ 65–90% RH	$t_{90}=75\;s,\;t_{10}=175\;s$	NA	[266]
**Si**	Vertical columns	E-beam: θ = 81°	Humidity	C ≈ 2–6400 ${nF/cm}^{2}$ @ 2–90%RH	NA	1 kHz, 1 $V_{rms}$	[242]
$Al_{2}O_{3}$	Vertical columns	E-beam evaporation: θ=81°	Humidity	S(nF/RH%): MAX @ 80% RH	$t_{90}\approx 87\;ms,\;t_{10}\approx 104\;ms$	1 kHz@1 $V_{rms}$	[144]
	Vertical columns	E-beam evaporation: θ = 60–85°	Humidity	C ≈ 0.060 @ 10–50%RH C ≈ 0.060–0.950 nF @ 50–85%RH	$t_{10}\approx 40\;ms$ (θ = 85°) $t_{10}\approx 100\;ms$ (θ = 75°)	1 kHz	[267]
**Piezoelectric**							
SiO **_2_**	Vertical columns	E-beam: θ = 70°	Humidity	S = 15.50–65.4 kHz/%RH @ 25–75% RH	N/A	~123.3 MHz	[259]

Among the many nanostructured humidity sensors developed over the past two decades, GLAD-based TiO_2_ sensors fabricated with Ti IDEs and θ=70° currently represent the state-of-the-art, achieving a sensitivity of 1.17 × 10^−11^ F/%RH and excellent linearity ($R^{2}=0.97$) at a 100 Hz signal frequency [260]. Studies have shown optimal structures are formed at glancing angles of 70° to 85°, which produce vertically aligned columns with open diffusion paths, resulting in subsecond response times with high capacitive response observed for varying humidity (Figure 14B) [258]. Furthermore, Steele et al. [144] investigated various GLAD-fabricated nanostructured materials, including TiO_2_, Al_2_O_3_, and SiO_2_, for their application as capacitive humidity sensors. The TiO_2_-based sensor exhibits the highest sensitivity across the entire RH range, peaking at 34.4 nF/%RH around 78% RH. In addition to TiO_2_, Al_2_O_3_ and SiO_2_ show lower maximum sensitivities, peaking near 80% and 55% RH, respectively. This variation with RH reflects the exponential relationship between capacitance and humidity in these materials. On the other hand, reactive ion etched (RIE) capacitive sensors achieved response times as fast as 50 ms while maintaining high sensitivity [261]. Together, these findings highlight the versatility and strong performance of GLAD-fabricated humidity sensors.

The choice of sensing material critically influences sensor performance. Early sensors used SiO and SiO_2_ [257,266], which offered moderate sensitivity and stability but lacked surface chemistry conducive to regeneration. Metal oxides like Al_2_O_3_ and especially TiO_2_ emerged as superior materials, offering enhanced adsorption sites due to surface hydroxylation, strong dielectric response to moisture, and photocatalytic properties that enable UV-based regeneration [144,262,263,267]. TiO_2_ sensors typically exhibit capacitance increases of up to 1000× from low to high humidity and retain performance over extended RH cycling.

Film morphology, especially porosity and nanocolumn topology, is directly controlled through deposition angle and film thickness. Among the studied structures, straight vertical NRs consistently deliver the best results. Sensors based on these structures exhibit subsecond response times, high sensitivity, and minimal hysteresis [144,258,267]. In contrast, helical nanostructures introduce more tortuous diffusion pathways. This results in slower response times (up to 75 s) and reduced sensitivity, as observed in early SiO-based sensors [257]. The limited porosity and restricted top access of helical structures make them less suitable for rapid humidity sensing. A direct comparison by Wu et al. [257] between helical and post-like (straight column) SiO films confirmed that post-like structures outperform helical ones in terms of both sensitivity and response time. This conclusion has been consistently supported by subsequent studies using TiO_2_ and Al_2_O_3_ [144]. Empirically, response time scales linearly with thickness up to ~4 µm [258],

(21) $$\tau \left(T\right)\approx \left(162\;\frac{ms}{\mu m}\right)T-(16\frac{ms}{{}^{\circ}})(\theta -81{}^{\circ}),$$

but becomes sublinear above this limit due to morphological broadening near the film surface. Etching techniques, such as RIE, further enhance performance by opening intercolumn diffusion channels, producing adsorption response times as short as 50 ms [261].

Electrode material and geometry also play critical roles. IDEs, especially countersunk designs, ensure full engagement with the nanostructured films and minimize deposition shadowing [262]. Recent work comparing Ti and TiN IDEs found that Ti electrodes significantly outperform TiN, offering smoother capacitance-RH–RH curves and improved linearity [260]. Smaller pitch sizes and larger electrode areas further enhance sensitivity due to stronger electric field overlap and increased effective sensing volume [262].

A unique advantage of TiO_2_-based GLAD sensors is their self-regenerating capability via photocatalytic UV treatment. After several weeks of aging and performance decline, TiO_2_ sensors can be fully rejuvenated by UV exposure, particularly at short wavelengths (254–295 nm), restoring sensitivity and reducing hysteresis [263,264]. Regeneration has been demonstrated with both mercury lamps and UV LEDs, enabling compact and efficient implementation.

To further enhance performance and enable new sensor functionalities, post-fabrication strategies have been proposed. One notable method is ALD of ultrathin TiO_2_ onto GLAD-fabricated scaffolds made of other materials (e.g., Si) [242]. Even a 0.3–0.5 nm TiO_2_ coating is sufficient to mimic the full sensing and regeneration behavior of TiO_2_ films, while preserving the mechanical properties and porosity of the original structure. This decouples the roles of morphology and surface chemistry, offering new flexibility in sensor design.

In addition to humidity sensing, GLAD-based capacitive sensors have also been adapted for room-temperature detection of alcohol vapors. Beckers et al. [265] demonstrated this by fabricating a sensor comprising 1500 nm-thick vertically aligned SiO_2_ NRs deposited onto IDEs at θ=81°. Rather than relying on an array of materials, they employed a single sensor with multi-frequency impedance measurements to function as a virtual sensor array, capable of distinguishing between different alcohol species. The sensor was tested at room temperature (22 °C) against various alcohol vapors, including ethanol, methanol, 1-propanol, 2-propanol, and 1-butanol, by measuring both capacitance and impedance across a frequency range of 20 Hz to 1 kHz. It showed consistent and reproducible responses for all tested vapors, with a LoD for ethanol of 1.9 × 10^−4^ mole fraction under UV-LED illumination, and 3.4 × 10^−4^ without it. The response and recovery times were approximately 66 s (10–90%) and 76 s (90–10%), respectively, at 20 Hz. A notable feature of the sensor was its UV-assisted regeneration capability. Illumination at 351 nm significantly prolonged sensor life by promoting photocatalytic desorption of strongly adsorbed alcohol molecules. For ethanol and 1-butanol, the UV-LED extended the operational lifetime of the sensor from ~8 h to approximately 67 h, an eightfold improvement. In contrast, UV treatment had a limited effect on the responses to methanol, 1-propanol, and 2-propanol, indicating that desorption efficiency is analyte-specific. To achieve selectivity, the authors developed a database of standard frequency response curves and applied a matching algorithm to identify alcohol type and concentration. In blind tests, the system correctly identified the analyte in 5 out of 7 cases, particularly for methanol, ethanol, and 2-propanol. However, it struggled to differentiate between 1-propanol and 1-butanol, likely due to the similarity in their impedance signatures. This study illustrates the potential of GLAD-fabricated sensors as compact, room-temperature, and regenerable platforms for selective vapor detection using frequency-domain analysis.

### 3.3. Piezoelectric-Based Gas Sensors

Another widely used gas sensing mechanism relies on the oscillation of a piezoelectric crystal, where the intrinsic resonant frequency shifts upon gas molecule adsorption on the sensor surface. This mass-loading effect alters the vibrational characteristics of the crystal, allowing for highly sensitive detection of trace gases through frequency monitoring [7,268]. When gas molecules bind to a functionalized or selective sensing film coated onto the resonator surface, the added mass causes a measurable drop in resonant frequency, forming the basis of quantitative gas detection. Two of the most common piezoelectric sensor platforms that utilize this principle are thickness shear mode (TSM) resonators, also known as quartz crystal microbalances (QCM) or bulk acoustic wave (BAW) devices, and surface acoustic wave (SAW) resonators. The frequency shift ∆F due to mass loading is described by the Sauerbrey equation [7],

(22) $$∆F=2.3\times 10^{6}\times F^{2}\left(\frac{∆M}{A}\right),$$

where *F* is the fundamental frequency, ΔM is the added mass due to gas adsorption, and *A* is the active surface area. This frequency shift can be further used to calculate the specific molar sensitivity $S_{mol}$, a measure of sensor performance [7],

(23) $$S_{mol}=\frac{∆F\times M}{c\times m_{f}},$$

where M is the molar mass of the analyte (g/mol), c is its concentration (g/m^3^), and $m_{f}$ is the mass of the sorbent film. The selectivity and sensitivity of piezoelectric gas sensors are determined by both the chemical composition of the coating and the operating frequency of the device. Functionalizing the membrane with specific chemical groups allows targeting different gas species. These sensors offer advantages such as low operating temperatures and extended lifetimes, but typically have moderate sensitivity and are influenced by environmental conditions. The LoD depends on factors like resonator sensitivity to mass changes, frequency stability, and the strength of gas–film interactions.

Compared to resistive and capacitive gas sensors, piezoelectric gas sensors offer several distinct advantages, including high sensitivity, low power consumption, and stable operation at ambient temperatures, making them particularly attractive for portable and battery-powered applications. Their long-term stability is further supported using inert substrates and low-temperature operation. However, their performance can be affected by environmental factors such as humidity and temperature, and they require precise instrumentation to detect small shifts in resonant frequency. Despite these challenges, with the use of well-engineered surface coatings, piezoelectric sensors can achieve an optimal balance between sensitivity, energy efficiency, and chemical selectivity.

The sensing layer plays a central role in enabling reliable gas detection. It must provide a high surface area and strong affinity for target gas molecules to ensure effective adsorption and produce a detectable frequency shift. To enhance selectivity, the layer can be chemically tailored using polymers, nanomaterials, or specific functional groups. In addition, the coating should be thin, uniform, and mechanically stable to support acoustic wave propagation without introducing significant damping. It must also allow reversible adsorption, adhere well to the substrate, and be compatible with standard fabrication techniques.

As discussed in Section 2, GLAD-fabricated nanostructures meet many of these key requirements. Their tunable porosity, large surface area, and mechanical stability make them especially suitable as high-performance sensing layers in piezoelectric gas sensors (see Figure 14C for a typical configuration). However, there are only a few studies on using GLAD nanostructures to enhance the performance of piezoelectric sensors (see Table 6) [259,269,270,271].

Kwan and Sit [259] developed Love-wave SAW humidity sensors based on vertical SiO_2_ nanocolumns fabricated by GLAD at θ=70°, with film thicknesses ranging from 200 to 705 nm. The sensors are built on ST-cut quartz substrates with a polymethyl methacrylate (PMMA) waveguiding layer, operating at approximately 120 MHz. Figure 14D illustrates the frequency shift of Love-wave SAW humidity sensors coated with GLAD SiO_2_ nanostructures of varying thicknesses in response to RH ranging from 0% to 90%. All GLAD-coated devices show significantly larger frequency shifts compared to the uncoated reference device, with the magnitude of the shift increasing with film thickness. This trend demonstrates the increased surface area and water adsorption capacity provided by thicker nanostructured films, confirming their effectiveness in boosting sensor sensitivity to humidity. The GLAD-coated devices also demonstrated good long-term stability, with a 500 nm-coated sensor maintaining consistent performance over 21 days in ambient air. However, increasing the thickness of the GLAD films beyond 300 nm resulted in a rise in insertion loss, attributed to the viscoelastic nature of the thicker coatings and possible aging effects. A control study confirmed that the improved sensitivity was due to the nanostructured GLAD coating rather than increased waveguide thickness, as a device with a thicker PMMA guiding layer showed lower sensitivity than even the thinnest GLAD-coated sensor.

### 3.4. Optical Gas Sensors

Optical gas sensors offer several advantages over traditional resistive, capacitive, and piezoelectric sensors. They achieve ppt-levels of detection limits and high selectivity by detecting unique molecular absorption or vibrational features, enabling precise analyte identification [5]. Unlike resistive sensors that require high operating temperatures, optical sensors typically operate at room temperature, resulting in lower power consumption and safer operation, especially for flammable gases [272]. They also support remote and non-contact detection, multiplexed data outputs, and seamless integration with machine learning for real-time classification and quantification. These features make optical gas sensors ideal for low-power, portable, and intelligent sensing platforms [273]. GLAD-based nanostructures have been successfully implemented in a range of optical gas sensing modalities, most notably in optical absorption and surface-enhanced Raman spectroscopy (SERS). Table 7 presents a summary of GLAD-based optical gas sensors reported in the literature.

#### 3.4.1. Optical Absorption Spectroscopy

Optical absorption spectroscopy-based gas sensors detect gases by measuring the amount of light absorbed as it passes through a gas sample. Many gases have unique absorption features at specific wavelengths in the ultraviolet (UV), visible, or infrared (IR) regions of the electromagnetic spectrum. When a beam of light from a laser or broadband source is directed through a gas, certain wavelengths are absorbed by the gas molecules due to their characteristic electronic, vibrational, or rotational transitions. A photodetector then measures the intensity of the transmitted light, and the difference between the incident and transmitted intensities indicates the amount of absorption. This absorption is directly related to the gas concentration, as described by Beer–Lambert’s law, which links absorbance A to the concentration c of the gas, its molar absorptivity, and the optical path length [5],

(24) $$A=ln(I_{0}/I)=\varepsilon \cdot c\cdot l,$$

where $I_{0}$ and I are the incident and transmitted light intensity, ε is the molar absorptivity, and l is the optical path length. Optical absorption spectroscopy offers several advantages, including high specificity, real-time and non-invasive measurement, and the ability to detect both trace and high concentrations of gases. This technique is commonly used to detect gases such as CO_2_, CH_4_, NO_2_, SO_2_, O_3_, NH_3_, and various VOCs.

**Figure 15:**
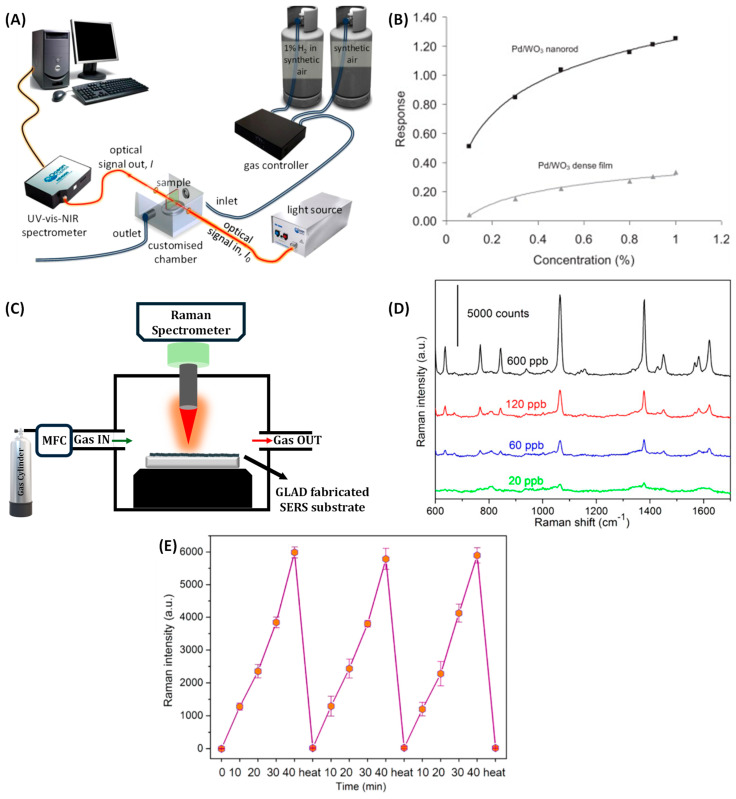
(**A**) The experimental setup and (**B**) Sensor response of Pd/WO_3_ dense film and NRs toward various H_2_ concentrations at 100 °C. Reproduced from [274] with permission from Elsevier. (**C**) A typical SERS gas sensor setup integrated with GLAD SERS substrate. (**D**) SERS spectra of different concentrations of 2-NAT collected on the AgNRs@HfO2 substrate after 40 min of gas flow, and (**E**) The peak intensity variations at 1379 cm^−1^ of 600 ppb 2-NAT during the repetition of “vapor exposure−thermal cleaning” cycles. Reproduced from [275] with permission from the American Chemical Society.

Two studies have directly reported optical absorption spectroscopy-based gas sensors using GLAD nanostructures. Wisitsoraat et al. [274] developed a Pd-coated WO_3_ NR sensor via GLAD sputtering for H_2_ detection, leveraging the gasochromic response of WO_3_. As illustrated in Figure 15A, the setup features a heated Pd/WO_3_ sensor exposed to controlled H_2_ concentrations, with real-time absorbance (650–1000 nm) monitored using a fiber-optic spectrometer. To enhance catalytic interaction with H_2_, a thin Pd layer (~2.5 nm) was coated on the WO_3_ NRs. Figure 15B illustrates the sensor response versus H_2_ concentration (0.1–1%) at 100 °C, where the NR sensor outperforms dense films showing a response of 0.51 at 0.1% H_2_, over 10× greater than the dense film. The response followed a logarithmic trend, with fast kinetics (~60 s response, ~90 s recovery at 1% H_2_), demonstrating high sensitivity and capability for low-concentration detection. Castillero et al. [253] developed a GLAD-fabricated optical gas sensor for detecting ammonia and volatile amines using protonated porphyrin-functionalized TiO_2_ thin films. The sensor operates via reversible deprotonation of cationic porphyrins, leading to measurable changes in fluorescence and UV–vis absorption. Two porphyrins, MMPyP and TMPyP, were studied; MMPyP/TiO_2_ outperformed TMPyP with a faster response time (*t*_90_ = 247 s) and a lower LoD (0.05 ppm), attributed to favorable J-type aggregation that enhances gas diffusion. The sensor also showed better selectivity toward more volatile amines.

#### 3.4.2. Surface-Enhanced Raman Spectroscopy

Surface-enhanced Raman spectroscopy (SERS) is a powerful analytical technique that enhances the inherently weak Raman scattering signals of molecules adsorbed on or near nanostructured metallic surfaces [279]. This enhancement primarily arises from the localized surface plasmon resonance (LSPR) of metallic nanostructures, which amplifies the local electromagnetic field at the surface and boosts the Raman signal by several orders of magnitude [280]. Among various SERS-active substrates, silver NRs (Ag NRs) fabricated via OAD have gained particular attention due to their high aspect ratio, strong plasmonic behavior, and tunable morphology [281]. These vertically aligned NR arrays provide a dense network of electromagnetic “hot spots” that are ideal for SERS enhancement. Their compatibility with surface functionalization and optical detection techniques makes Ag NRs especially suitable for vapor-phase sensing of gases and VOCs.

A typical SERS-based gas sensing setup is shown in Figure 15C, where the SERS substrate is placed inside a controlled gas chamber with a top window for laser excitation and signal collection. Several studies have explored Ag NR-based SERS substrates for gas detection [276,282,283]. Shah et al. [276] demonstrated that cryogenically grown Ag NRs (~100 K) exhibit enhanced SERS performance compared to room-temperature-grown structures, offering ~3× higher enhancement factors and significantly improved detection of 4-aminobenzenethiol (4-ABT) vapor within 5 min, with detection limits in the low ppm range. To improve durability and reusability, Ma et al. [275] coated Ag NRs with a ~1.6 nm HfO_2_ shell via ALD, creating a recyclable substrate. Figure 15D presents the SERS spectra of vapor-phase 2-naphthalenethiol (2-NAT) at various concentrations collected after 40 min of gas flow over the Ag NRs@HfO_2_ substrate. The spectra clearly demonstrate that the substrate can detect 2-NAT down to 20 ppb with strong and well-defined Raman signals. Moreover, the substrate maintained its sensing performance through multiple “vapor exposure–thermal cleaning” cycles, wherein adsorbed analytes were removed by briefly heating the substrate at 250 °C (Figure 15E). This recyclability highlights the practical potential of the sensor for repeated use without significant signal degradation. In addition to detecting single analytes, the Ag NRs@HfO_2_ substrate also demonstrated the ability to distinguish and simultaneously detect vapor mixtures, such as 2-NAT and 2-mercaptopyridine (2MPy). Gahlaut et al. [277] developed Ag–Ag_2_S nanoheterostructures by exposing Ag NRs to ambient H_2_S in sewage gas. These structures retained SERS functionality and exhibited enhanced adsorption, with additional applications in photocatalysis and antibacterial surfaces.

To achieve ultra-sensitive VOC detection, Oh et al. [278] introduced a deep-cooling strategy, in which Ag NRs functionalized with propanethiol were cooled to −80 °C using a thermoelectric cooler. This suppressed benzene desorption and improved adsorption, resulting in over 1000× signal enhancement and a LoD as low as ~1 ppb, representing one of the best reported values for SERS-based gas sensors. The platform supported rapid, reversible sensing and was supported by theoretical modeling based on Langmuir and Maxwell–Boltzmann adsorption kinetics [278].

Ag NR-based SERS gas sensors demonstrate high sensitivity (ppb–ppm), strong selectivity, especially for sulfur-containing gases, and tunable structural and surface properties. Innovations such as cryogenic fabrication [276], protective coatings [275], thermal control [278], and multi-mode integration [277] have greatly expanded the performance, supporting their use in both laboratory-controlled and real-world environmental sensing applications.

### 3.5. GLAD-Based Electronic Nose Systems for Multi-Gas Sensing

In real-world environments, gases rarely appear in isolation; instead, they exist as complex mixtures composed of multiple analytes. This poses a challenge for conventional single-gas sensors, which often suffer from cross-sensitivity, limited selectivity, and difficulties in distinguishing overlapping spectral or electrical responses. As a solution, multi-gas sensing platforms, commonly referred to as electronic noses (e-noses), have emerged as powerful tools capable of detecting, identifying, and quantifying multiple gaseous species simultaneously. By mimicking the combinatorial detection approach of the mammalian olfactory system, e-noses offer a more holistic and adaptable sensing strategy. This makes them invaluable in applications such as air quality monitoring, industrial safety, medical diagnostics, and food freshness assessment.

In recent years, several research groups have explored the use of GLAD-fabricated metal oxide nanostructures in chemiresistive and hybrid gas sensors for electronic nose (e-nose) applications. As summarized in Table 8, a variety of metal oxides have been employed in GLAD-based e-noses, including WO_3_, SnO_2_, In_2_O_3_, NiO, CuO, and TiO_2_ [105,215,237,238,284,285,286]. These materials are typically deposited at θ ranging from 80° to 88°, resulting in the formation of villi-like or nanocolumnar morphologies. Reported film thicknesses generally fall between 200 and 400 nm, with porosities reaching up to 46.4% in some cases. Post-deposition annealing at temperatures between 200 and 550 °C is commonly performed to enhance crystallinity and optimize the sensing characteristics of the metal oxide films. 

**Figure 16:**
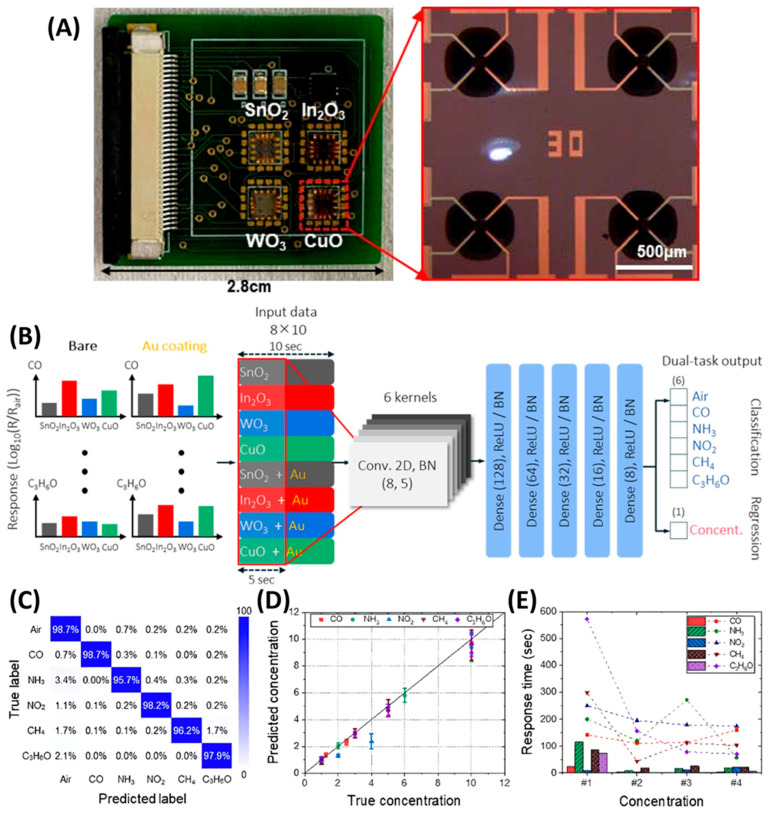
(**A**) Image of a 4 × 4 suspended microheater platform-based MOS gas sensor array integrated on PCB. (**B**) Architecture of the CNN model used for simultaneous classification of gas types and prediction of gas concentrations. (**C**) Confusion matrix of gas type classification results and (**D**) predicted gas concentrations (normalized from 0 to 10) obtained using the CNN on test data from an unseen gas sensor array. (**E**) Comparison of gas type identification times between the proposed CNN-based approach (bar graph) and conventional response time (scatter plot). Reproduced from [237] with permission from the American Chemical Society.

The integration strategies for these GLAD-based sensors vary across platforms but generally fall into three categories: (1) multi-sensor arrays, (2) single-sensor systems with thermal modulation, and (3) hybrid transistor-based devices. In the first category, Moon et al. [215,238] and Kang et al. [237] demonstrated the use of 3 × 3 and 4 × 4 sensor arrays, respectively, each comprising different GLAD-fabricated MOSs or functionalized nanostructures. For example, Figure 16A shows a photograph of a 4 × 4 gas sensor array by Kang et al. [237], where each chip (3.3 mm × 3.3 mm) integrates four microheater-based gas sensors, each coated with a different GLAD-fabricated metal oxide (SnO_2_, In_2_O_3_, WO_3_, and CuO). Notably, Kang et al. [237] developed a 16-channel array with four different oxide films deposited on a MEMS platform, achieving high batch uniformity with sensor-to-sensor resistance variation of less than ±15% at 250 °C. This design supported real-time multiplexed signal acquisition while maintaining low power consumption, with each sensor operating at just 11–15 mW. Alternatively, Lee et al. [105,286] introduced a minimalist single-sensor design using a GLAD-fabricated WO_3_ nanostructure on a thermally isolated anodic aluminum oxide (AAO) microheater platform. The sensor employed a staircase heating waveform and bridge-supported air-gap configuration to deliver rapid thermal response (reaching 300 °C in under 50 ms) with power consumption below 32 mW. This approach also provided long-term mechanical and thermal durability, surviving over 150,000 heating cycles and mechanical shocks equivalent to impact energies of 0.158 J [105]. A third, highly innovative architecture was developed by Ao et al. [285], who functionalized graphene field-effect transistors (GFETs) with GLAD-deposited TiO_2_ and SnO_2_ NRs. This dual-parameter sensing platform extracted both resistive and capacitive responses to gas exposure, allowing for six-gas classification with only two sensor elements. This design effectively combined the high sensitivity of nanostructured MOSs with the signal richness of transistor-based sensing, demonstrating enhanced performance with reduced system complexity.

GLAD-based e-nose systems have been evaluated across a wide spectrum of target gases. These include medically relevant biomarkers such as NO, NH_3_, H_2_S, and acetone, which are indicative of diseases like asthma, renal dysfunction, and diabetes [215,238,285]. In fire detection scenarios, Lee et al. [105] used GLAD-fabricated NiO and SnO_2_ nanocolumns to detect gases such as CO and HCl released during the early thermal decomposition of PVC, achieving a lead time of up to 91 s ahead of commercial smoke detectors. Kang et al. [237] and Ao et al. [285] targeted environmental pollutants like NO_2_, ethanol, methane, and formaldehyde, while Lee et al. [286] demonstrated the ability of a single GLAD sensor to discriminate between complex gas mixtures from culinary herbs with over 96% classification accuracy.

For signal analysis, earlier efforts relied on conventional machine learning techniques. Moon et al. [215,238] applied Principal Component Analysis (PCA) to differentiate gas types using normalized resistance values, capturing over 95% of data variance within the first two components. Ao et al. [285] used Linear Discriminant Analysis (LDA) on resistive and dielectric data to enhance gas separation accuracy in their dual-mode GFET sensors. In contrast, more recent studies have transitioned to deep learning models, particularly one-dimensional convolutional neural networks (1D-CNNs), for analyzing gas response time-series data. Figure 16B illustrates the architecture of the CNN designed by Kang et al. [237] for simultaneous gas classification and concentration prediction. The network uses 8 × 10 matrices as input, reflecting data from eight sensor channels over ten time points (from a 10-s moving window). The CNN consists of one convolutional layer (with six 8 × 5 kernels), followed by multiple fully connected layers (128 → 64 → 32 → 16 → 8), with batch normalization and ReLU activations. The output layer includes six softmax nodes for gas type classification and one node for gas concentration regression, enabling real-time multi-gas analysis. Figure 16C presents a confusion matrix summarizing the classification performance of the CNN on test data from a new sensor array. The system accurately identifies six gas classes (including air), achieving a 98.06% classification accuracy. Figure 16D shows the predicted gas concentration**s** (normalized 0–10) for each test sample. Despite variation in gas type and concentration, the average prediction error is only 10.15%, highlighting the robustness of the regression output. Figure 16E compares the minimum CNN-based prediction times for identifying gas types (bar chart) against traditional response times (scatter plot, defined as the time to reach 90% of the saturated response). The CNN achieves identification within 1–19 s for CO, NH_3_, NO_2_, CH_4_, and acetone (C_3_H_6_O), dramatically faster than conventional methods (44–174 s). Lee et al. [286] applied a similar CNN model to a single GLAD-WO_3_ sensor operated under pulsed heating conditions, obtaining 97.0% classification accuracy and MAPE values ranging from 13.7% to 19.8%. Importantly, CNNs consistently outperformed traditional classifiers such as support vector machines (SVMs), particularly in scenarios requiring real-time or early-stage detection [237].

The overall sensing performance of GLAD-based e-nose systems is impressive across key metrics. For example, NiO sensors fabricated by Lee et al. [105] achieved a ΔR response of 577 to PVC decomposition gases, while In_2_O_3_ sensors reported by Lee et al. [284] reached ΔR values of 331 for 5 ppm NO_2_. Moon et al. [215,238] achieved detection limits as low as 534 ppt for H_2_S, 899 ppt for NO, and 312 ppb for NH_3_, comfortably below thresholds relevant to medical diagnostics. Selectivity was significantly enhanced in Au-functionalized sensors, which showed minimal response to interfering gases like CO_2_ and acetone, even under 80% relative humidity [238]. Power consumption remained low across all GLAD-AAO platforms, with average values between 9 and 32 mW, and response times were generally under 10 s for most gases [284,286]. The robustness of GLAD sensors in high-humidity conditions further supports their suitability for breath-based diagnostics and indoor air quality monitoring [215,237,238].

In parallel, recent advances in micro-light-emitting diode (μLED)-integrated gas sensors have opened new avenues for ultra-compact, energy-efficient, and intelligent e-nose systems [239,240,243]. These sensors operate at room temperature and rely on photo-activated surface reactions enabled by near-UV μLEDs (typically ~395 nm) to detect and differentiate gases with high selectivity and sensitivity. When combined with deep learning algorithms, μLED sensors can perform accurate, real-time classification and quantification of multiple gases using a single or dual-sensor configuration [239,240,243].

**Figure 17:**
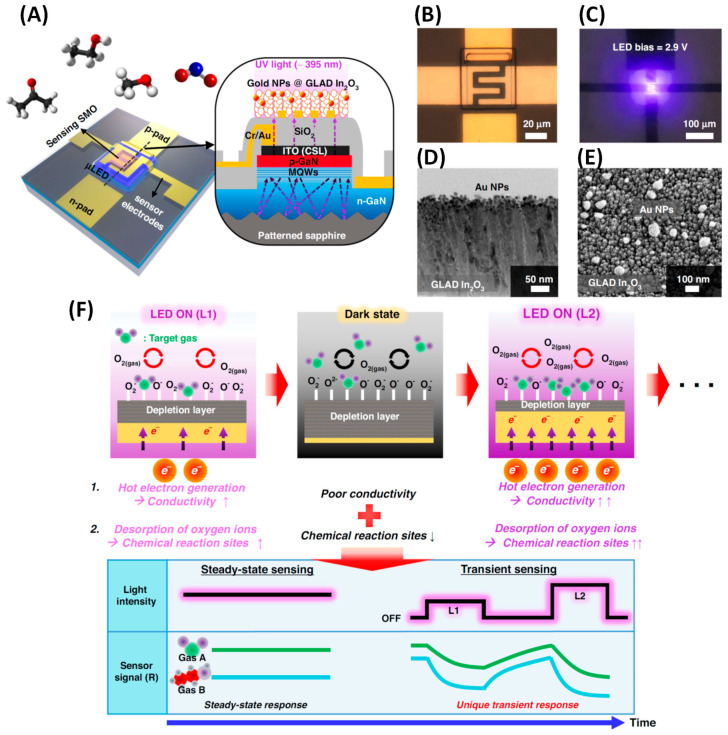
(**A**) Schematic illustration of the μLED-integrated photoactivated gas sensor (μLP). The inset shows the cross-sectional structure, highlighting the vertical stack of components. (**B**,**C**) Optical microscopy images of the fabricated sensor chip and the near-UV μLED (λ_peak_ = 395 nm) emitting under a forward bias of 2.9 V. (**D**,**E**) Cross-sectional TEM and top-view SEM images of the porous, columnar In_2_O_3_ sensing film coated with gold NPs, deposited via GLAD. (**F**) Conceptual diagram of the pulsed illumination strategy. By applying random high-intensity (L_2_) and low-intensity (L_1_) light pulses instead of steady illumination, gas-specific transient responses are enhanced, enabling selective detection of different gases using a single sensor. Reproduced from [240] with permission from Nature.

The operating principle of μLED gas sensors centers on optically triggered surface reactions (Figure 17). UV illumination generates photoexcited charge carriers in porous GLAD-deposited In_2_O_3_ sensing layers. To enhance sensitivity, plasmonic NPs (e.g., Ag or Au) are deposited atop the sensing layer. These NPs induce LSPR under UV illumination, generating energetic hot electrons that are transferred to the semiconductor surface, thus increasing the density of active charge carriers and accelerating gas-surface interactions. Different gases are identified by analyzing transient resistance responses generated during pseudorandom, time-varying UV illumination. Periodic modulation of the μLED intensity induces gas-specific surface reactions, as the photoactivation effects vary according to the redox nature of the target molecules. For instance, oxidizing gases such as NO_2_ typically extract electrons from the n-type In_2_O_3_, resulting in an increase in resistance. Conversely, reducing gases like ethanol, methanol, and acetone donate electrons to the conduction band, leading to a decrease in resistance. These interactions produce distinct temporal resistance patterns, effectively “dynamic fingerprints”, that characterize the presence and identity of specific gases. To extract and interpret these complex temporal features, the resistance signals are input into a CNN model. Trained on representative datasets, the CNN can accurately classify gas species and quantify their concentrations based on the unique time-domain characteristics of their response profiles. This enables real-time identification and quantification of multiple gases using only one or two μLED-integrated sensing elements, even in the presence of gas mixtures.

Structurally, μLED-based sensors use a vertically stacked, monolithic design where the UV light source lies beneath the GLAD-fabricated In_2_O_3_ sensing layer (~250 nm thick, ~35% porosity) [239,243]. Devices are fabricated using CMOS- and MEMS-compatible processes with patterned sapphire substrates, GaN μLEDs, SiO_2_ insulation, and gold electrodes.

**Figure 18:**
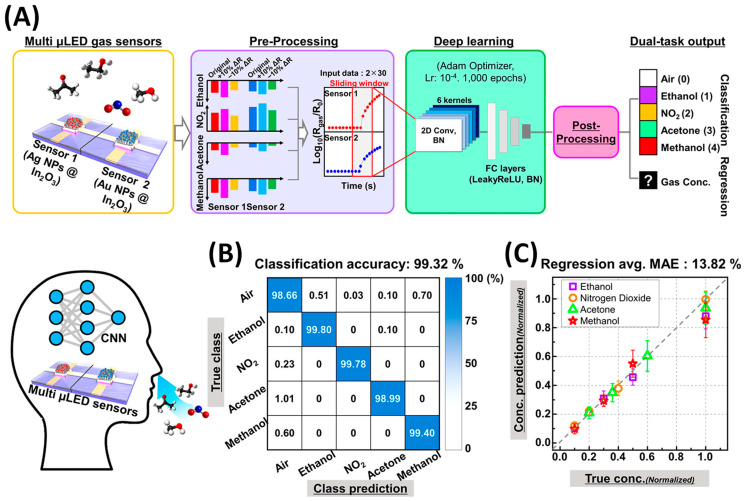
(**A**) Schematic of the electronic nose (e-nose) architecture combining two μLED-integrated gas sensors (Ag- and Au-decorated GLAD In_2_O_3_) with a CNN model for simultaneous classification and regression of five target gases (air, ethanol, NO_2_, acetone, and methanol). (**B**) Confusion matrix summarizing the gas type classification and (**C**) regression results for gas concentration prediction (normalized between 0 and 1). Reproduced from [243] with permission from the American Chemical Society.

The μLED sensors have been tested across various gas types and concentrations. Cho et al. [240] tested methanol, ethanol, acetone, and NO_2_ at multiple concentrations (10–100 ppm for VOCs; 0.1–1 ppm for NO_2_). Their CNN model achieved 96.99% classification accuracy and 31.99% MAPE in concentration prediction, with detection limits down to 10 ppm for alcohols and sub-ppm for NO_2_. In binary mixture detection, their system accurately resolved methanol–ethanol mixtures, achieving over 97% classification confidence and ~34% MAPE in concentration estimation. Lee et al. [239,243] used two μLED sensors (Ag- and Au-decorated) to classify air, NO_2_, ethanol, acetone, and methanol. The system achieved 99.5% classification accuracy and 12.8% regression error, while operating at just 0.38 mW, making it more than 100× more energy efficient than conventional heater-based sensors. Figure 18A illustrates the architecture of the ultra-low-power e-nose system, which integrates two micro-LED (μLED) gas sensors, decorated with Ag and Au nanoparticles, with a CNN model. This setup enables simultaneous classification and quantification of five gases: air, ethanol, NO_2_, acetone, and methanol. Figure 18B presents the confusion matrix of the CNN-based gas classification results, demonstrating excellent performance with a total accuracy of 99.32%, confirming the strong discriminative capability of the system across multiple gases. Figure 18C shows the regression results for gas concentration prediction, normalized between 0 and 1. The system achieves a mean absolute error (MAE) of 13.82%, highlighting its effectiveness in not only identifying but also quantifying gas concentrations using minimal sensor input. Gas-specific transient features were recognizable within 10–30 s of exposure, enabling real-time detection [239,240,243].

μLED-based gas sensors offer several key advantages. They operate efficiently at room temperature, eliminating the need for power-hungry heaters and reducing ignition risks for flammable gases. Their ultra-low power consumption (<0.4 mW total), miniaturized form factor, and CMOS compatibility make them ideal for integration into portable devices, wearables, and IoT platforms. Furthermore, by using deep learning to analyze dynamic sensor responses, they overcome the long-standing issue of poor selectivity in traditional metal oxide gas sensors. This combination of intelligent sensing, energy efficiency, and scalability positions μLED-based e-nose systems as a promising solution for next-generation environmental sensing, industrial safety, and health diagnostics.

GLAD enables the fabrication of highly porous, nanostructured metal oxide films with dramatically increased surface area, which in turn facilitates ultra-low detection limits, often reaching the ppb or even ppt range (see Table 5 and Table 8). As highlighted in Table 8, GLAD-based electronic nose (e-nose) systems have achieved detection limits as low as ~0.5 ppb for H_2_S [215], demonstrating exceptional sensitivity and selectivity. In contrast, conventional e-nose platforms typically exhibit detection thresholds in the 0.1–10 ppm range, making GLAD-based architectures orders of magnitude more sensitive and thereby significantly enhancing trace gas discrimination accuracy.

From a fabrication standpoint, GLAD is a cost-efficient physical vapor deposition (PVD) technique that does not require lithographic masks, cleanroom facilities, or high-temperature reactions. Instead, it leverages high-angle deposition (e.g., RF sputtering or e-beam evaporation) to create nanocolumnar films in a single step. A single GLAD run can deposit sensor layers across an entire wafer, enabling batch fabrication of dozens of devices in parallel. For example, the e-nose developed by Cho et al. [240] employed a ~268 nm porous In_2_O_3_ film deposited via GLAD in approximately 90 min, uniformly coating multiple sensor sites. Because it bypasses multi-step processes like photolithography or screen printing, GLAD substantially reduces both per-unit fabrication time and capital costs.

In terms of system robustness, GLAD-based e-noses have demonstrated strong mechanical and environmental durability. For instance, Au-decorated GLAD sensors maintained stable selectivity even under high humidity conditions (up to 80% RH) [238]. A WO_3_-based GLAD sensor built on a thermally isolated anodic aluminum oxide (AAO) microheater survived over 150,000 heating cycles and mechanical shocks equivalent to an impact energy of ~0.158 J without failure [105]. These platforms also offer low power consumption (typically 9–32 mW per microheater) and rapid response/recovery times under 10 s for most gases [243]. Collectively, these attributes underscore the potential of GLAD-fabricated e-noses to deliver sensitive, reliable, and scalable performance, often surpassing conventional designs in both efficiency and robustness.

## 4. Emerging Strategies for Enhancing GLAD-Based Gas Sensors

While GLAD enables the fabrication of highly porous, anisotropic nanostructures with tunable geometry and composition, further performance enhancements require chemical and physical modifications that improve surface reactivity, selectivity, and signal transduction. When integrated with the structural features of GLAD, these functionalization strategies enable gas sensors to achieve lower detection limits, faster response times, and improved discrimination against interferents. This section categorizes enhancement approaches into four major strategies: (1) doping and heterojunction formation, (2) nanoparticle decoration, (3) surface functionalization with MOFs, polymers, or SAMs, and (4) integration of photoactive and quantum materials. Each strategy is discussed in terms of its mechanism, GLAD compatibility, and exemplary applications. A consolidated summary of these strategies is provided in Table 3, and selected implementations are highlighted in Table 4.

### 4.1. Hybrid Nanostructures and Composite Architectures

Despite the extraordinary versatility of GLAD in controlling nanostructure geometry, composition, and layering, most current GLAD-based gas sensors continue to employ relatively simple architectures, such as slanted columns, zigzags, and helices (Figure 3A–E), often enhanced with surface-decorated NPs (Figure 3H,I). While these conventional designs provide reasonable sensitivity and are straightforward to fabricate, they only scratch the surface of what GLAD can offer. GLAD is uniquely capable of producing complex hybrid and composite nanostructures that can drastically improve sensor performance. For instance, multilayered NRs with alternating topologies (Figure 3H) can combine slanted, vertical, and helical layers to balance gas diffusion, light scattering, and electrical connectivity. These structures also offer opportunities for integrating built-in electrodes or photonic enhancements. Multisegmented heterorods (Figure 3G), created by sequentially stacking different materials along a single NR, allow for vertical p–n junctions and segment-specific interactions for multi-analyte detection or optoelectronic coupling. Core–shell NRs (Figure 3J) provide functional separation between the sensing core (e.g., metal oxide) and a protective or catalytic shell (e.g., polymer or metal), enabling enhanced stability, response speed, and selectivity. Janus NRs and checkerboard helices (Figure 3K,L) introduce compositional asymmetry, enabling anisotropic sensing responses and directional flow detection. In addition, doped nanostructures (Figure 3N,O) allow tunable charge transport and surface chemistry via dopant atoms or embedded nanoparticles formed through post-deposition annealing. These embedded heterostructures offer increased interfacial area and promote more stable gas-surface interactions than conventional surface-decorated structures. GLAD also supports the fabrication of porous NRs through composite-and-etching methods, where a removable metal (e.g., Cu or Ag) is co-deposited with a stable host matrix, then selectively etched to form well-defined internal porosity [287,288]. Finally, composition-graded NRs can be synthesized by dynamically varying the deposition ratio of two materials during growth, creating spatial gradients in chemical composition, gas reactivity, or nanoparticle distribution along the NR axis [289].

Collectively, these advanced architectures, illustrated in Figure 3G–O, represent a vast and largely untapped design space within GLAD-based gas sensing. Although individual structures have demonstrated promising results, comprehensive design rules for selecting and integrating these configurations remain underdeveloped. To fully realize the potential of GLAD, future work must establish systematic frameworks for correlating structure, composition, and function. Doing so will enable the rational design of high-performance, multifunctional gas sensors tailored to complex real-world environments [52,53].

### 4.2. Functional Coatings and Selectivity Enhancement

While GLAD excels in producing porous, anisotropic nanostructures, its integration with complementary fabrication techniques unlocks new structural and functional possibilities that extend far beyond what GLAD alone can achieve. These hybrid strategies help overcome the inherent limitations of GLAD, such as material constraints, conformal coating challenges, and resolution limits, while enabling multifunctionality and fine-tuned surface control. This section highlights key hybridization pathways and their roles in advancing the design of GLAD-based gas sensors.

- *Atomic layer deposition.* ALD provides conformal, sub-nanometer-thick coatings that are ideal for modifying high-aspect-ratio GLAD nanorods (e.g., Figure 3N). When deposited onto GLAD scaffolds, ALD layers can form core–shell structures that introduce catalytic activity (e.g., Pt or Pd), passivate surface defects, or tune band alignment. For example, ALD-coated ITO GLAD NRs with a Pt shell exhibit improved electrochemical sensor performance [290]. The uniformity of ALD is particularly beneficial for densely packed NR arrays where precise surface modification is required throughout the full depth of the structure.
- *Chemical vapor deposition (CVD).* CVD enables the growth of high-quality crystalline films, including 2D materials like graphene or MoS_2_. When applied to GLAD nanostructures, CVD-derived coatings can significantly improve charge transport and gas adsorption through synergistic interfacial interactions. For instance, GLAD NRs coated with graphene layers can support hybrid electrical–chemical sensing and multifunctional detection schemes involving both electronic and optical readouts. These hybrid architectures combine the vertical access channels provided by GLAD with the conductivity and surface chemistry of 2D materials.
- *Plasma etching.* Plasma processing serves as a versatile post-GLAD modification tool to tailor surface roughness, porosity, and chemistry. Oxygen plasma, for example, can introduce hydrophilic functional groups that improve polar gas adsorption. Selective etching of NR sidewalls or tips can reveal buried catalytic zones or create hierarchical porosity, which enhances gas diffusion and reduces response time. These effects are particularly useful for tuning sensing kinetics and surface specificity in dense GLAD arrays.
- *Template-Assisted Fabrication.* Combining GLAD with pre-patterned templates or nanosphere masks (e.g., nanosphere lithography (NSL)) enables precise spatial control over nanostructure geometry and placement [291]. This approach can produce nanohole arrays, nanocaps, or curved architectures with improved uniformity and spatial resolution. For example, recent studies have shown that combining GLAD and NSL significantly enhances hydrogen sensor performance [292,293]. Specifically, Pd_80_Co_20_ nanohole arrays demonstrated ultrafast response times (~1 s), part-per-billion detection limits, and excellent selectivity and stability, especially when coated with PMMA to exclude interfering gases and moisture [292]. Likewise, Pd_67_Co_33_ nanocap arrays demonstrated magneto-optical sensing with sub-second response times in high-humidity environments when paired with polymer barriers [293].

### 4.3. Integration with Low-Dimensional and Soft Materials

Incorporating functional nanomaterials into GLAD-fabricated architecture offers a powerful route to enhance gas sensor performance by introducing additional chemical specificity, electrical tunability, and multimodal sensing capabilities. While the incorporation of metal nanoparticles and quantum dots has been explored (Section 3.2), other low-dimensional and soft materials, including 2D materials, polymers, MOFs, and SAMs, remain underutilized. The vertically aligned, porous morphology produced by GLAD provides an ideal scaffold for integrating these materials, enabling hybrid systems that combine the spatial control and anisotropy of GLAD with the chemical richness of functional nanomaterials.

- *2D Materials*. Layered materials such as graphene, MoS_2_, WS_2_, and MXenes can be integrated with GLAD NRs to enhance conductivity, introduce selective adsorption layers, or enable electron/hole transfer at the interface. For instance, MoS_2_-coated GLAD structures could show improved NO_2_ and NH_3_ sensing due to enhanced charge transport and selective adsorption. MXenes, with their metallic conductivity and surface terminations, can contribute hydrophilic or polar sensitivity while conformally covering complex GLAD morphologies without compromising porosity or anisotropy.
- *Functional and Conducting Polymers.* Polymers offer excellent chemical tunability and compatibility with GLAD substrates. Functional polymers, such as PMMA or polyacrylic acid, can serve as molecular sieves or humidity barriers, improving selectivity for target gases. Conducting polymers, including polyaniline (PANI), polypyrrole (PPy), and PEDOT:PSS, can introduce alternative transduction pathways, including conductivity changes due to doping or swelling, thereby enhancing signal diversity. These materials can be uniformly applied via spin-coating, spray deposition, or vapor processes and maintain performance under mechanical or thermal stress, making them well-suited for flexible or wearable sensing platforms.
- *Metal–Organic Frameworks.* MOFs, such as ZIF-8 and MIL-101, are crystalline, nanoporous materials that can offer precise molecular sieving and chemical specificity. When coated onto or infiltrated into GLAD structures, MOFs can create selective gas diffusion paths and enhance sensitivity by concentrating trace analytes like VOCs or ammonia. MOFs can also reduce humidity interference and support modular sensing through post-synthetic functionalization.
- *Self-Assembled Monolayers.* SAMs offer molecular-level control of surface chemistry, enabling the introduction of functional groups (–NH_2_, –COOH, –SH) for selective gas interaction or surface passivation. Applied to GLAD NRs, SAMs enhance stability, reduce fouling, and tailor wettability. Selective patterning of SAMs on sensor arrays also enables spatially distinct response profiles, supporting multi-analyte detection.

### 4.4. Device Engineering: Electrode Configuration and Signal Readout

#### 4.4.1. Electrode Configuration and Conductance Anisotropy

**Figure 19:**
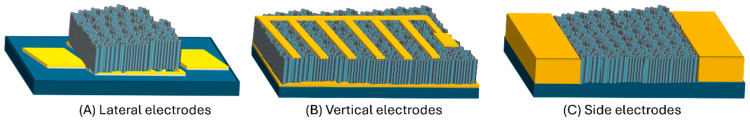
Electrode configurations for resistive gas sensors based on GLAD nanostructures, showing (**A**) vertical, (**B**) lateral, and (**C**) side-contact configurations.

The performance of resistive gas sensors based on GLAD nanostructures is strongly influenced by the configuration of the electrodes, which determines current pathways, sensing volume, and gas accessibility. Due to the directional growth and porosity of GLAD films, electrode geometry must be carefully matched with the structural anisotropy of the NRs. Three main configurations are commonly used, as illustrated in Figure 19.

Lateral configuration (Figure 19A), the most widely adopted in GLAD-based sensors (see Section 3.1), uses planar electrodes patterned on the substrate to facilitate in-plane conduction. This geometry allows for unobstructed gas access but requires sufficient lateral connectivity across tilted or disconnected NRs. Vertical configuration (Figure 19B) places electrodes above and below the GLAD layer. It offers compact integration and high sensitivity when vertical conductivity is ensured, but risks obstructing gas diffusion unless transparent or patterned top electrodes (e.g., ITO meshes) are employed. Side-contact configuration (Figure 19C) positions electrodes on opposite sides of a trench or bridge, allowing unrestricted three-dimensional gas exposure and potential integration with optical sensing modes. This configuration offers high performance potential but is fabrication-intensive and has not yet been widely explored.

Table 9 summarizes the requirements and trade-offs of these configurations. For example, vertical configurations require interconnected rods or conductive coatings, lateral configurations depend on tilt alignment and rod bridging, and side-contact configurations benefit from dense, mechanically robust films.

#### 4.4.2. Well-Separated NR Array Sensors

In well-separated vertical NR arrays, especially those using vertical or lateral electrode configurations, gas sensing performance is largely governed by the properties of individual NRs or representative unit cells, similar to single-NW sensors [294,295,296]. Key NR parameters such as diameter, length, crystallinity, surface morphology, doping, and electrical contact type critically influence sensitivity, response time, selectivity, and stability. These attributes can be systematically tailored through GLAD and post-deposition modifications.

*Crystallinity and structural quality.* Crystallinity plays a central role in balancing charge transport efficiency and surface reactivity [297,298]. Single-crystalline NRs enable rapid electron transport and high stability due to their defect-free structure, although they may exhibit reduced sensitivity owing to fewer adsorption sites. In contrast, polycrystalline NRs offer enhanced sensitivity via grain boundary adsorption at the expense of slower carrier mobility and potential signal drift. GLAD provides control over crystallinity primarily through substrate temperature, material selection, and annealing, with deposition angle playing a secondary role.

*Nanorod diameter.* Diameter has a strong influence on depletion behavior [295,299]. When the NR diameter approaches or falls below the Debye length (~≤50 nm), complete charge depletion upon gas adsorption leads to a significant boost in sensitivity. However, these ultra-thin rods may suffer from high resistance and limited charge carriers. GLAD enables precise diameter control through vapor angle, substrate rotation, and deposition duration. To mitigate conductivity loss, conformal coatings (e.g., ALD with Pt or Pd) or core–shell architectures can be employed.

*Nanorod Length.* NR length impacts both the conduction path and the available surface area for gas interaction. Short rods (≤5 µm) favor rapid response due to reduced charge transport distance, while long rods (>10 µm) offer greater sensitivity but slower kinetics. Advanced GLAD designs, including multilayered or segmented structures, can combine short-path conduction with large surface area. Heterorod architectures, featuring vertically stacked dissimilar materials, allow the creation of built-in p–n junctions for improved selectivity.

*Surface Roughness and Porosity:* Surface texture enhances gas adsorption by increasing the number of active sites [300,301]. Moderate roughness boosts sensitivity and response speed, but excessive roughness may introduce trap states and impede recovery. GLAD naturally produces rough, high-aspect-ratio surfaces, which can be fine-tuned via plasma etching or thermal treatment. Porous nanorods fabricated through sacrificial-phase co-deposition and etching further improve adsorption kinetics and increase effective surface area.

*Doping Concentration and Composition Gradients.* Doping concentration governs baseline conductivity and sensor responsiveness [302]. Lower doping yields higher sensitivity but slower response times, whereas higher doping favors speed at the expense of sensitivity. GLAD enables compositional tuning through co-deposition and annealing. Notably, graded-doping profiles, where composition varies along the NR, allow spatially modulated reactivity and charge transport, enabling multifunctional sensing behavior.

*Electrical Contact Configuration.* The type of electrical contact, Ohmic or Schottky, has a substantial effect on sensor performance [303,304]. Schottky barriers can be modulated by gas adsorption, amplifying sensitivity. GLAD enables the fabrication of Janus NRs with asymmetric compositions, offering a pathway to engineer directional charge transport and dynamically tunable barrier heights for Schottky contact. In contrast, for Ohmic contacts, performance improvements focus on minimizing contact resistance and improving stability. This can be achieved through uniform nanorod alignment and the incorporation of conductive polymers such as PEDOT:PSS.

#### 4.4.3. Porous Thin Film Sensors

Porous nanostructured thin films represent a key class of GLAD-based gas sensors due to their tunable surface area, pore architecture, material composition, and structural connectivity. Their performance depends on a delicate balance among these parameters, which together determine sensitivity, response time, selectivity, and environmental stability.

*Surface area and porosity.* High surface area increases active adsorption sites, improving sensitivity. Optimal porosity facilitates gas diffusion; however, excessive porosity can compromise mechanical robustness and disrupt charge transport networks, raising resistance and reducing stability. Composite–etching strategies using sacrificial metals (e.g., Cu, Ag) enable the formation of porous yet mechanically resilient films [287,288]. Post-deposition plasma etching can further fine-tune surface roughness and porosity for enhanced gas accessibility without structural degradation.

*Pore Size and Distribution.* Pore size critically impacts gas transport and adsorption behavior. Mesopores (<50 nm) enhance surface interaction but may slow diffusion, while macropores (>50 nm) facilitate rapid transport but reduce surface density. Hierarchical structures combining micro-, meso-, and macropores effectively balance these trade-offs, achievable through multilayer GLAD growth or porous rod assembly. Template-assisted GLAD, including NSL, offers precise control over pore size and spatial distribution for uniform gas exposure.

*Material composition and defect engineering.* Film composition and defect density dictate chemical reactivity and electronic properties. Noble metal doping (e.g., Pt, Pd, Au) can enhance catalytic activity, while engineered oxygen vacancies can improve adsorption and charge transfer. These features can be tuned during GLAD growth or by post-processing techniques such as ALD. Composition-graded structures further enable spatially resolved chemical functionality in films where uniform doping is difficult to achieve.

*Charge transport and structural connectivity*. Maintaining efficient charge percolation in porous films is critical yet challenging. Disconnected or poorly connected structures elevate resistance and degrade signal quality. Strategies to improve connectivity include multilayered heterostructures, embedded conductive nanoparticles, and conformal ALD coatings that bridge nanodomains without blocking gas flow. Core–shell architectures can also offer continuous conduction pathways while preserving porosity.

*Thermal management and operating conditions.* Operating temperature affects both kinetics and selectivity. Elevated temperatures accelerate desorption and shorten response times but reduce specificity due to nonspecific adsorption. Low-temperature operation enhances selectivity but may require UV activation or catalytic additives. Integration of reactive 2D materials (e.g., MoS_2_, MXenes) and MOFs introduces selective adsorption sites that support efficient performance under low-power and ambient-temperature conditions. Thermal stabilization coatings, applied via ALD or CVD, help extend the usable temperature range.

*Environmental Stability and Humidity Tolerance*. Humidity poses a significant challenge by competing for adsorption sites and inducing signal drift. Hydrophobic surface modifications, such as ALD-grown oxides, SAM coatings, or MOF layers, mitigate moisture interference by repelling water molecules or functioning as molecular sieves. These strategies improve reliability in variable environments and extend sensor lifespan.

*Impact of electrode configuration on resistive gas sensors.* Electrode geometry and material are vital to resistive sensor performance. Interdigitated electrodes (IDEs) offer broad area coverage, high sensitivity, and compact layout, especially effective in porous films. However, non-uniform film morphology can lead to uneven current distribution. End-contact (two-terminal) designs are simpler and suited for single NW sensors, while four-probe setups provide precision by eliminating contact resistance, though they are impractical for scalable sensing. Electrode material selection also matters. Ohmic contacts (e.g., Au, Pt, Ti) provide linear and stable conduction, ideal for long-term reliability. Schottky contacts (e.g., Pt/ZnO) introduce rectifying barriers that are sensitive to gas-induced modulation, enhancing sensitivity at the cost of nonlinearity. Electrode spacing influences the electric field and power requirements, with tighter spacing increasing local fields but potentially introducing noise. Alignment between electrode layout and nanostructure orientation is crucial for uniform response. The electrode–film interface further determines response speed and stability. High-resistance interfaces degrade signal integrity, whereas nanostructured or polymer-modified electrodes reduce interfacial impedance, improving signal transduction and recovery time.

### 4.5. Expanding the Modalities of GLAD-Fabricated Nanostructures for Optical Gas Sensing

While most GLAD-based optical gas sensors have focused on optical absorption and SERS, the morphological versatility of GLAD nanostructures also makes them well-suited for a broader set of underexplored optical sensing modalities. These include LSPR, fiber-optic sensing, fluorescence enhancement, photoacoustic spectroscopy (PAS), and colorimetric detection. The anisotropy, porosity, and tunable architecture of GLAD-fabricated films offer rich opportunities to enhance light–matter interactions across these modalities, enabling new directions in gas sensor development.

#### 4.5.1. LSPR: Unlocking Plasmonic Sensitivity Through Tailored Nanostructures

LSPR provides real-time, label-free detection by monitoring extinction peak shifts in response to refractive index changes upon gas adsorption [305,306]. Unlike SERS, LSPR is ideal for small molecules and supports rapid response with minimal sample preparation. GLAD enables the fabrication of nanostructures such as nano-islands, slanted columns, and patterned arrays with controlled spacing and anisotropy that match the functional requirements of LSPR-based sensing. Despite its potential, LSPR remains underutilized in GLAD-enabled gas sensing, although its application has been well-demonstrated in biosensing [307,308]. Structures including slanted or vertical nanocolumns [309,310,311,312], nanosphere-templated patches [313], nano-protrusions atop plasmonic gratings [314], and Au/TiO_2_/Au multilayer sandwiches [311] have enabled detection of analytes such as antibodies, endotoxins, and nucleic acids [309,310,314,315]. Extending these designs to gas-phase sensing could yield compact, scalable platforms with tailored plasmonic responses. To advance LSPR-based gas sensors, future research should focus on GLAD architecture optimization, surface functionalization for volatile species, and dynamic platforms such as flow-through cells or thermally responsive substrates.

#### 4.5.2. Fiber-Integrated GLAD Sensors: Toward Distributed, Multimodal Gas Detection

Fiber-optic gas sensors offer several advantages, such as miniaturization, electrical isolation, and remote deployment [316,317]. However, traditional configurations (e.g., Fabry-Pérot cavities, Bragg gratings) are limited by short optical interaction lengths. GLAD overcomes this limitation by enabling nanostructure deposition onto curved surfaces, enhancing evanescent coupling and LSPR/SERS field confinement. For example, the first demonstration of GLAD nanorod deposition onto fiber substrates was reported by Fan et al. [318] in 2005. Subsequent studies have demonstrated trace-level gas detection using SERS on GLAD-coated fiber tips and tapers [319] as well as SPR on GLAD-integrated fibers [320,321]. These approaches support multimodal detection with tunable response characteristics. As fabrication methods mature, integrating GLAD nanostructures into distributed fiber networks could enable high-resolution gas mapping and in situ environmental monitoring.

#### 4.5.3. Fluorescence Amplification via GLAD: Enhancing Emission for Optical Readout

GLAD nanostructures can significantly enhance fluorescence signals from dye-functionalized sensors by increasing surface area and coupling to LSPR fields [322,323]. Metallic NRs, such as vertical Ag NRs fabricated via GLAD, have achieved fluorescence enhancement factors of up to 200× [324]. Structuring these arrays atop microposts further improves signal-to-noise ratios and reduces nonspecific interactions [325]. Beyond metals, dielectric NRd arrays grown on photonic crystals via GLAD have yielded 114× enhancement through guided-mode resonance and 3D porous excitation volumes [326]. These hybrid structures are ideal for detecting gas-sensitive fluorophores through emission intensity, spectral shift, or lifetime changes. Demonstrations in biochip applications highlight the scalability of GLAD for such platforms [327], which could be adapted for gas sensing with visible, real-time readouts.

#### 4.5.4. GLAD-Enabled Photoacoustics: Amplifying Acoustic Signals Through Optical Engineering

PAS offers ultra-sensitive gas detection by converting absorbed light into acoustic waves [327,328]. GLAD nanostructures, especially porous Fabry-Pérot interferometers (FPIs), can enhance both optical coupling and acoustic generation [329,330,331]. P. Hajireza et al. [330] demonstrated in vivo reflection-mode optical-resolution photoacoustic microscopy using GLAD nanostructured FB interferometers. These GLAD-based PAS systems exhibit near-field optical confinement and efficient acoustic conversion, enabling dynamic sensing across variable angles and gas species. Further integration of birefringent GLAD films or thermally responsive layers could enable real-time, selective PAS sensors for field applications.

#### 4.5.5. Structural Color Sensing: GLAD-Based Colorimetric Platforms for Visual Detection

Colorimetric sensors exploit structural color changes in response to gas-induced optical shifts, offering label-free, power-free detection [332]. GLAD nanostructures inherently produce interference and diffraction effects through columnar or multilayered geometries, making them excellent candidates for visually interpretable gas sensors. GLAD-grown materials, such as TiO_2_, Ni/NiO, and porous Ge, have been used to fabricate colorimetric structures like Bragg stacks, nanozyme films, and Gires-Tournois resonators [333,334,335,336]. These systems have demonstrated colorimetric responses to humidity, biomolecules, and viruses via chromatic and refractive index shifts. Applying similar designs to gas sensing could yield low-cost, portable platforms for real-time, visual gas monitoring in the field.

#### 4.5.6. Toward Rational Design: Bridging Nanostructure Morphology and Optical Function via Modeling and AI

Although GLAD nanostructures have demonstrated enhanced signal output across many optical modalities, the relationships between fabrication parameters, morphology, and optical performance remain largely empirical. Parameters such as porosity, NR tilt, and inter-rod spacing influence LSPR, SERS, fluorescence, and cavity resonance. However, robust design rules remain underdeveloped. Bridging this gap will require a dual strategy that combines systematic experimentation with advanced modeling. High-throughput GLAD fabrication, coupled with in situ optical characterization and imaging techniques (e.g., SEM, AFM, ellipsometry), can generate detailed datasets linking nanostructure morphology to optical properties. Meanwhile, numerical tools such as FDTD, DDA, RCWA, and multiphysics simulations can reveal subwavelength light–matter interactions within complex GLAD geometries. AI integration can further accelerate this discovery process [337]. Machine learning models trained on morphological and optical data can predict performance metrics like LSPR shifts or enhancement factors. Generative AI and reinforcement learning can propose novel architectures tailored for gas-specific sensing requirements. Physics-informed neural networks (PINNs) offer a hybrid approach, embedding physical laws into AI models to yield interpretable and high-accuracy predictions. Closed-loop, AI-driven fabrication, where deposition parameters are dynamically adjusted based on sensor feedback, could transform GLAD into a precision manufacturing platform. Ultimately, this synergy between AI, modeling, and fabrication could enable design maps that correlate GLAD conditions with performance, streamlining sensor development and unlocking the full potential of GLAD-based optical gas sensing.

## 5. Conclusions

GLAD has emerged as a uniquely powerful technique in the development of next-generation gas sensors, offering structural tunability, high surface area, and compatibility with a wide range of materials. This review has comprehensively examined how GLAD can be harnessed to create advanced nanostructured platforms ranging from vertically aligned rods to complex multilayered and composite architectures that significantly enhance gas sensor performance. The morphological flexibility of GLAD allows precise tuning of porosity, surface area, and nanorod topology, directly impacting gas adsorption and diffusion kinetics. When combined with composition engineering, such as doping and heterostructure formation, GLAD enables tailored electronic and chemical properties for improved sensitivity and selectivity. Moreover, the integration of GLAD nanostructures with catalytic nanoparticles, quantum dots, conducting polymers, MOFs, and SAMs introduces multi-functional sensing pathways, broadening detection capabilities across gas species and environmental conditions. Recent innovations, including porous nanorod designs, composition-graded structures, and template-assisted patterning, demonstrate the evolving role of GLAD in high-resolution sensor architectures. Hybrid fabrication techniques, such as GLAD combined with ALD, CVD, or NSL, further expand their versatility and device integration potential. These developments set the stage for GLAD-based sensors to meet the growing demands for flexible, low-power, and intelligent sensing systems.

Despite its advantages, GLAD-based gas sensors still face several challenges. These include improving long-term stability, achieving better environmental tolerance, and integrating with modern electronic platforms for real-time monitoring. Additionally, a deeper understanding of structure-function relationships, quantitative sensing mechanisms, and device-level design rules remains critical. Bridging these gaps will require a multidisciplinary approach that incorporates computational modeling, machine learning, and high-throughput experimental design.

Looking forward, GLAD offers a promising route for scalable and cost-effective fabrication of multifunctional gas sensors. With continued research into complex nanostructure engineering, hybrid material integration, and sensor-system-level optimization, GLAD-based platforms are well-positioned to play a key role in future smart sensing technologies for environmental monitoring, healthcare, industrial safety, and beyond.

## Figures and Tables

**Table 1:** Comparison of PVD techniques for GLAD nanostructure fabrication.

PVD Technique	Advantages	Disadvantages	Material Compatibility	Effect on GLAD Nanostructures
**Thermal Evaporation [32]**	- Simple and low-cost setup - High purity films - Good for volatile materials	- Poor control of deposition energy	Metals, organics, some oxides (via reactive evaporation)	- High porosity - Less dense columns - Often amorphous as deposited - Requires post-annealing for crystallinity
**E-beam Evaporation [32]**	- Enables deposition of high-melting-point materials - Good directionality	- Higher cost than thermal evaporation - Potential beam-induced damage	Refractory metals, oxides, semiconductors	- Villi-like or nanocolumn structures with moderate porosity - Better stoichiometry than thermal evaporation - Crystallinity depends on substrate heating
**IBAD [36]**	- Independent control of film growth and ion flux - Enhances film density and orientation	- Complex equipment - Slower deposition rate	Metals, oxides, nitrides	- Tailorable column tilt and density - Lower porosity - Higher crystallinity possible - Smooth, aligned NRs with enhanced mechanical integrity
**RF/DC Sputtering [36]**	- Wide material range - Scalable - High energy species for dense growth	- May lead to substrate heating - Resputtering issues for complex targets	Metals, metal oxides, nitrides, alloys	- Denser nanostructures with finer control over morphology - Moderate to low porosity - Enhanced column uniformity - Improved crystallinity with energy input
**Reactive Sputtering [106]**	- Good for nitrides and oxides	- Requires gas flow control - May have reduced deposition rate	Oxides, nitrides, fluorides	- Moderate porosity - Can achieve specific stoichiometry with controlled reactive gas flow
**PLD [36]**	- Stoichiometric transfer from complex targets - High deposition rate for small areas	- Small area deposition - Expensive setup	Complex oxides, multicomponent materials	- Good composition control - Morphology depends on background gas and laser parameters - Moderate porosity - Crystallinity tunable via substrate temperature

**Table 3:** Strategies to improve the performance of gas sensors. ↑ indicates an increase; ↓ indicates a decrease.

Strategy	Mechanism	Key Benefit
Heterostructures (p–n, n–n)	Band bending, charge depletion	↑ Selectivity, ↑ Response
Core–Shell Structures	Shell controls gas access & charge modulation	↑ Sensitivity, optimized depletion layer
Doping	Carrier modulation, oxygen vacancies	↑ Active sites, ↓ Operating temp.
Noble Metal Decoration	Schottky barrier, spillover catalysis	↑ Sensitivity, ↑ Stability
MOS NP Decoration	p–n junctions, charge modulation	↑ Selectivity
Quantum Dot Functionalization	Light harvesting, surface sensitization	Room-temp sensing, ↑ Sensitivity
SAMs/Chemical Grafting	Functional group–gas interaction	Molecular specificity
MOF Coating	Molecular sieving, pre-concentration	↑ Selectivity, ↓ Cross-interference

**Table 4:** Potential coating methods for MOFs and polymers on GLAD nanostructures.

Coating Type	Method	Advantages	Potential Problems
**MOF**	In situ solvothermal/hydrothermal growth [175]	Strong adhesion; uniform crystal growth; conformal coating	Requires high temperature; may damage delicate nanostructures
	Dip-coating/spin-coating [176]	Simple, low-cost; scalable	Non-uniform coverage; weak adhesion; film cracking after drying
	Interfacial layer-assisted growth [177]	Improved nucleation and adhesion via functional layer (e.g., APTES)	Additional processing step; limited MOF-substrate compatibility
	Spray coating/electro-spraying [178,179]	Good control over film thickness and coverage; scalable	May require multiple passes; lower crystallinity
	Solvent-free CVD-like growth [180]	Thin, conformal coatings without solvent exposure	Limited to volatile precursors; MOF diversity restricted
**Polymer**	Layer-by-layer (LbL) deposition [181]	Nanometer-scale thickness control; high versatility for chemical tuning	Time-consuming; may require many layers for adequate functionality
	Initiated chemical vapor deposition (iCVD) [182]	Conformal coating; solvent-free; excellent for porous structures	Requires vacuum system; limited monomer choices
	Plasma polymerization [183]	Conformal and uniform coating; customizable surface chemistry	Surface damage possible; can alter GLAD morphology
	Dip-coating/spin-coating [184]	Simple and fast; compatible with many polymers	May bridge or clog porous structures; film uniformity may vary

**Table 8:** Summary of GLAD-based electronic nose.

Sensor Architecture	Method/Accuracy	Mat	Structure	Deposition Condition	Target Gases (LOD)	Response and Sensitivity	t_90_ & t_10_ (Seconds)		Selectivity	Operation Condition	Ref
**4 × 4 array with micro-heater**	Resistive based CNN 98.06% classification; 10.15% regression error	SnO_2_, In_2_O_3_, WO_3_, CuO, + Au decorated, 2x replicated (Total 16)	Vertical columns, Au decorated vertical columns	RF sputtering θ=85°;	**Individual**	$R_{gas}$/$R_{air}$	CNN	No CNN	N/A	250 °C; 11–15 mW	[237]
					CO (5 ppm)	<5% variation	t_90_: 1	t_90_: 112			
					NH_3_ (0.5 ppm)		t_90_: 8	t_90_: 57			
					NO_2_ (0.05 ppm)		t_90_: 5	t_90_: 174			
					CH_4_ (10 ppm)		t_90_: 19	t_90_: 44			
					C_3_H_6_O (1 ppm)		t_90_: 2	t_90_: 70			
**3 × 3 array with micro-heater**	Resistive based PCA-based gas separation	SnO_2_, WO_3_, In_2_O_3_, + Au decorated, + Bare (Total 9)	VLNs, Au-decorated thin films, thin films	E-beam θ=85°	**Individual**	ΔV response	t_90_~Fast		High vs. Acetone, Ethanol, Benzene, CO	168 °C; 800 mW heater	[215]
					H_2_S (0.534 ppb)	50–100%					
					NH_3_ (4.45 ppb)	50–100%					
					NO (0.206 ppb)	>110%					
**2 × 2 array with micro-heater**	Resistive based PCA-based gas separation PC1 + PC2 = 92.69 + 5.87 = 98.56%	SnO_2_, WO_3_, SnO_2_ @ Au, WO_3_ @ Au	VLNs, Au-decorated	E-beam θ=86°	**Individual**	$R_{gas}$/$R_{air}$	N/A		High vs. C_2_H_5_OH, CO, C_7_H_8_, C_6_H_6_, CH_3_COCH_3_	212 °C, 500 mW heater, 80% RH	[238]
					NO: (0.899 ppb)	(1 ppm) 9–133					
					NH_3_: (312 ppb)	(10 ppm) 5–20					
**4-single sensors with micro-heater**	Resistive based w/threshold decision, EWMA filtering	NiO, SnO_2_, WO_3_, In_2_O_3_	Tilted Columns	E-beam θ=80°	**Simultaneously** Fire gases from PVC (HCl, CO, VOCs)	(200 °C) NiO: 1.2 SnO_2_: 2.1 (350 °C); NiO: 577.1 SnO_2_: 294.9	Time to reach detection: SnO_2_ 1007 (200 °C); 948 (350 °C);		N/A	~250 °C,	[105]
**2 × 6 array using GFET**	Resistive + capacitive based LDA; classification enhanced with dielectric channel	TiO_2_, SnO_2_	Tilted Columns	E-beam TiO_2_, θ=85° SnO_2_θ=88°	**Individual** SO_2_, CH_2_O, C_7_H_8_, C_2_H_6_O, NO_2_, NH_3_ (1 ppm)	Linear response; distinct Z’ and tan φ traces for each gas;	N/A		N/A	175 °C, dry air, 10 mV at 8.223–13.158 kHz	[285]
**Single AAO microheater**	Resistive + temp. modulation 1D-CNN (Gasses) 97.0% MAPE: 18.0% (Spices) 96.1% classification; MAPE: 7.7–26.1%	WO_3_	Vertical Columns	RF sputtering θ=85°	**Individual** NO_2_ (0.5 ppm) Acetone (1 ppm) NH_3_ (1 ppm) Ethanol (1 ppm) **Simultaneous** Coriander, cilantro, star anise, licorice	Distinct temp-modulated pulse patterns	Detection Time ≈ 30–90		N/A	Up to 301.7 °C, 31.4 mW, 2.0–3.0 V staircase, 5.0 V at 10 Hz, dry air	[286]
**Single-μLED embedded sensor** **(photo-activated)**	Resistive based D-CNN 96.99% classification MAPE: 31.99%	In_2_O_3_	Vertical Columns	RF sputtering θ=85° E-beam Au NPs	**Individual**	(ΔR/R_0_)	t_90_ = 80 t_10_ = 79		N/A	RT, 395 nm pulse	[240]
					CH_3_OH (10 ppm)	(10 ppm) 0.56					
					EtOH (10 ppm)	(10 ppm) 0.51					
					Acetone (200 ppm)	(200 ppm) 0.17					
					NO_2_ (0.5 ppm)	(10 ppm) 2.71					
**Dual-μLED** **embedded sensor** **(photo-activated)**	Resistive based CNN 99.32% classification MAPE: 13.82%	In_2_O_3_	Vertical Columns + Au or Ag decorated	RF sputtering θ=85° E-beam Au and Ag NPs	**Individual**	$R_{gas}$/$R_{air}$	CNN detects in <30		N/A	RT, 0.38 mW total power	[243]
					CH_3_OH (10 ppm)	(100 ppm) 0.50–0.69					
					C_2_H_5_OH (10 ppm)	(100 ppm) 0.55–0.62					
					CH_3_COCH_3_ (50 ppm)	(50 ppm) 0.89–0.92					
					NO_2_ (0.5 ppm)	(5 ppm) 253–571					

**Table 5:** Summary of GLAD-based resistive gas sensors.

Mat	Structure	Deposition Condition	Post-Treating	Target Gas	Response and Sensitivity	LOD	$t_{90}\;\&\;t_{10}$	Selectivity	Operation Condition	Refs
**Single-Material**										
$SnO_{2}$	Tilted columns	DC reactive sputtering: θ=80°	350 & 500 °C/48 h in air	BTEX	0.008–0.075	30 ppb (benzene)	$t_{90}\approx 2\;min$ $t_{10}\approx 15\;min$ (8% RH, 25 °C)	Enhanced BTEX selectivity	300–500 °C tested, best ≥ 400 °C	[143]
	Vertical columns	DC Reactive sputtering: θ=87°	300 & 400 °C/3 h in air	${NO}_{2}$	5310 5 ppm@150 °C	6 ppb (400 °C)	$t_{90}=5.9\;min$ $t_{10}=2.6\;min$ (5 ppm, 150 °C)	High vs. $H_{2}$, $H_{2}S$, CO, $C_{2}H_{5}OH$	0.125–5 ppm@150 °C in air	[161]
	Vertical columns	RF sputtering: θ=50°	300 °C/ 3 h in air	CO	150 500 ppm@110 °C	50 ppm (not calc.)	$t_{90}=37\;s$ $t_{10}=160\;s$	High vs. ${CH}_{4}$, ${NH}_{3}$, $C_{2}H_{5}OH$	50–500 ppm@110 °C in dry air	[160]
$WO_{3}$	Vertical columns	RF co-sputter: θ=85°	450 °C/ 2 h in air	${NO}_{2}$	1075 10 ppm@150 °C	0.5 ppm	$t_{90}<20\;s$ $t_{10}<36\;s$	Selects oxidizing or reducing gases by temperature	${NO}_{2}$: 0.5–10 ppm $C_{2}H_{5}OH:$ 10–200 ppm @100–400 °C	[191]
				$C_{2}H_{5}OH$	10 200 ppm@300 °C	10 ppm	N/A			
	Tilted/zigzag/spiral columns	DC sputtering (GLAD + RGGP): θ=80°	300 °C/ 12 h in air	$C_{12}H_{26}$	≈60% 300 ppm@250 °C	10 ppm	$t_{90}\approx 90\;s$ $t_{10}\approx 360\;s$	N/A	250 °C	[192]
				$O_{3}$	≈35,000% 1 ppm@250 °C	0.2 ppm	$t_{90}\approx 90\;s$ $t_{10}\approx 140\;s$			
	Tilted columns	DC and RF co-sputtering: θ=80°	300 °C/ 12 h in air	$C_{12}H_{26}$	≈63% 325 ppm@500 °C	N/A	$t_{90}\approx 90\;s$ $t_{10}\approx 360\;s$	N/A	325 ppm@450–500 °C	[193]
	Vertical columns	DC sputtering: θ=85°	400–500 °C/ 3 h in air	${NO}_{2}$	≈27 2.0 ppm@250 °C	0.1 ppm	$t_{90}\approx 200\;s$ $t_{10}\approx 600\;s$ (1 ppm, 500 °C)	N/A	0.1–2 ppm @ 250 °C in air	[187]
	Villa-like Nanostructures	RF sputtering: θ=85°	500 °C/1 h in air	NO	278 @200 °C	88 ppt	$t_{90}\approx 177\;s$ $t_{10}\approx 7\;s$ (1 ppm, 200 °C)	High vs. ${NH}_{3}$, $C_{2}H_{5}OH$, CO, $C_{3}H_{6}O$	500 sccm @200 °C	[194]
	Vertical columns	DC magnetron sputtering θ=45−85°	400 °C/4 h in air	$C_{3}H_{6}O$	≈1.45 1.25 ppm@300 °C	0.1 ppm	$t_{90}\approx 140\;s$ $t_{10}\approx 457\;s$ (θ = 75°, 1.25 ppm)	N/A	300–400 °C	[195]
${In}_{2}O_{3}$	Tilted columns	E-beam evap.: θ=78−85°	550 °C/2 h in air	${CH}_{3}{COCH}_{3}$	1313	50 ppm (all gases)	$t_{90}=3\;s$	High vs. VOCs at 300 °C; Low vs. oxidizing gases	50 ppm@300 °C in dry air	[196]
				$C_{2}H_{5}OH$	1130		$t_{90}=7\;s$			
				$C_{6}H_{6}$	623		$t_{90}=7\;s$			
				HCHO	610		$t_{90}=3\;s$			
				$C_{7}H_{8}$	320		$t_{90}=7\;s$			
				${NO}_{2}$	166		$t_{90}=335\;s$			
				CO	5.03		$t_{90}=18\;s$			
				${NH}_{3}$	0.28		$t_{90}=22\;s$			
	Vertical columns	E-beam evap.: θ=80°,85°	500 °C/2 h in air	${NO}_{2}$	176 5 ppm @ 200 °C	~2 ppt	$t_{90}=20\;s$ $t_{10}=310\;s$	Tunable selectivity to redox gases	1000 sccm@200–300 °C in air	[73]
				$C_{2}H_{5}OH$	929 50 ppm@ 300 °C	~370 ppt	$t_{90}=7\;s$ $t_{10}=150\;s$			
**ZnO**	Vertical columns	RF magnetron sputter: θ=80°	N/A	${SO}_{2}$	18.19% 3 ppm@300 °C	3 ppm	$t_{90}=41.82\;s$ $t_{10}=84.93\;s$ (3 ppm)	High vs. ${NO}_{2}$ (2.75%), CO (1.45%)	300 °C in air	[197]
	Helical columns	RF sputtering: θ=85°	400 °C/2 h in air	NO	15 90 ppb@150 °C	10 ppb	$t_{90}\approx 60\;s$ $t_{10}\approx 700\;s$ 90 ppb	High vs. ${NH}_{3}$, ${CH}_{4}$, $H_{2},$ CO	150–300 °C best@250 °C	[198]
$TiO_{2}$	1-,2-,4-fold zigzag columns	Thermal evap.: θ=85°	N/A	${NH}_{3}$	3.6@50 ppm 1.17@200 ppb	0.2 ppm	$t_{90}=4.5\;s$ $t_{10}=79\;s$ (50 ppm)	High vs. ${CO}_{2}$, ${NO}_{2}$ Low vs. $H_{2}S$, $C_{2}H_{5}OH$	25 °C, 50–80%RH best@60%	[188]
**CuO**	Vertical columns	DC sputtering: θ=45−85°	400 °C/4 h in air	$C_{3}H_{6}O$	2.1 2.5 ppm@350 °C	0.25 ppm	N/A	N/A	300–400 °C best@350 °C	[129]
$V_{2}O_{5}$	Nano-sculptured thin films	DC magnetron sputtering: θ=80°	500 °C/24 h	$C_{6}H_{6}$	0.9@100 ppb	~28 ± 4 ppb	$t_{90}=75\;s$ $t_{10}=115\;s$	N/A	450 °C, ≤60% RH	[131]
**Hetero-Junction**										
$TiO_{2}/SnO_{2}$	Vertical hetero-junction columns	E-beam evap.: θ=85°	N/A	$C_{2}H_{5}OH$	~14@200 ppm, 4.7@20 ppm	20 ppm	$t_{90}^{200ppm}=41\;s$ $t_{10}^{200ppm}=84\;s$ $t_{90}^{20ppm}=37\;s$ $t_{10}^{20ppm}=64\;s$	N/A	150 °C	[199]
				$C_{3}H_{6}O$	~3@200 ppm 1.2@20 ppm		$t_{90}^{200ppm}=95\;s$ $t_{10}^{200ppm}=166\;s$ $t_{90}^{20ppm}=51\;s$ $t_{10}^{20ppm}=85\;s$			
**Decorated and Doped**										
$WO_{3}$ @Pt	Vertical columns	DC sputtering: θ=85°	450 °C/4 h in air	${NO}_{2}$	11.24@1 ppm	80 ppb	$t_{90}=27\;s$ $t_{10}=34\;s$ (1 ppm)	High vs. $NH_{3}$, CO, $C_{3}H_{6}O$, $C_{2}H_{5}OH$	150 °C; 0.08–10 ppm ${NO}_{2}$,	[113]
	Vertical columns	DC magnetron sputtering: θ=85°	400 °C/3 h in air	$H_{2}$	$2.2\times 10^{5}$ 3000 ppm@200 °C	0.5 ppm	$t_{90}\approx 8\;min$ $t_{10}\approx 47\;min$ (0.3% $H_{2}$)	High vs. $H_{2}S$, $NH_{3}$, ${NO}_{2}$, $C_{2}H_{2},$ $SO_{2},$ CO	200 °C in 2 L/min synth air	[114]
$SnO_{2}$ **@ Pd**	Vertical columns	E-beam evap. and DC sputtering: θ=70−85°	550 °C/2 h in air	$H_{2}$	104@1% in $N_{2}$ 96@480 ppm in transformer oil	$N_{2}:$ 0.2 ppm Oil: 0.3 ppm	$N_{2}$: $t_{90}=15\;s$ (1%$H_{2}$); Oil: $t_{90}=300\;s$ (480 ppm)	High vs. $C_{2}H_{2}$, CO, ${CO}_{2}$	RT in $N_{2}$; 80 °C in oil; 0.2 ppm–1% in $H_{2}$	[115]
	Vertical columns	E-beam evap.: θ=80°	550 °C/2 h in air	$C_{2}H_{2}$	0.99 10 ppm@200 °C	10 ppb	$t_{90}\approx 25\;s$ $t_{10}\approx 120\;s$	High vs. $H_{2}\;$	200 °C in dry air 0.01–50 ppm	[116]
**ZnO @ Pd**	Tilted columns	RF sputtering: θ=50−80°	N/A	CO	1020 500 ppm@150 °C	10 ppm	$t_{90}=17\;s$ $t_{10}=23\;s$	High vs. ${NO}_{2}$, ${NH}_{3}$, ${CH}_{4}$, LPG, ${CO}_{2}$.	150 °C, in dry air	[117]
$WO_{3}$ @Pd	Vertical columns:	DC sputtering: θ=85°	450 °C/4 h in air	${NO}_{2}$	2.72@1 ppm 191@10 ppm	0.5 ppm	N/A	High vs. $NH_{3}$, CO, $C_{3}H_{6}O$, $C_{2}H_{5}OH$	150 °C, 0.5–10 ppm	[118]
$SnO_{2}$ **@ Au**	“Bamboo” vertical columns w/layered Au	E-beam evap.: θ=80°	550 °C/1 h in air	$C_{2}H_{5}OH$	338.8@50 ppm	70 ppt	$t_{90}\approx 3\;s$	N/A	350 °C in 1000 sccm dry air flow	[112]
				$CH_{3}{COCH}_{3}$	301.3@50 ppm	78 ppt	$t_{90}\approx 4\;s$			
				$C_{7}H_{8}$	153.8@50 ppm	193 ppt	$t_{90}\approx 4\;s$			
$WO_{3}$ @Rh	Vertical columns	E-beam evap.: θ=80°	550 °C/2 h in air	$C_{3}H_{6}O$	75@5 ppm	~0.131 ppb	$t_{90}=11\;s$ $t_{10}=110\;s$ (5 ppm)	High vs. $C_{8}H_{10}$ $C_{2}H_{5}OH$, $C_{7}H_{8}$, $CH_{4}$	0.2–5 ppm @300 °C, 0–80% RH	[119]
${In}_{2}O_{3}$ **@ Au, Ag, Pt, Cu**	Vertical columns	RF sputtering: θ=85°	400 °C/1 h in air	$O_{2}$	2.06@0.5% O_2_ 3.34@2% O_2_	5000 ppm	$t_{90}=500\;s$ (w/o CNN) $t_{90}<5\;s$ (w/CNN);	High selectivity in humid $H_{2}$	22 °C, 30–90% RH, in $H_{2}$	[200]
$WO_{3}+C$	Vertical columns	RF sputtering: θ=85°	400 °C/3 h in air	${NO}_{2}$	320 3 ppm@250 °C	<0.5 ppm	$t_{90}\approx 17\;s$ $t_{10}\approx 50\;s$	High vs. $H_{2}$, $NH_{3}$, CO, $C_{2}H_{5}OH$, $H_{2}S$	150–250 °C best@250 °C 50 sccm in dry air	[111]

**Table 7:** Summary of GLAD-based optical gas sensors.

Mat	Structure	Deposition Condition	Target Gas	Response and Sensitivity	$t_{90}\;\&\;t_{10}$	Operating Temperature (LoD)	Ref
**Absorption**							
**Pd/** $WO_{3}$	NRs	RF sputtering: θ=85°	$H_{2}$	$∆A=51\%\;for\;0.1\%\;H_{2}$	$t_{90}\approx 60\;s,\;t_{10}\approx 90\;s$	100°C, 0.1%	[274]
${TiO}_{2}$ **/** **protonated porphyrin**	Tilted columns	E-beam evaporation θ=70°	${NH}_{3}\;$+ Amines	$∆Emission=80\%\;for\;{NH}_{3}$	$t_{90}\approx 247\;s$ $t_{10}\approx NA$	100 °C, 2.3% ${NH}_{3}$	[253]
**SERS**							
**Ag/Hf** $O_{2}$	NRs with Hf$O_{2}$ shell	E-beam evaporation θ=86°	2-NAT and 2MPy	$\Delta I\;\left(1379\;cm^{-1}\right)=$ 6000 for 600 ppb 2NAT vapor exposure	N/A	RT, 20 ppb 2-NAT	[275]
**Ag**	Tilted columns	E-beam evaporation θ=88°	4-ABT	Detection based on Raman peak intensity 4-ABT detected after 5 min exposure. Avg. signal from cryo-Ag (100 K) is 282% higher than RT	Detection ~5–60 min	RT, 5 min of 4-ABT exposure	[276]
	Tilted columns	PVD θ=86°	$H_{2}S$	BPE and Rh6G showed strong peaks at 1200–1650 cm^−1^; dye degradation monitored by SERS showed signal drop over time	N/A	RT	[277]
	Tilted columns	Thermal evaporation θ=86°	Benzene (C_6_H_6_)	Benzene Raman peak (990 cm^−1^) used for detection; signal increased by 1000× when cooled to −80 °C	$t_{90}\approx 20\;s,\;t_{10}\approx 3\;s$	RT, 1 ppb benzene @ −80 °C	[278]

**Table 9:** A comparison of different electrode configurations for resistive gas sensor design.

Configuration	Current Path	GLAD Structure Requirement	Gas Access	Fabrication Complexity	Sensor Performance Potential
**Vertical**	Through film thickness (*z*-axis)	Vertically connected porous network	Moderate (blocked by top electrode)	Moderate to High	High if well integrated
**Lateral**	Along substrate surface (x-y plane)	Lateral connectivity (tilted rods or bridging)	High	Low	Moderate to High
**Side (sandwiched)**	Across film between side electrodes	Lateral conductivity and mechanical integrity	Very High	High	High, ideal for hybrid sensing
